# Cardiac LXRα protects against pathological cardiac hypertrophy and dysfunction by enhancing glucose uptake and utilization

**DOI:** 10.15252/emmm.201404669

**Published:** 2015-07-14

**Authors:** Megan V Cannon, Herman HW Silljé, Jürgen WA Sijbesma, Inge Vreeswijk-Baudoin, Jolita Ciapaite, Bart van der Sluis, Jan van Deursen, Gustavo JJ Silva, Leon J de Windt, Jan-Åke Gustafsson, Pim van der Harst, Wiek H van Gilst, Rudolf A de Boer

**Affiliations:** 1 Department of Cardiology, University Medical Center Groningen, University of GroningenGroningen, The Netherlands; 2 Department of Nuclear Medicine, University Medical Center Groningen, University of GroningenGroningen, The Netherlands; 3 Department Pediatrics, Center for Liver, Digestive and Metabolic Diseases, University Medical Center Groningen, University of GroningenGroningen, The Netherlands; 4 Department of Pathology and Medical Biology, University Medical Center Groningen, University of GroningenGroningen, The Netherlands; 5 Department of Biochemistry & Molecular Biology, Mayo ClinicRochester, MN, USA; 6 Department of Cardiology, CARIM School for Cardiovascular Diseases, Maastricht UniversityMaastricht, The Netherlands; 7 Department of Biology and Biochemistry, University of HoustonHouston, TX, USA; 8 Department of Biosciences and Nutrition, Novum, Karolinska InstitutetHuddinge, Sweden

**Keywords:** glucose metabolism, left ventricular hypertrophy, liver X receptor, nuclear receptor, O-GlcNAcylation

## Abstract

Pathological cardiac hypertrophy is characterized by a shift in metabolic substrate utilization from fatty acids to glucose, but the molecular events underlying the metabolic remodeling remain poorly understood. Here, we investigated the role of liver X receptors (LXRs), which are key regulators of glucose and lipid metabolism, in cardiac hypertrophic pathogenesis. Using a transgenic approach in mice, we show that overexpression of LXRα acts to protect the heart against hypertrophy, fibrosis, and dysfunction. Gene expression profiling studies revealed that genes regulating metabolic pathways were differentially expressed in hearts with elevated LXRα. Functionally, LXRα overexpression in isolated cardiomyocytes and murine hearts markedly enhanced the capacity for myocardial glucose uptake following hypertrophic stress. Conversely, this adaptive response was diminished in LXRα-deficient mice. Transcriptional changes induced by LXRα overexpression promoted energy-independent utilization of glucose via the hexosamine biosynthesis pathway, resulting in O-GlcNAc modification of GATA4 and Mef2c and the induction of cytoprotective natriuretic peptide expression. Our results identify LXRα as a key cardiac transcriptional regulator that helps orchestrate an adaptive metabolic response to chronic cardiac stress, and suggest that modulating LXRα may provide a unique opportunity for intervening in myocyte metabolism.

## Introduction

Pathological cardiac hypertrophy and remodeling ensue in response to sustained elevations in hemodynamic workload (Frey & Olson, 2003). A hallmark of this remodeling process is the alteration in myocardial energy metabolism which is necessitated by increased energy demand (Neubauer, 2007; Ventura-Clapier *et al*, 2011). Under normal physiological conditions, the heart predominantly consumes fatty acids (FA), whereas various stressors trigger a compensatory shift toward glucose, the preferred substrate for its oxygen-sparing effect in ATP production (Stanley *et al*, 2005; Opie & Knuuti, 2009). Despite the success of current pharmacological strategies which aim to reduce afterload and hypertrophic growth using beta blockers and inhibitors of the renin–angiotensin–aldosterone system, heart failure nevertheless remains a progressive disease with high morbidity and mortality. Interventions in metabolic remodeling therefore represent a promising therapeutic adjunctive for targeting pathological cardiac hypertrophy and development of heart failure (Ardehali *et al*, 2012).

Liver X receptors (LXRs) are nuclear receptors that have emerged as central metabolic regulators in various organ systems and cell types. At the molecular level, LXRs function as ligand-activated transcription factors that intricately regulate and coordinate cholesterol homeostasis, glucose and lipid metabolism, and inflammatory signaling. As such, the importance of LXRs in the cardiovascular system is mainly attributed to their atheroprotective effects in accelerating reverse cholesterol transport (Naik *et al*, 2006; Zhang *et al*, 2012), and reducing atherosclerotic lesion size and inflammation (Schuster *et al*, 2002; Joseph *et al*, 2003; Giannarelli *et al*, 2012). The biological functions of LXRs are mediated via two subtypes, LXRα (NR1H3), which is predominantly expressed in liver, adipose, intestine, and macrophages, but also in heart, kidney, adrenal gland, and lung, and LXRβ (NR1H2), expressed ubiquitously (Chen *et al*, 2005). LXRs reside in the nucleus where they heterodimerize with retinoid X receptor and are bound to cognate LXR response elements (LXREs) in regulatory regions of target genes (Peet *et al*, 1998).

Less is known regarding the role of LXR in pathological cardiac hypertrophy. LXRs have been implicated in blood pressure control by regulating renin gene expression *in vivo* (Morello *et al*, 2005). In blood pressure-independent models, the LXR agonist, T0901317 (T09), attenuated the hypertrophic response to pressure overload in mice (Kuipers *et al*, 2010), whereas this effect was exacerbated in LXRα-null mice (Wu *et al*, 2009). These studies, however, are limited by confounding systemic effects of pharmacological LXR activation and by the lack of agonist specificity. Therefore, it remains unclear whether LXRα directly affects the pathogenesis of hypertrophy. The purpose of the present study was to investigate the cardiospecificity of LXRα in modulation of myocardial metabolism and pathological hypertrophy by generating a murine model of cardiac-specific LXRα overexpression.

## Results

### Mice with cardiac-specific LXRα overexpression exhibit no overt cardiac phenotype at baseline or with aging

**Figure 1:**
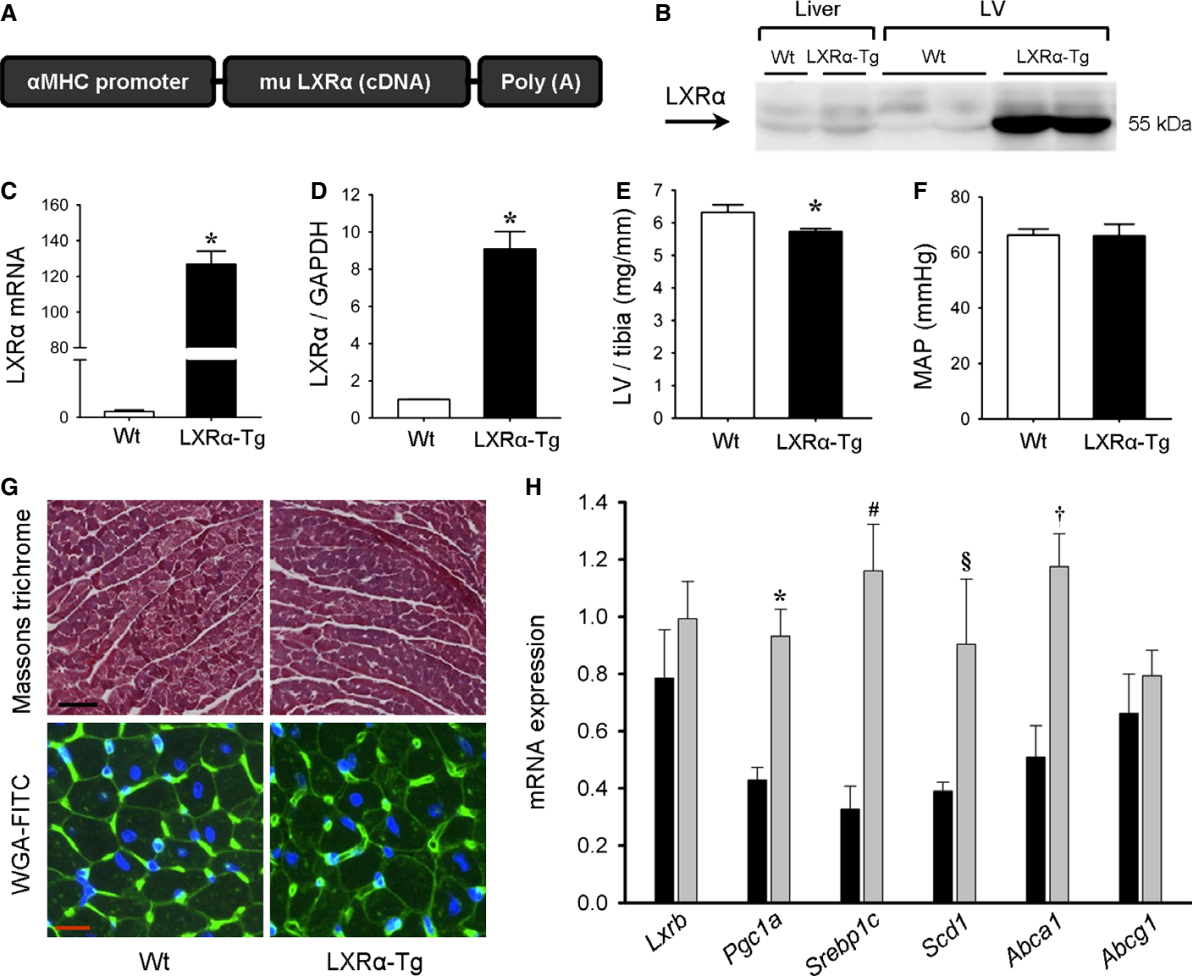
Baseline characterization of mice with cardiac-specific LXRα overexpression A Murine LXRα DNA construct for the generation of transgenic (LXRα-Tg) mice.

To study LXRα activation in the heart, we generated transgenic (LXRα-Tg) mice with cardiac-specific overexpression of murine LXRα driven by the cardiomyocyte-specific αMHC promoter (Fig1A). LXRα mRNA and protein expression were increased 130-fold and 9-fold, respectively (Fig1B, C and D). Heart weight in LXRα-Tg mice, as assessed by LV weight normalized to tibia length (LV/tibia), was marginally less compared to Wt (Fig1E). Mean arterial pressure (MAP) was unaltered in LXRα-Tg mice (Fig1F). Echocardiography-determined LV dimensions and function were comparable to Wt, and no differences in indices of contractility and intracardiac pressures, measured *in situ* with microtip catheterization, were observed (Supplementary Table S1). Histological analyses of ventricular sections stained with WGA-FITC or Masson’s trichrome displayed no evidence of abnormal cardiomyocyte morphology or collagen deposition in LXRα-Tg hearts (Fig1G). To verify whether overexpression indeed induced functionally active LXRα, we determined mRNA levels of well-described LXRα target genes (Tontonoz & Mangelsdorf, 2003) including *Srebp1c*, *Scd1*, and *Abca1*. These were significantly increased in LXRα-Tg mice (Fig1H). Furthermore, *Pgc1a*, a co-activator of LXR (Oberkofler *et al*, 2003), was also upregulated, plausibly to maintain LXRα in a constitutively active state. *Lxrb* expression was not changed. The long-term effects of cardiac-specific LXRα activation were also assessed in mice up to 12 months of age. Chronic LXRα activation did not impair gross cardiac morphology or function in aged mice (Supplementary Table S1). In summary, LXRα-Tg mice displayed normal physiological development, and all structural and functional cardiac parameters were within normal range.

B–D Left ventricular LXRα expression in Wt and LXRα-Tg mice aged 12 weeks. (B) Western blot of LXRα in liver and LV tissue. Quantification of (C) mRNA expression (normalized to *36b4*; *n* = 6–8/group, *P < 0.00001 versus Wt) and (D) protein expression (normalized to GAPDH; *n* = 4/group, *P = 0.0001 versus Wt).

E LV weight to tibia length ratios (LV/tibia); *n* = 15/group. *P = 0.02.

F Assessment of mean arterial pressure (MAP) with *in situ* catheterization shows no significant differences.

G Representative Masson’s trichrome and wheat germ agglutinin (WGA)-FITC staining in cross-sectional LV, scale bars = 100 μm and 10 μm, respectively.

H Relative mRNA levels of known LXRα-associated and target genes assessed with RT–PCR; *n* = 8/group. *P = 0.0003, ^#^*P* = 0.0004, ^§^*P* = 0.043, ^†^*P* = 0.0009 versus Wt. Data information: Data are means ± SEM; Student’s paired 2-tailed *t*-test was used to compare groups. Source data are available online for this figure.

### LXRα overexpression attenuates pathological development of cardiac hypertrophy, fibrosis, and dysfunction

**Figure 2:**
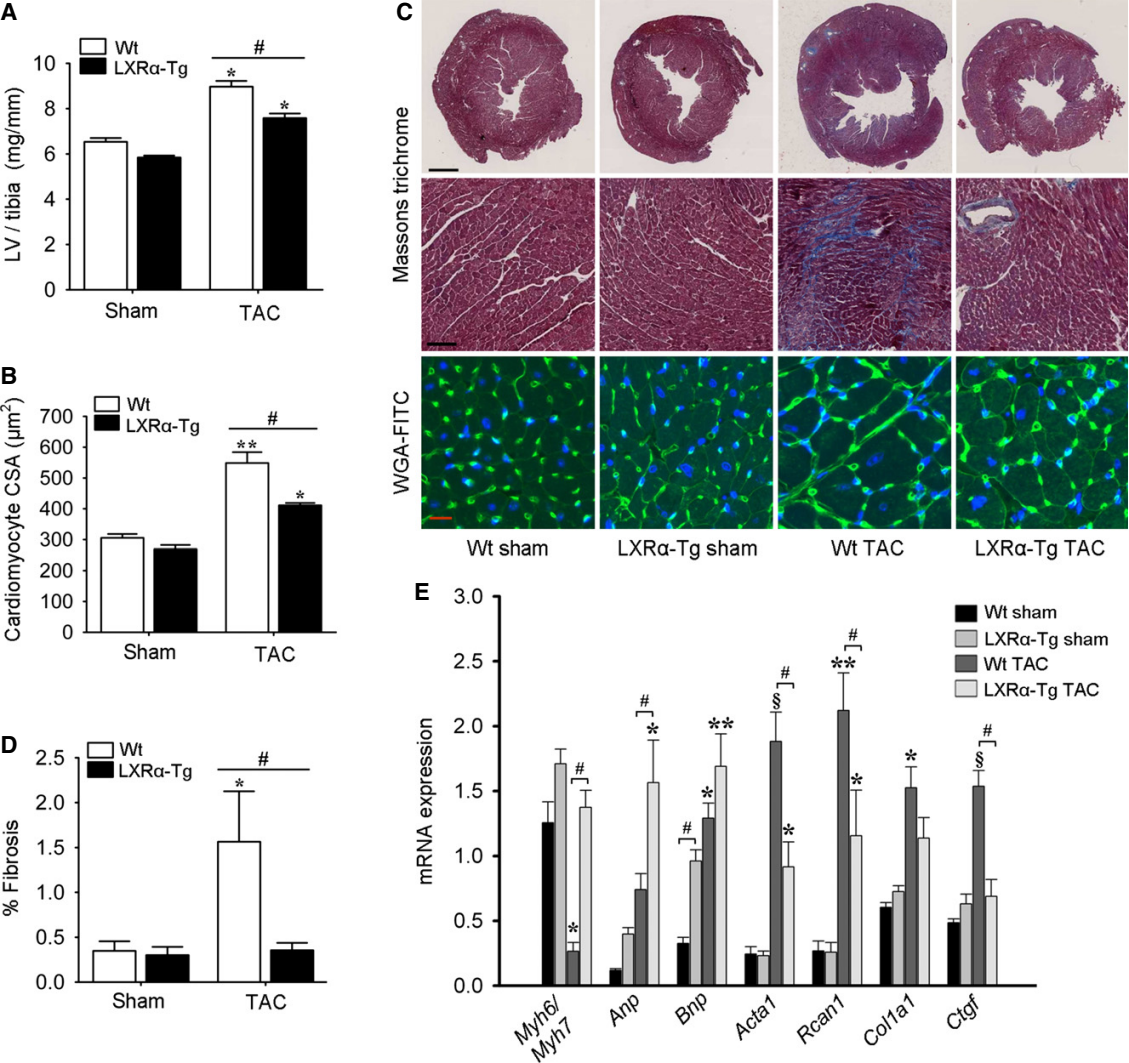
LXRα overexpression attenuates TAC-induced cardiac hypertrophy and fibrosis after 5 weeks LV/tibia ratios in sham- and TAC-operated Wt and LXRα-Tg mice; *n* = 21 Wt sham, *n* = 24 LXRα-Tg sham, *n* = 24 Wt TAC, *n* = 26 LXRα-Tg TAC. *P < 0.00001 versus respective sham, ^#^*P* = 0.00004.

To evaluate specific effects of LXRα in pathological cardiac hypertrophy, mice were subjected to pressure overload by TAC for 5 weeks. Heart weights were similar between sham-operated Wt and LXRα-Tg groups (Fig2A). TAC caused significant increases in LV/tibia ratios; however, LXRα-Tg mice exhibited 24% less hypertrophy compared to Wt, which was further evidenced by reduced cardiomyocyte size (Fig2B and C). In comparison with LXRα-Tg mice, the greater degree of hypertrophy observed in Wt was attributable to larger increases in interventricular septal and LV free wall thicknesses, while no marked dilatation of the LV chamber was observed for either TAC group (Supplementary Table S2). To further assess the impact of LXRα on other parameters of myocardial remodeling, fibrosis was quantified in cross-sectional LV. Collagen deposition was only marginally detected in LXRα-Tg hearts following TAC, whereas this increased 4-fold in Wt (Fig2C and D). These anti-fibrotic effects were associated with less induction of genes involved in fibrogenesis, *Col1a1* and *Ctgf* (Fig2E). Following TAC, typical reactivation of the fetal gene program occurred in both groups, but to a lesser extent in LXRα-Tg mice (Fig2E). Interestingly, we observed elevated basal levels of natriuretic peptides, *Anp* and *Bnp*, in LXRα-Tg mice. In contrast, other gene markers representative of fetal gene activation were more strongly induced in Wt following TAC: αMHC to βMHC isoform transition (*Myh6/Myh7* ratio), *Acta1*, as well as the hypertrophic gene, *Rcan1*.

Quantification of cardiomyocyte cross-sectional area from WGA-FITC-stained histological sections; *n* = 5 Wt sham, *n* = 4 LXRα-Tg sham, *n* = 5 Wt TAC, *n* = 3 LXRα-Tg TAC. **P = 0.00001 versus Wt sham, *P = 0.01 versus LXRα-Tg sham, ^#^*P* = 0.01.

Representative LV sections stained with WGA-FITC and Masson’s trichrome to assess cardiomyocyte hypertrophy and fibrosis, respectively; scale bars = 1 mm, 100 μm, 10 μm, from top to bottom.

Whole area percentage of fibrosis in Masson’s trichrome-stained sections; *n* = 8 per group, except *n* = 7 in Wt TAC group. *P = 0.02 versus Wt sham, ^#^*P* = 0.02.

Measurement of mRNA levels with RT–PCR to assess induction of fetal gene program, as well as hypertrophy (*Rcan1*) and fibrosis (*Col1a1, Ctgf*); *n* = 8 per group, except *n* = 7 in Wt TAC group. *Myh6/Myh7*: *P = 0.0001 versus Wt sham, ^#^*P* = 0.00001; *Anp*: *P = 0.0005 versus LXRα-Tg sham, ^#^*P* = 0.02; *Bnp*: *P = 0.0006 versus Wt sham, **P = 0.009 versus LXRα-Tg sham, ^#^*P* = 0.03; *Acta1*: ^§^*P* < 0.00001 versus Wt sham, *P = 0.01 versus LXRα-Tg sham, ^#^*P* = 0.001; *Rcan1*: **P = 0.00004 versus Wt sham, *P = 0.05 versus LXRα-Tg sham, ^#^*P* = 0.04; *Col1a1*: *P = 0.00003 versus Wt sham; *Ctgf*: ^§^*P* < 0.00001 versus Wt sham, ^#^*P* = 0.00001. Data information: Data are means ± SEM; one-way ANOVA with Bonferroni’s multiple comparison test was used to compare groups.

**Figure 3:**
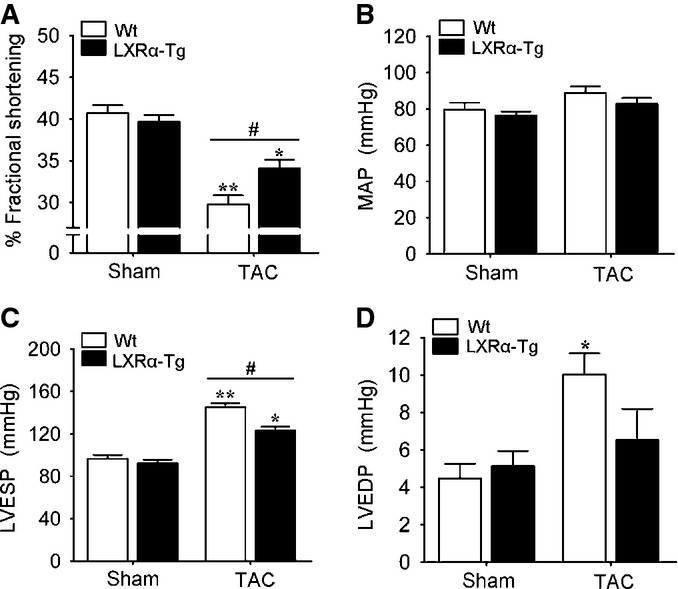
Reduced cardiac dysfunction in mice with cardiac LXRα activation in response to pathological pressure overload A Echocardiographic assessment of percent fractional shortening in mice subjected to 5 weeks of TAC; *n* = 19 Wt sham, *n* = 22 LXRα-Tg sham, *n* = 24 Wt TAC, *n* = 26 LXRα-Tg TAC. **P < 0.00001 versus Wt sham, *P = 0.0005 versus LXRα-Tg sham, ^#^*P* = 0.009.

Functional evaluation revealed that Wt mice subjected to TAC achieved significantly less systolic LV thickening and demonstrated greater acceleration toward heart failure as fractional shortening declined 11% versus only 6% in LXRα-Tg (Fig3A). These functional improvements in LXRα-Tg mice were further accompanied by reduced intracardiac pressures (Fig3C and D). No changes in MAP or HR were recorded (Fig3B, Supplementary Table S2).

B–D Hemodynamic measurements obtained *in situ* in sham- and TAC-operated mice; *n* = 6–8/group. (B) Mean arterial pressure (MAP) showed no significant differences. (C) LV end-systolic pressure (LVESP). **P < 0.00001 versus Wt sham, *P = 0.00004 versus LXRα-Tg sham, *P = 0.003. (D) LV end-diastolic pressure (LVEDP). *P = 0.02 versus Wt sham. Data information: Data are means ± SEM; one-way ANOVA with Bonferroni’s multiple comparison test was used to compare groups.

To determine whether cardiac LXRα overexpression affects early hypertrophic remodeling processes, mice were subjected to 1 week of TAC-induced pressure overload. Cardiac hypertrophy was present in Wt mice after 1 week of TAC; however, this was significantly attenuated in LXRα-Tg mice (Supplementary Fig S1A). Assessment of cardiac function with echocardiography indicated that function remained relatively compensated in TAC-operated mice (Supplementary Fig S1B). The effect of cardiac LXRα on hypertrophy, including expression of fetal genes (Supplementary Fig S1C), is comparable to what we observed in TAC after 5 weeks. Molecular determinants of inflammation were more strongly upregulated in Wt hearts compared to LXRα-Tg (Supplementary Fig S1D–G), whereas the anti-apoptotic factor, Bcl2, was significantly induced with LXRα overexpression (Supplementary Fig S1I and J). These data implicate an anti-inflammatory role for LXRα in the initial phase of hypertrophic pathogenesis, and possible protection against anti-apoptotic triggers.

To further verify that our observations were not model dependent, we conducted experiments with Ang II infusion over 10 days. Consistent with our findings from TAC experiments, LXRα-Tg mice showed reduced Ang II-induced myocardial hypertrophy and fibrosis with moderate improvements in hemodynamic parameters (Supplementary Fig S2). Taken together, these data demonstrate that cardiac-specific LXRα activation ameliorates adverse cardiac remodeling and dysfunction in mice in response to diverse pathological hypertrophic stimuli.

### LXRα overexpression induces transcriptional alterations in metabolic pathways

**Figure 4:**
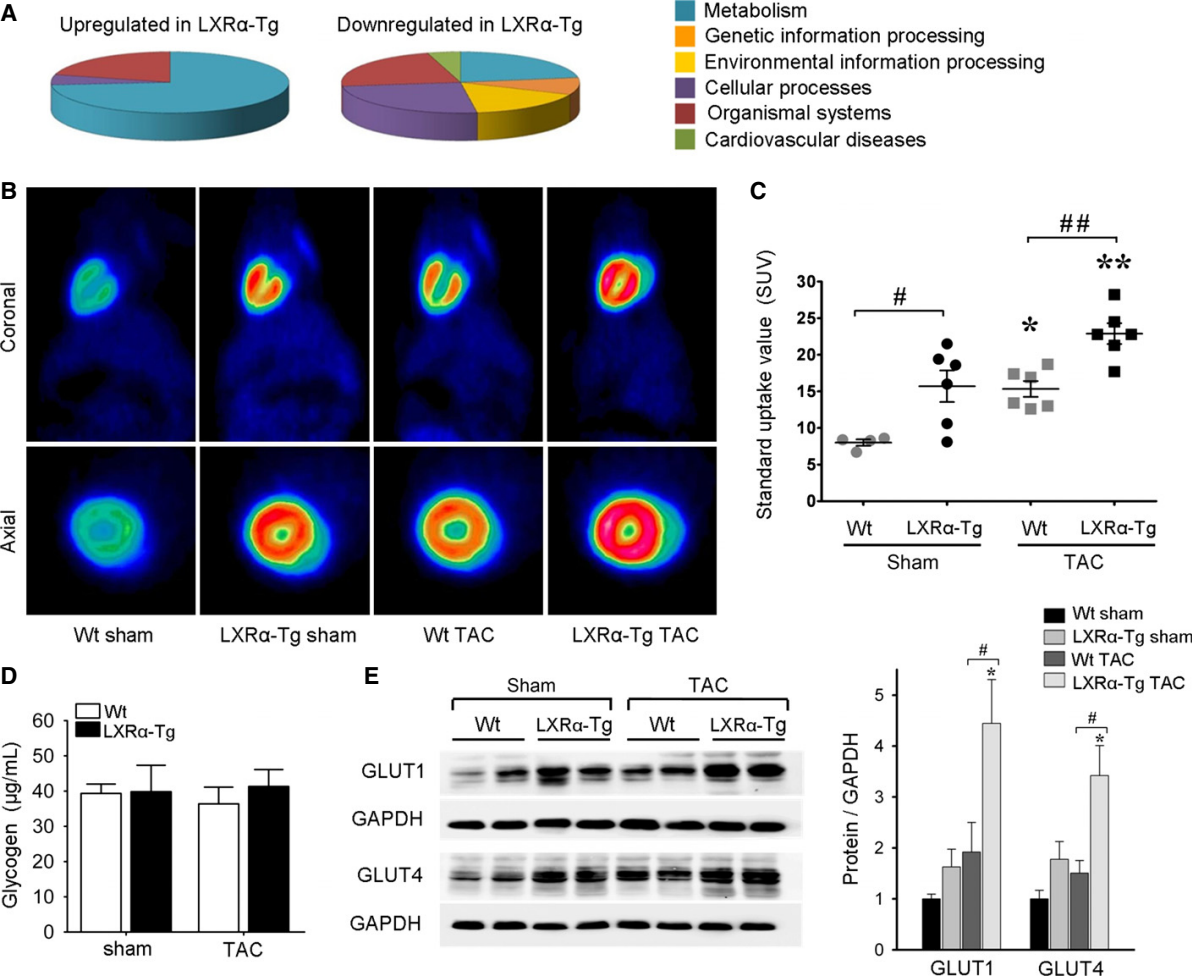
Cardiac LXRα overexpression enhances myocardial glucose uptake Classification of genes differentially expressed in LXRα-Tg hearts (see Supplementary Fig S3).

Gene profiling of the LV transcriptome was performed to uncover the molecular basis for the cardioprotective phenotype observed in LXRα-Tg mice (Supplementary Fig S3, Supplementary Table S3). Basal LXRα overexpression induced substantial changes in genes relating to metabolism (Fig4A). To further investigate microarray pathway analysis, mRNA levels of several key genes regulating FA and glucose metabolism were determined (Supplementary Fig S4). In LXRα-Tg hearts, glycolysis-related genes were more differentially expressed, including upregulation of the glucose transporters, *Glut1* and *Glut4*, as well as *Pdk4,* regulating pyruvate oxidation in mitochondria.

Representative ^18^F-FDG images with microPET in the coronal and axial planes in Wt and LXRα-Tg mice 5 weeks post-TAC.

Myocardial FDG uptake measured as standard uptake value (SUV); *n* = 4–6/group. *P = 0.04 versus Wt sham, **P = 0.02 versus LXRα-Tg sham, ^#^*P* = 0.03, ^##^*P* = 0.01.

Quantification of myocardial glycogen content shows no significant differences; *n* = 5 per sham group, *n* = 7 per TAC group.

GLUT protein expression in LV tissue normalized to GAPDH; *n* = 6/group. Glut1: *P = 0.01 versus LXRα-Tg sham, ^#^*P* = 0.03; Glut4: *P = 0.03 versus LXRα-Tg sham, ^#^*P* = 0.01. Data information: Data are means ± SEM; one-way ANOVA with Bonferroni’s multiple comparison test was used to compare groups. Source data are available online for this figure.

TAC provoked parallel transcriptional alterations in Wt and LXRα-Tg mice, downregulating FA metabolism and similarly upregulating pathways pertaining to extracellular remodeling and cardiovascular disease. However, the comparison between differentially expressed genes in hypertrophic hearts was most striking for LXRα-Tg where more than 50% of upregulated genes clustered into metabolic pathways, for example, glutathione metabolism. Collectively, these expression data convey that LXRα activation transcriptionally reprograms metabolic pathways in the heart, specifically glucose metabolism.

### Constitutive LXRα activation enhances myocardial glucose uptake and utilization

We next evaluated whether global transcriptional changes relating to glucose metabolism translated into a functional metabolic outcome. To this end, *in vivo* glucose uptake measurements were performed in a separate sham/TAC cohort (*n* = 22) by injecting mice with FDG and using microPET imaging modality. Basal myocardial FDG-glucose uptake was 1.9-fold higher in LXRα-Tg mice compared to Wt, indicating a greater propensity for glucose utilization. Consistent with previous reports showing a substrate shift to glucose following hypertrophic perturbation (Liao *et al*, 2002; Voelkl *et al*, 2012), TAC lead to a substantial increase in FDG-glucose uptake in both groups. Wt mice achieved 90% increase, which subsequently matched basal LXRα-Tg levels. On the other hand, TAC-operated LXRα-Tg mice exhibited comparatively even greater capacity for glucose uptake that was augmented 50% above both basal LXRα-Tg levels and that of Wt TAC cohorts (Fig4B and C). Increased glucose uptake in LXRα-Tg hearts did not impact systemic blood glucose levels as no significant differences in basal levels prior to scan, nor under fasted conditions (Supplementary Table S1), were observed. Furthermore, enhanced glucose uptake was not stored, but rather utilized since myocardial glycogen content was unaltered (Fig4D).

Expression levels of several key proteins involved in substrate metabolism and regulation were measured. In concert with FDG-glucose uptake levels, GLUT1 and GLUT4 protein levels were increased in LXRα-Tg hearts (Fig4E), suggesting their membrane translocation and functionality. No appreciable differences between LXRα-Tg and Wt were observed for hexokinase 2 (HK2), the enzyme catalyzing the first step in glycolysis, nor phosphorylated-AMPK, a key metabolic regulator in response to increased workload and energetic stress, as well as for CD36 regulating myocardial FA uptake (Supplementary Fig S5). In summary, enhanced myocardial glucose uptake evidenced in LXRα-Tg mice in the stressed and non-stressed state is associated with induction of GLUT1 and GLUT4.

### LXRα-deficient mice manifest reduced myocardial glucose uptake capacity in response to TAC

**Figure 5:**
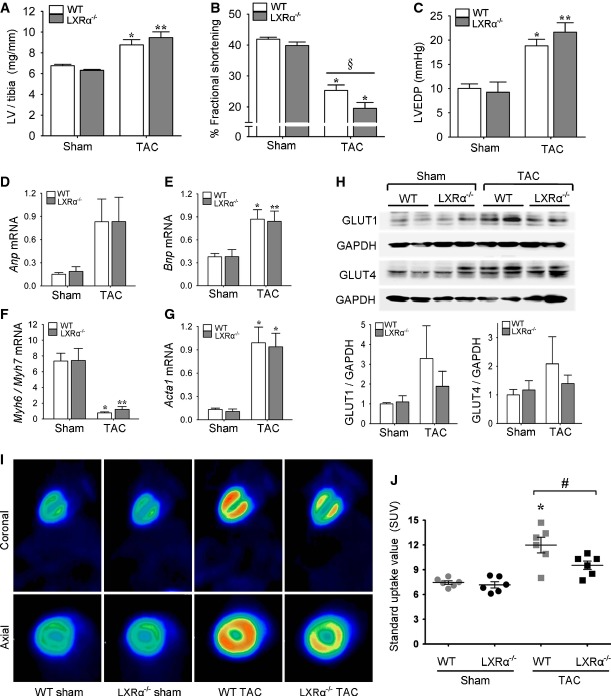
LXRα-null mice manifest reduced myocardial glucose uptake capacity in response to TAC A LV/tibia ratios in sham- and TAC-operated WT and LXRα^−/−^ mice; *n* = 10 WT sham, *n* = 8 LXRα^−/−^ sham, *n* = 12 WT TAC, *n* = 10 LXRα^−/−^ TAC. *P = 0.006 versus WT sham, **P = 0.00008 versus LXRα^−/−^sham.

To gain further insight into LXRα regulation of myocardial metabolism in cardiac hypertrophy, loss-of-function studies were performed in LXRα^−/−^ mice (Supplementary Table S4). Following 5 weeks of pressure overload, both WT and LXRα^−/−^ mice developed LV hypertrophy (Fig5A). The relative increases in LV/tibia ratios were considerably higher for LXRα^−/−^ compared to WT, 50% versus 30%, albeit not significantly different from each other. Furthermore, LXRα-deficient hearts exhibited a greater tendency toward cardiac dysfunction (Fig5B and C). Activation of the fetal gene program occurred to a similar extent in both LXRα^−/−^ and WT mice (Fig5D–G).

B, C Cardiac functional assessment at 5 weeks post-TAC. (B) Percent fractional shortening determined with echocardiography; *n* = 10 WT sham, *n* = 7 LXRα^−/−^ sham, *n* = 12 WT TAC, *n* = 10 LXRα^−/−^ TAC. *P < 0.00001 versus respective sham, ^§^*P* = 0.06. (C) LV end-diastolic pressure (LVEDP) recorded *in situ*; *n* = 10 WT sham, *n* = 7 LXRα^−/−^ sham, *n* = 10 WT TAC, *n* = 9 LXRα^−/−^ TAC. *P = 0.001 versus WT sham, **P = 0.00005 versus LXRα^−/−^ sham.

D–G Relative mRNA gene expression as determined by RT–PCR, normalized to *36b4*; *n* = 8/group. *Anp*: no significant differences; *Bnp*: *P = 0.01 versus WT sham, **P = 0.03 versus LXRα^−/−^ sham; *Myh6/Myh7*: *P = 0.0002 versus WT sham, **P = 0.0003 versus LXRα^−/−^ sham; *Acta1*: *P = 0.001 versus respective sham.

H Western blot detection of GLUT protein expression in LV tissue normalized to GAPDH, expressed as fold change, shows no significant differences among groups; *n* = 6–8/group.

I Representative ^18^F-FDG-microPET scans.

J Myocardial FDG uptake quantified as standard uptake value (SUV); *n* = 6/group. *P = 0.0001 versus WT sham, ^#^*P* = 0.047. Data information: Data are means ± SEM; one-way ANOVA with Bonferroni’s multiple comparison test was used to compare groups. Source data are available online for this figure.

The metabolic response of LXRα-null hearts to hypertrophic perturbation was assessed by evaluating myocardial glucose uptake and GLUT expression. Both GLUT1 and GLUT4 were evidently less upregulated in LXRα^−/−^ hearts in response to TAC (Fig5H). Furthermore, FDG-glucose analysis with microPET revealed that LXRα^−/−^ mice demonstrated an inability to normalize the increases in glucose uptake levels achieved in pressure overload-induced hypertrophy (Fig5I and J, Supplementary Fig S6). Myocardial glucose uptake increased 1.6-fold in WT, but only 1.3-fold in LXRα^−/−^ mice. Overall, loss of LXRα resulted in a more progressive deterioration of function following TAC that was associated with a compromised adaptive capacity to augment glucose uptake.

### Mitochondrial oxidative capacity of pyruvate is unaltered by chronic LXRα overexpression

To assess whether LXRα-Tg mice displayed increased mitochondrial capacity to oxidize glucose-derived substrates, we determined oxidative phosphorylation in permeabilized LV muscle fibers in the presence of pyruvate in an additional sham/TAC cohort (*n* = 26). Basal (state 2) and maximal ADP-stimulated (state 3) oxygen consumption rates did not differ among stressed and unstressed LXRα-Tg and Wt hearts (Supplementary Fig S7A), indicating that the capacity for pyruvate oxidation was neither impaired nor enhanced by LXRα overexpression or by TAC. Citrate synthase activity, a marker of mitochondrial density to which all respirometry measurements were normalized, was similar for all groups (Supplementary Fig S7B).

Interestingly, we recorded increased state 2 and state 3 respiration rates with palmitoyl (C16)-carnitine in LXRα-Tg, suggesting a trend toward increased capacity to oxidize FA (Supplementary Fig S7C). This may be due to a reciprocal effect on pyruvate oxidation by *Pdk4*, which was induced in LXRα-Tg (Supplementary Fig S4A). The respiratory control ratio, indicative of overall mitochondrial function (Brand & Nicholls, 2011), tended to be higher for palmitoyl-carnitine in stressed and unstressed hearts overexpressing LXRα (Supplementary Fig S7D). Further lipid profiling revealed increased myocardial leanness in LXRα-Tg hearts (Supplementary Fig S8), despite induction of several lipogenic gene targets (*Srebp1c*, *Scd1, Fasn)* (Fig1H, Supplementary Fig S4B).

### LXRα-mediated glucose uptake increases O-GlcNAc signaling in cardiomyocytes

**Figure 6:**
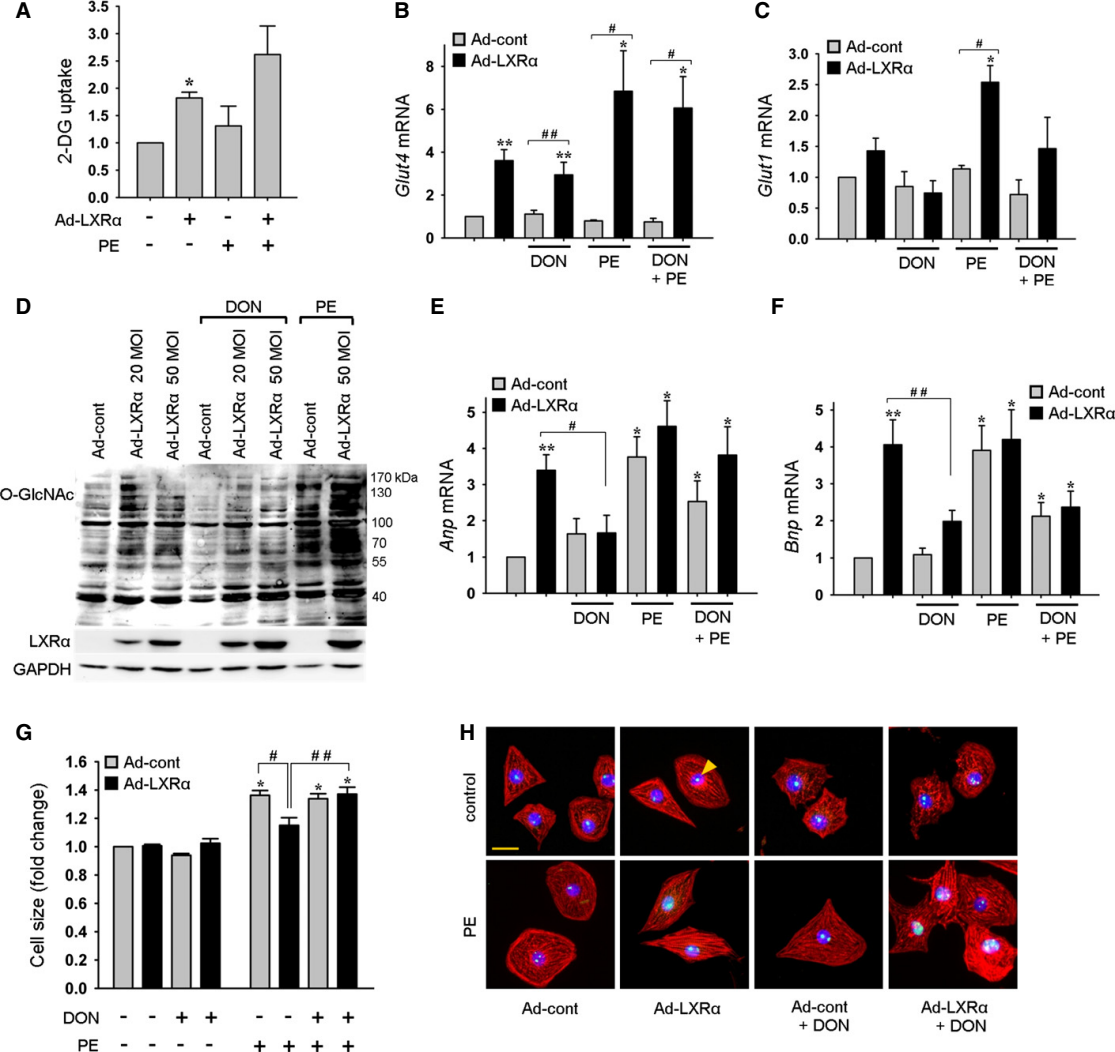
LXRα enhances glucose uptake and O-GlcNAc signaling via activation of the hexosamine biosynthetic pathway (HBP) in cultured cardiomyocytes Neonatal rat ventricular myocytes were transfected with Ad-LXRα or GL2 (Ad-cont), and treatments with phenylephrine (PE) and DON (inhibitor of HBP) were initiated for 24 h.
A Assessment of 2-deoxyglucose (2-DG) uptake from 4 independent experiments. *P = 0.03 versus Ad-cont.

Since no differences in mitochondrial capacity to utilize pyruvate was identified, we postulated that beneficial effects derived from LXRα-mediated enhanced glucose uptake involved alternate pathways of glycolytic metabolism. One such pathway is the hexosamine biosynthesis pathway (HBP) which culminates in the formation of O-GlcNAc, a posttranslational modifier of numerous proteins. The HBP has been demonstrated to be an essential signaling system in the failing heart (Watson *et al*, 2010), and accumulating evidence from *in vitro* and *ex vivo* systems shows that augmented O-GlcNAc levels via the HBP potentiates cytoprotection (Ngoh *et al*, 2010; Darley-Usmar *et al*, 2012). Using NRVMs, we tested the hypothesis that LXRα-mediated increases in glucose uptake would enhance substrate availability for O-GlcNAc. NRVMs transfected with Ad-LXRα showed significantly elevated 2-deoxyglucose (2-DG) levels of 1.8-fold compared to Ad-cont cells, which was further augmented 40% following PE stimulation (Fig6A). *Glut4* and *Glut1* mRNA levels were correspondingly increased (Fig6B and C). Next, we assessed whether enhanced glucose availability led to HBP activation and downstream formation of O-GlcNAc. Ad-LXRα cells displayed increased global protein O-GlcNAcylation that was further enhanced with PE (Fig6D). Administration of DON to inhibit HBP flux attenuated Ad-LXRα-increased O-GlcNAcylation (Fig6D), confirming the link between LXRα and HBP-O-GlcNAc signaling.

B, C *Glut* mRNA expression determined by RT–PCR normalized to *36b4*, *n* = 5 per condition in the absence of PE, *n* = 4 per condition in the presence of PE. *P = 0.02 versus Ad-cont, **P = 0.008 versus Ad-cont, ^#^*P* = 0.03, ^##^*P* = 0.008.

D Western blot indicating Ad-LXRα- and PE-induced increases in global protein O-GlcNAcylation, which was abrogated following inhibition of HBP with DON. LXRα protein expression is shown, and GAPDH served as a loading control.

E, F Modulation of *Anp* and *Bnp* mRNA levels by Ad-LXRα-induced O-GlcNAc signaling. Gene expression as determined by RT–PCR normalized to *36b4*, *n* = 5 per condition in the absence of PE, *n* = 4 per condition in the presence of PE. *P = 0.02 versus Ad-cont, **P = 0.008 versus Ad-cont, ^#^*P* = 0.03, ^##^*P* = 0.02.

G Measurement of cell size, *n* = 5 per condition. *P = 0.008 versus Ad-cont, ^#^*P* = 0.02, ^##^*P* = 0.03.

H Representative images for the determination of cell size. Cells were stained with an antibody specific for LXRα (green, indicated by arrow), DAPI for nuclei (blue), and rhodamine-phalloidin for *F*-actin (red); scale bar = 50 μm. Data information: Data are means ± SEM and are reported as fold change with respect to control group; Kruskal–Wallis test followed by Mann–Whitney *U*-test was used for group comparisons. Source data are available online for this figure.

From our findings in LXRα-Tg mice, we speculated that increased myocardial natriuretic peptide expression (Fig2E) in conjunction with preference for glucose may, in part, be evidence of an endogenous cardioprotective stress response elicited via LXRα overexpression. The anti-hypertrophic properties of ANP and BNP are well established (Nishikimi *et al*, 2006). Ad-LXRα cells expressed both increased *Anp* and *Bnp* (3.2-fold and 3.8-fold, respectively), which were subsequently suppressed following DON inhibition, suggesting that their induction is linked to O-GlcNAc effector signaling (Fig6E and F). Further assessment of cellular hypertrophy indicated that DON inhibition of HBP flux also abolished the Ad-LXRα-mediated reductions in cell size that was increased upon PE stimulation (Fig6G and H).

**Figure 7:**
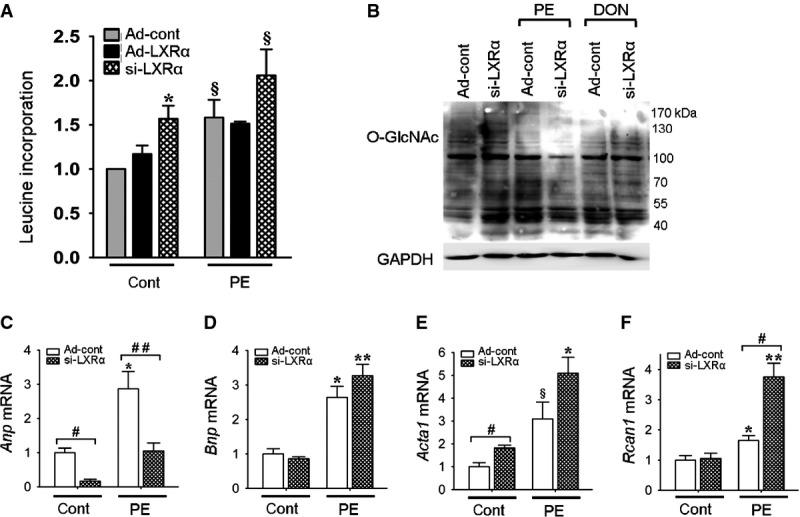
Knockdown of LXRα increases cellular hypertrophy in cultured cardiomyocytes Cells were transfected with Ad-LXRα, si-LXRα, or Ad-cont, in the absence or presence of phenylephrine (PE).
A Protein synthesis determined via leucine incorporation, *n* = 2–4 independent experiments. *P = 0.03 versus Ad-cont, ^§^*P* = 0.06 versus Ad-cont.

Alternative experiments were performed with si-LXRα to address the causal relationship among LXRα expression, protein O-GlcNAc modification, and hypertrophy. Knockdown of LXRα led to comparatively higher levels of hypertrophic growth (Fig7A), and lower levels of O-GlcNAc following PE-induced cellular stress (Fig7B). Interestingly, gene expression analysis (Fig7C–F) revealed significant downregulation of *Anp* in LXRα-silenced cells (Fig7C).

B Global O-GlcNAc protein expression with Western blot, GAPDH served as a control.

C–F mRNA expression, normalized to *36b4*, *n* = 6 per condition, except *n* = 5 for si-LXRα control. *Anp*: *P = 0.009 versus Ad-cont, ^#^*P* = 0.004, ^##^*P* = 0.009; *Bnp*: *P = 0.004 versus Ad-cont, **P = 0.002 versus Ad-cont; *Acta1*: *P = 0.002 versus Ad-cont, §*P* = 0.06 versus Ad-cont, ^#^*P* = 0.02; *Rcan1*: *P = 0.03 versus Ad-cont, **P = 0.004 versus Ad-cont, ^#^*P* = 0.004. Data information: Data are means ± SEM and are reported as fold change with respect to control group; Kruskal–Wallis test followed by Mann–Whitney *U*-test was used for group comparisons. Source data are available online for this figure.

### Transcriptional activators of natriuretic peptides are O-GlcNAc modified in LXRα-Tg hearts

**Figure 8:**
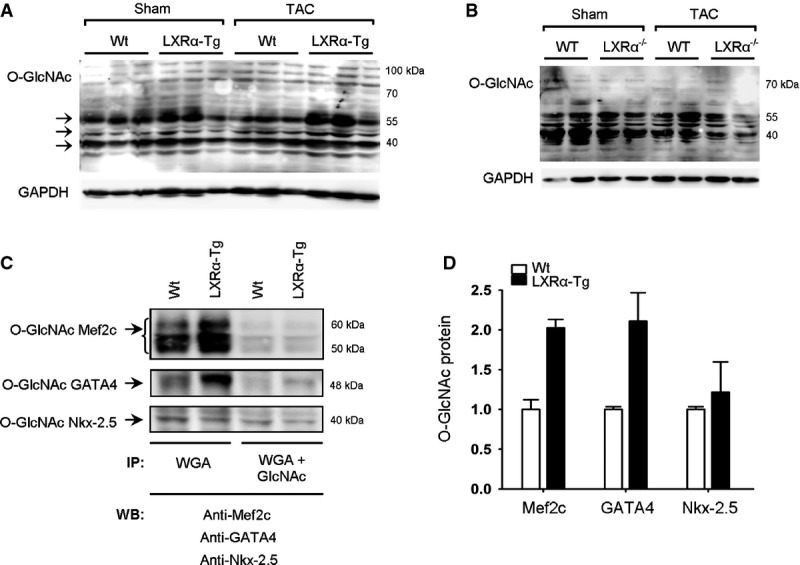
O-GlcNAcylation is increased with cardiac LXRα overexpression in mice A, B Western blot analyses of global protein O-GlcNAc levels in left ventricles of mice with either (A) cardiac-specific LXRα overexpression or (B) LXRα deficiency and subjected to 5 weeks TAC.

Finally, to corroborate our *in vitro* findings, we assessed global protein O-GlcNAc levels from LV tissue lysates of mice overexpressing and deficient for LXRα. Most extensive O-GlcNAcylation was observed in LXRα-Tg hearts involving proteins between 40 and 55 kDa in size (marked in Fig8A). In contrast, loss of LXRα resulted in attenuated O-GlcNAc signal in response to TAC (Fig8B). To further identify specific O-GlcNAc targets, agarose wheat germ agglutinin (WGA) precipitation was performed to isolate nuclear GlcNAcylated proteins. Using antibodies specific for known transcription factors activating ANP and BNP (Morin *et al*, 2000; Hayek & Nemer, 2011), Western blot analysis revealed that GATA4 and Mef2c precipitated with WGA in LXRα-Tg hearts, but not with Nkx-2.5, suggesting that O-GlcNAc modification of GATA4 and Mef2c potentiates their activities (Fig8C and D). *N*-acetylglucosamine (GlcNAc), a competitor, served as a control.

C, D Nuclear protein extracts were precipitated with agarose WGA in the absence or presence of GlcNAc, a competitor, and analyzed by Western blot with antibodies against Mef2c, GATA4, or Nkx-2.5, known transcription factors of natriuretic peptides; bands represent 3 pooled hearts per Wt and LXRα-Tg lanes, and (D) quantification is for *n* = 2, expressed as fold change. Data information: Data are means ± SEM. IP, immunoprecipitation; WGA, wheat germ agglutinin; GlcNAc, *N*-acetylglucosamine; WB, Western blot. Source data are available online for this figure.

**Figure 9:**
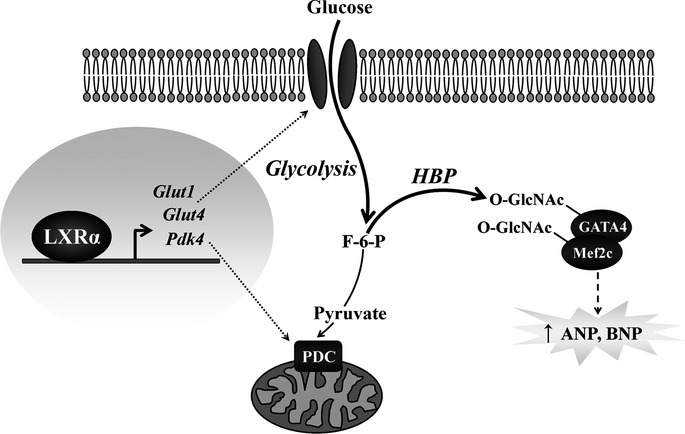
Schematic representation of LXRα-mediated glucose protection LXRα transcriptionally regulates glycolytic metabolism by targeting glucose utilization at distinct levels. Increased *Glut1* and *Glut4* enhance glucose uptake, and *Pdk4* regulates pyruvate oxidation in mitochondria. Subsequent increases in glycolytic flux activate the HBP, resulting in downstream O-GlcNAc modification of transcription factors inducing natriuretic peptide expression, a putative end effector mediating anti-hypertrophic effects in the heart. ANP, atrial natriuretic peptide; BNP, B-type natriuretic peptide; F-6-P, fructose-6-phosphate; Glut, glucose transporter; HBP, hexosamine biosynthesis pathway; Mef2c, myocyte enhancer factor 2C; O-GlcNAc, β-*O*-linkage of *N*-acetylglucosamine; PDC, pyruvate dehydrogenase complex; Pdk4, pyruvate dehydrogenase kinase 4.

In summary, these data indicate that cardiac LXRα integrates glucose metabolism and downstream O-GlcNAcylation with induction of cytoprotective natriuretic peptides to orchestrate an anti-hypertrophic response. Therefore, the energy-independent effects of glucose that, herein, are governed by LXRα may be an important salutary mechanism in modulating and preserving myocyte function (Fig9).

## Discussion

In the present study, we describe a cardiac-specific overexpression model for LXRα in mice, and herewith, elucidate the significance of LXRα in modulating myocardial metabolism in pathological hypertrophy. We established that constitutive LXRα activation in murine hearts substantially diminished LV hypertrophy, adverse cardiac remodeling, and improved overall cardiac function following chronic pressure overload and Ang II stimulation. Using this model, we identified the intrinsic transcriptional regulatory mechanisms LXRα exerts in the heart and in counteracting hypertrophic stress. By principally modulating glucose pathways, LXRα functionally enhanced the capacity for myocardial glucose uptake, which was conversely impaired in hypertrophic hearts deficient for LXRα. Furthermore, increased glucose utilization via an energy-independent pathway resulted in the glycosylation of transcription factors inducing natriuretic peptide expression, which we identified as a putative end effector of LXRα-mediated protective effects in the heart.

The role of LXRα in protection against cardiac pathophysiology is not well established. This has been previously addressed in pharmacological studies using the LXR agonist, T09, and LXRα-null mice (Wu *et al*, 2009; Kuipers *et al*, 2010). Conceivably, these approaches are restrained by confounding variables associated with systemic LXR activation, which include lipogenic (Peet *et al*, 1998), anti-inflammatory (Zelcer & Tontonoz, 2006), and blood pressure lowering (Leik *et al*, 2007) effects. Moreover, T09 mediates its effects indiscriminately via other nuclear receptors since it is also a co-activator of farnesoid X receptor, pregnane X receptor, and retinoic acid receptor signaling (Houck *et al*, 2004; Mitro *et al*, 2007; Kumar *et al*, 2010). Selectively overexpressing cardiac LXRα in mice circumvented these confounding factors and afforded a system for delineating the heart-specific effects of LXRα. Using two diverse hypertrophic perturbations, we demonstrated that constitutive LXRα activation countervailed pathological growth and remodeling processes in the heart, including blunting the development of myocardial fibrosis, an observation in line with previous studies demonstrating the anti-fibrotic effects of LXRs in kidney (Tachibana *et al*, 2012) and in liver (Beaven *et al*, 2011). Cardiac LXRα also appears to influence early remodeling processes since less inflammation in association with decreased hypertrophy occurred at an earlier time point of 1 week post-TAC. LXRα-Tg mice may also be less susceptible to apoptosis, which is underscored by upregulation of Bcl2. Taken together, counteraction of inflammatory signaling and myocyte death may explain the attenuated development of fibrosis remodeling we observed after 5 weeks TAC.

In loss-of-function studies, LXRα^−/−^ mice did not develop significantly greater severity of hypertrophy with respect to WT, although function was worsened in LXRα-deficient hearts. This is in contrast to a previous report showing exacerbated hypertrophic response in LXRα^−/−^ mice (Wu *et al*, 2009). The discrepancy between LXRα overexpression and deficiency cannot be fully explained herein; however, our *in vitro* data indicate that, at the cellular level, there is a clear effect on hypertrophic growth in cardiomyocytes lacking LXRα, and thus, compensatory mechanisms may be operative in the intact heart. Interestingly, recent evidence from a genome-wide association study (GWAS) of electrocardiographic LV hypertrophy (LVH) found a genetic variant, or SNP, in the LXRα (*Nr1h3*) locus to be significantly associated with the LVH trait. Furthermore, expression QTL analysis showed a significant correlation between decreased expression of *Nr1h3* and increased LVH (P. Van der Harst, unpublished data, 2011), supporting an anti-hypertrophic role for LXRα.

Functionally, gene profiling in our model of cardiac-specific LXRα overexpression identified primary effects for LXRα on metabolic pathways, and further investigation into this metabolic profile revealed that myocardial glucose uptake in association with GLUT expression was significantly increased in LXRα-Tg hearts and in isolated cardiomyocytes. GLUT1 and GLUT4 have previously been elucidated as targets for transcriptional regulation by LXRs in adipose tissue and skeletal muscle (Ross *et al*, 2002; Dalen *et al*, 2003; Laffitte *et al*, 2003; Kase *et al*, 2005; Griesel *et al*, 2010). Myocardial glucose uptake capacity was enhanced in LXRα-Tg mice, and more importantly, when challenged with hypertrophic stress, these mice demonstrated an even more robust response versus that of Wt. Conversely, insufficient glucose uptake capacity that ensued in hypertrophic hearts deficient for LXRα resulted in a worsened functional outcome. The shift toward greater glucose reliance is believed to be an adaptive response that confers cardioprotection (Opie & Knuuti, 2009; Kolwicz & Tian, 2011), and evidence from other genetic mouse models renders further support for a role for glucose uptake in myocardial protection. Cardiac-specific overexpression of GLUT1 in mice increased glucose uptake and glycolysis which prevented the development of ventricular dysfunction and improved survival (Liao *et al*, 2002; Luptak *et al*, 2005), whereas reduced glucose utilization in GLUT4 knockout mice manifested greater hypertrophy and acceleration toward heart failure (Katz *et al*, 1995; Domenighetti *et al*, 2010). With cardiac insulin resistance and metabolic dysregulation known to precede the development of heart failure (Witteles & Fowler, 2008; Brouwers *et al*, 2013), strategies sensitizing the heart to glucose uptake may thus have clinically relevant implications in the long-term prognosis of heart failure.

Since glucose uptake rates were enhanced by LXRα activation, we hypothesized that this would also lead to downstream changes in energy-dependent pathways, causing increased mitochondrial oxidative capacity. However, we did not observe corresponding increases in oxidative capacity from pyruvate, suggesting that LXRα does not transcriptionally reprogram pathways for the enhancement of mitochondrial glucose utilization. In essence, excess glucose uptake and glycolysis appears to be partially uncoupled from mitochondrial oxidation and ATP synthesis in LXRα-Tg hearts, possibly via a regulatory effect of LXRα on *Pdk4*, which negatively regulates pyruvate dehydrogenase complex (PDC) activity. This is in contrast to previous reports showing that, in the protection against cardiac stress, GLUT1 overexpression corrected insufficient glucose utilization and oxidation caused by PPARα deficiency in mice (Luptak *et al*, 2005), and preserved mitochondrial energetic status (Liao *et al*, 2002). That glucose oxidative capacity is not increased in LXRα-Tg hearts may be due to the fact that mitochondrial oxidation rates are indeed normal and not compromised, and since myocardial contractility is unimpaired is evidence that ATP supply is sufficient to fuel contraction. Consequently, excess glucose uptake is neither stored nor oxidized, but is instead diverted into other glycolytic functions due to a modulatory effect of *Pdk4*.

Currently, the role of glucose signaling independent of its energy-providing effects is largely unaddressed in the hypertrophic and failing heart, but has been implicated to play an important role in myocyte function and survival (Kolwicz *et al*, 2013). Moreover, the fate of glucose is of interest given that increased glucose uptake and glycolysis in cardiac hypertrophy do not always result in concomitant increases in glucose oxidation (Allard *et al*, 1994; Wambolt *et al*, 1999; Doenst *et al*, 2013). Our data indicate that, by enhancing glucose flux, cardiac LXRα activates an ancillary pathway of glycolysis, the HBP, increasing levels of the posttranslational modifier, O-GlcNAc. Further, we establish that this pathway induces transcription of natriuretic peptides via glycosylation of GATA4 and Mef2c, transcriptional activators of ANP and BNP (Morin *et al*, 2000). It is interesting that LXRα-Tg hearts exhibit increased basal ANP and BNP mRNA levels without inducing the complete fetal gene response or displaying signs of cardiac dysfunction normally associated with their induction, suggesting that the expression of individual fetal genes is indeed regulated by distinct signal mechanisms. Nevertheless, the cardioprotective effects of natriuretic peptide signaling are well established (Nishikimi *et al*, 2006), and murine models with ablated natriuretic peptide signaling show increased propensity for cardiac hypertrophy and myocardial fibrosis (Tamura *et al*, 2000; Holtwick *et al*, 2003; Wang *et al*, 2003). Therefore, the anti-hypertrophic and anti-fibrotic potential of local ANP and BNP signaling may largely contribute to the protective phenotype observed in LXRα-Tg mice, an adaptive response mediated via transcriptional control of glucose-O-GlcNAc-dependent signaling by cardiac LXRα. Future studies aimed at elucidating additional O-GlcNAc targets should provide further insight into the link between myocyte metabolism and survival in the diseased heart.

In clinical cardiology, progressive cardiac remodeling often transitions into overt symptomatic heart failure, and although several effective treatments have been developed to prevent this transition, there remains a high residual risk. Alterations in myocardial substrate metabolism contribute to this progression; however, no metabolic modulators are part of the guideline-based therapy for heart failure. Promising pharmacological agents such as trimetazidine and perhexiline inhibit FA oxidation and indirectly cause a reciprocal shift to glucose utilization, yet drugs for direct glucose enhancement are not available. Our data indicate that targeting LXRα as a metabolic intervention to increase glucose metabolism profoundly influenced cardiac hypertrophy and remodeling, independent of hemodynamic or neurohormonal effects.

In conclusion, this study demonstrates that LXRα confers heart-specific protective effects in the attenuation of pathological LV hypertrophy and preservation of cardiac function. We identify LXRα as an important cardiac transcriptional regulator that further promotes the adaptive capacity for glucose uptake and utilization in cardiac hypertrophy. Furthermore, this study highlights the under-recognized potential for non-energy-dependent pathways of glycolysis such as the HBP in promoting cytoprotection. New generation LXR agonists with less lipogenic profiles are currently being developed, and we postulate that such agonists may be useful modulators of myocyte metabolism in the prevention of pathological cardiac remodeling and heart failure.

## Materials and Methods

For more detailed Methods, see the Supplementary Information.

### Animal models

The murine NR1H3 gene (GeneBank: NM_013839) was obtained from German Science Centre for Genomic Research (RZPD; clone # IRAVp968B0923D). This PCR product was amplified by polymerase chain reaction (PCR) and cloned into a previously described vector containing the cardiac-specific αMHC promoter (Gulick *et al*, 1991). Transgenic founders were obtained by pronuclear injection of the αMHC-LXRα construct into FVB oocytes. Transgene identification was performed by a PCR-based method using the following primers: sense 5′-CGGCACTCTTAGCAAACCTC-3′, antisense 5′-TGCTGACTCCAACCCTATCC-3′. Mice were backcrossed for at least three generations into the C57BL/6 (#000664, The Jackson Laboratory) genetic background. αMHC-LXRα (LXRα-Tg) mice were generated by the UMCG mouse clinic in collaboration with the Mayo Clinic (Rochester, NY, USA). For all experiments, non-transgenic littermates (Wt) served as controls.

Homozygous LXRα knockout mice (LXRα^−/−^; gift from Dr. Gustafsson) (Alberti *et al*, 2001) and matching C57BL/6BomTac wild-type (WT) mice were obtained from Taconic, Denmark.

### Experimental protocol

All experimental protocols were approved by the Institutional Animal Care and Use Committee at the University of Groningen. Male mice (8–10 weeks) were subjected to an infusion of angiotensin II (Ang II) (1.0 mg/kg/day) for 10 days, or pressure overload by transverse aortic constriction (TAC) for either 1 or 5 weeks. In subsequent studies, a subset of mice underwent sham/TAC for 5 weeks for further assessment of myocardial 2-deoxy-2-(^18^F)fluoro-D-glucose (FDG)-glucose uptake with microPET, or mitochondrial oxidative phosphorylation measurements. Cardiac function was determined with echocardiography and invasive hemodynamic monitoring, as previously described (Yu *et al*, 2013). LV tissue samples were used to perform expressional studies, immunohistochemical, and biochemical analyses.

### *In vitro* studies

Neonatal rat ventricular myocytes (NRVMs) were isolated from 1- to 3-day-old Sprague Dawley pups, as described (Lu *et al*, 2012). Recombinant adenovirus containing murine LXRα (Ad-LXRα) or silenced LXRα (si-LXRα) was used to infect NRVMs, and cells were treated with phenylephrine (PE) or 6-diazo-5-oxonorleucine (DON) (Sigma). Cellular glucose uptake and protein synthesis were assessed with 2-deoxyglucose (2-DG) and [^3^H]leucine assays, respectively.

### Statistical analysis

All data are presented as means ± standard error of the mean (SEM). Student’s paired 2-tailed *t*-test was used for two group comparisons. One-way ANOVA was performed to analyze differences for multiple-group comparisons, followed by Bonferroni *post hoc* analysis. Kruskal–Wallis test followed by Mann–Whitney *U*-test was used to analyze cell experiments. All results were tested at the *P* < 0.05 level of significance.

**The paper explained**

**Problem**

The heart responds to pathological stress by shifting myocardial substrate metabolism toward a greater reliance on glucose. Liver X receptors (LXRs) are nuclear receptors with described functions in lipid and glucose metabolism. Although it has been suggested that pharmacological LXR activation may protect the heart, lipogenic side effects of current LXR agonists preclude their clinical applicability, while the heart-specific effects of LXRs in cardiac pathophysiology and metabolism remain unknown.

**Results**

Here, we show that LXRα protects the heart from pathological cardiac remodeling. Using a transgenic approach to selectively overexpress LXRα in murine hearts, we demonstrate that the cardioprotective effects of LXRα are indeed heart specific in the attenuation of cardiac hypertrophy and myocardial fibrosis. LXRα overexpression markedly improved cardiac function, as assessed by echocardiography and invasive hemodynamics. Gene profiling revealed that LXRα transcriptionally reprograms metabolic pathways by upregulating a set of genes involved in glucose metabolism. This was functionally confirmed in isolated cardiomyocytes and *in vivo* with FDG-microPET scanning as LXRα overexpression markedly enhanced the capacity for glucose uptake in response to hypertrophic stress. Conversely, LXRα-deficient mice displayed an impaired adaptive capacity in augmenting glucose uptake. Further, cardiac LXRα overexpression promoted energy-independent utilization of glucose by activating the hexosamine biosynthesis pathway, resulting in downstream O-GlcNAc modification of transcription factors inducing natriuretic peptide expression, a putative end effector of LXRα-mediated anti-hypertrophic effects in the heart.

**Impact**

We identified LXRα to be a key cardiac transcriptional regulator that mediates an adaptive metabolic response to pathological cardiac stress. Targeting of LXRs as a metabolic intervention in cardiac hypertrophy and heart failure may therefore represent a promising therapeutic approach.

## Supporting Information

**Source Data for Supplementary Figure S9**

**Review Process File**
