# Supplementary material for: Cardiac LXRα protects against pathological cardiac hypertrophy and dysfunction by enhancing glucose uptake and utilization
Source: EMBO Mol Med. 2015 Jul 14;7(9):1229–43. doi: 10.15252/emmm.201404669 (PMC4568954; doi:10.15252/emmm.201404669)
Supplement: Supplementary file 1 [file emmm0007-1229-sd1.pdf]

SUPPLEMENTAL MATERIAL

Table of Contents

**Supplemental Methods**	**2**
**Supplemental References**	**10**
**Supplemental Figures**	**11**
**Supplemental Tables**	**20**

Supplemental Methods:

Experimental protocol

All experimental protocols were performed in accordance with the *Guide for the Care and Use of Laboratory Animals* (NIH Publication No. 86-23) and with approval from the Institutional Animal Care and Use Committee at the University of Groningen (Groningen, the Netherlands). Male mice (aged 8-10 weeks) were housed on a 12hr:12hr light:dark cycle in a temperature-controlled environment with *ad libitum* access to water and chow. Mice were subjected to an infusion of angiotensin II (Ang II) via osmotic mini pump for 10 days, or pressure overload by transverse aortic constriction (TAC) for either 1 or 5 weeks. In subsequent studies, a subset of mice underwent sham/TAC for 5 weeks for further assessment of myocardial FDG-glucose uptake with microPET or mitochondrial oxidative phosphorylation (oxphos) measurements. For all experiments, non-transgenic littermates (Wt) served as controls. LXRα-null mice (LXRα$^{-/-}$; provided by Dr. Gustafsson) (Alberti et al. 2001) and matching C57BL/6BomTac wild-type (WT) mice were obtained from Taconic, Denmark. Cardiac function was determined with echocardiography and invasive hemodynamic monitoring. Left ventricular (LV) tissue samples were used to perform expressional studies, immunohistochemical, and biochemical analyses.

Transverse aortic constriction

Chronic pressure overload induced via TAC is a well established model (Rockman et al. 1991). Mice were anesthetized with 2% isoflurane/oxygen, intubated, and mechanically ventilated (MiniVent, Harvard Apparatus, Holliston, MA, USA). Thereafter, they were placed supine on a heated pad and a 0.5-1.0 cm skin incision was made to the chest. An additional small incision was made to access the aortic arch between the second intercostal space. The arch was constricted between the brachiocephalic and left carotid arteries with a 7-0 silk suture tied around a blunt 27-gauge needle, creating a reproducible stenosis. After ligation, the needle was immediately removed and the incised skin closed. Carprofen (5.0 mg/kg) was administered subcutaneously, perioperatively, to relieve pain. Sham procedures were identical except the aortic arch was not ligated.

Subcutaneous Ang II infusion

Angiotensin II (1.0 mg/kg/day; dissolved in 0.9% NaCl) was dispensed into osmotic minipumps (Alzet 2004, Palo Alto, CA, USA) according to manufacturer's instructions. Mice were anesthetized with 2% isoflurane/oxygen and a single dose of 3.0 mg/kg flunixin-meglumin was given subcutaneously to alleviate wound pain. A small incision was made at the right flank wherein a subcutaneous pocket was prepared for pump insertion. Control groups received 0.9% NaCl in their pumps.

Echocardiography

In vivo cardiac dimensional and functional parameters were assessed with M-mode and 2D transthoracic echocardiography (Vivid 7 equipped with 14-MHz linear array transducer; GE Healthcare, Chalfont St. Giles, UK) 2-3 days prior to sacrifice. Mice were anesthetized with 2% isoflurane/oxygen, placed on a heating pad maintained at 37°C, followed by removal of chest hair via application of topical depilation agent. Parasternal short axis views were obtained to ensure M-mode recordings were recorded at LV mid-papillary level. From three cine loops, M-mode tracings were used to measure LV dimensions and fractional shortening. Percent fractional shortening (%FS) was calculated as: [(LVIDd-LVIDs)/LVIDd]*100; LVIDd = LV internal diameter in diastole, LVIDs = LV internal diameter in systole. In apical 4-chamber view, pulsed wave Doppler with sample volume placed at mitral valve leaflet separation was used to record mitral flow gradients and E and A filling velocities.

Invasive hemodynamic measurements

In situ hemodynamics were analyzed by aortic and LV catheterization under anesthesia with 2% isoflurane prior to sacrifice. The right carotid artery was isolated, punctured, and an indwelling micromanometer-tipped pressure catheter (1.4 F; Millar Instruments, Houston, TX, USA) was inserted. After three minutes of stabilization, arterial pressures were recorded. The catheter was then advanced into the LV to record intracardiac pressures. Heart rate (HR), aortic pressures, LV end-systolic (LVESP) and end-diastolic (LVEDP) pressures, and maximal and minimal first derivatives of force (dP/dt_{max} and dP/dt_{min}) were recorded. The catheter was then removed and the carotid artery ligated. Remaining under anesthesia, blood was collected via heart puncture. The thoracic cavity was then dissected and hearts were flushed with 10 ml phosphate-buffered saline to wash out red blood cells, then quickly excised and weighed. The remaining ventricle was portioned for either immunohistochemistry or frozen in liquid nitrogen and stored at -80°C until further processing for RNA, protein, and biochemical analyses. In a subsequent TAC cohort (n=26), 50-60 mg of fresh tissue was procured and stored in ice-cold preparation medium and kept on ice for oxphos measurements.

Histological analysis and microscopy

Mid-ventricular transverse sections were either post-fixed in 4% paraformaldehyde for paraffin embedding, or cryopreserved (Tissue-Tek, Sakura Finetek). Paraffin-embedded mid-ventricular tissue were sliced into 4 µm sections and stained with the following: Massons trichrome for detection of collagen, FITC-labeled wheat germ agglutinin (WGA) to quantify myocyte cross-sectional area. Whole stained sections were scanned (Nanozoomer 2.0-HT, Hamamatsu, Japan), and quantification of % fibrosis

of entire section was calculated at 20X magnification (ScanScope, Aperio Technologies, Vista, CA, USA). Cardiomyocyte cross-sectional area was measured and quantified at 20X magnification (ImageJ, NIH, Bethesda, MD, USA). To detect neutral lipid, 4 µm frozen LV sections were stained with hematoxylin-eosin and Oil red O (Sigma Aldrich).

Microarray analysis

Total RNA was extracted from LV tissue using TRIzol reagent (Invitrogen, Carlsbad, CA, USA), and 2 µg of purified total RNA was used to perform whole-genome expression profiling (n=3 per group). RNA quality was checked using the Agilent 2100 Bioanalyzer™. Samples were exponentially amplified from a starting amount of 50 ng to a final amount of one µg purified biotin-labeled cRNA using the Illumina® TotalPrep™-96 RNA Amplification Kit (Ambion, Inc., Austin, TX, USA). This final cRNA was evaluated and the quality, concentration, and size of the reaction productions were measured using the Agilent RNA 6000 Nano Kit (Agilent).

Illumina.SingleColor.MouseRef-8_V2.0 beadchips were used for microarray analysis. Chips were scanned using the BeadXpress Reader™ (Illumina). Beadstudio™ Illumina was used to import the raw data and remove any background noise. Data were converted to standard format and exported for use in Agilent Genespring GX™ (version 12.0, Agilent) with which quantile normalization of each individual well was performed. GeneTrail (http://genetrail.bioinf.uni-sb.de/) was used to cluster differentially expressed genes into highly enriched functional categories according to KEGG PATHWAY analysis (http://www.genome.jp/kegg/pathway.html).The microarray data from this publication have been submitted to the GEO database (http://www.ncbi.nlm.nih.gov/geo/) and assigned accession number GSE69355.

RNA isolation and quantitative real-time PCR

Total RNA was extracted from tissue using TRIzol reagent (Invitrogen, Carlsbad, CA, USA), and from cells using the Nucleospin RNA II kit (Macherey-Nagel, Duren, Germany). From total RNA, 0.5 µg was reverse transcribed to cDNA using RNeasy Mini kit (Qiagen Inc, Valencia, CA, USA). The resulting cDNAs were subjected to quantitative real-time PCR using C1000 Thermal Cycler CFX384 Real-Time PCR Detection System (Bio-Rad Laboratories, Veenendaal, The Netherlands). Quantification of mRNA levels were performed (Bio-Rad CFX Manager 2.0), and transcript measurements were normalized to the invariant transcript, *36b4*. Primer sequences used for quantitative PCR analyses are listed in Supplemental Tables 5 and 6.

Western blotting

Frozen LV tissue was homogenized in ice-cold lysis buffer (50 mM Tris pH 8.0, 1% NP40, 0.5% deoxycholate, 0.1% SDS, 150 mM NaCl, 1 mM PMSF, 15 mM Na Vanadate) supplemented with protease and phosphatase inhibitor cocktails (Sigma). Cells were lysed in ice-cold lysis buffer (50 mM Tris pH 8.0, 1% NP40, 0.5% deoxycholate, 0.1% SDS, 150 mM NaCl, 1 mM PMSF, 2 mM EDTA) supplemented with protease inhibitor and 100 µM *O*-(2-acetamido-2-deoxy-D-glucopyranosylidenamino) *N*-phenylcarbamate (PUGNAc) to prevent removal of O-GlcNAc from cellular proteins. This preparation was also used for O-GlcNAc protein determination in tissue lysates. Protein concentrations were measured using Bio-Rad DC Protein Assay (Bio-Rad) for tissue, and Pierce BCA Protein Assay Kit (Thermo Scientific) for cells. Protein lysates (20-30 µg) were resolved on 8-15% SDS-PAGE gels, and separated proteins were transferred onto 0.2 µm nitrocellulose membranes (Bio-Rad). Immunoblotting was performed using primary and secondary antibodies from the following commercial suppliers: anti-human LXRα (2ZPPZ0412H, R&D Systems, Perseus Proteomics); AMPKα (#2532), phospho-AMPKα (Thr172) (#2535), GLUT4 (#2213), hexokinase II (#2867) (Cell Signaling); anti-glucose transporter GLUT1 (ab40084, Abcam); CD36 (gift from Dr. Koonen, Groningen, The Netherlands); Bax (B-9) (sc-7480), Bcl-2 (C-2) (sc-7382) (Santa Cruz); O-GlcNAc CTD110.6 (MMS-248R, Covance) supplemented where indicated with *N*-acetylglucosamine (GlcNAc) (Sigma) to demonstrate antibody specifity (Suppl. Fig. 9B); glyceraldehyde-3-phosphate dehydrogenase (10R-G109A, Fitzgerald, USA); rabbit anti-mouse immunoglobulins/HRP (P0260, Dako, Denmark); goat anti-rabbit immunoglobulins/HRP (P0448, Dako, Denmark). Signals were detected by ECL (PerkinElmer, Waltham, MA, USA), and densitometry was quantified with ImageQuant LAS 4000 (GE Healthcare Europe GmbH, Diegem, Belgium). Fold changes were calculated and are shown.

Biochemical assays

Myocardial lipids were extracted from 40-60 mg of LV tissue according to Bligh & Dyer methods (BLIGH and DYER. 1959). Commercially available kits were used to measure the following: triglycerides (Roche Diagnostics, Mannheim, Germany), total cholesterol and non-esterified fatty acids (NEFA) (DiaSys, Holzheim, Germany). Phospholipids were quantified as detailed (Böttcher et al. 1961). For measurement of myocardial glycogen, 20 mg of LV tissue was boiled in 2N HCl at 99°C in a heat block for 2 hrs with occasional vortex. Tubes were re-weighed and reconstituted with dH_2O to original volume. Samples were neutralized with 2N NaOH. Glycogen content was measured using EnzyChrom Glycogen Assay Kit (BioAssay Systems, Hayward, CA, USA).

Myocardial FDG-glucose uptake

Mice were anesthetized by inhalation of medical air and 2% isoflurane. Immediately prior to scan, blood glucose was sampled via tail vein bleeding (Accu-Chek Aviva; Roche Diagnostics, Mannheim, Germany). Small-animal PET was performed on a microPET Focus 220 system (Siemens, USA). Inside the camera, mice were placed on a heated pad and maintained under anesthesia (1.5% isolflurane). A 200 µl bolus of ^{18}F-FDG, approximately 5 MBq, was administered via the penile vein. PET emission data were acquired for 30 min followed by a 10 min transmission scan to correct for photon attenuation and scatter. The PET images were reconstructed into a matrix of 512 X 512 pixels and analyzed using Inveon Research Workplace (Siemens, USA). Three consecutive ROIs (region of interest) were selected from the LV myocardium in the frontal and coronal planes. The myocardial ^{18}F-FDG uptake was calculated as a standardized uptake value (SUV): SUV = mean tissue counts (Bq/ml)/[injected dose (Bq)/body weight (g)]. All data were corrected for time of decay ($t_{1/2}$ = ^{18}F 109.8 min) before and after tracer injection.

Mitochondrial function and citrate synthase activity

Mitochondrial oxphos was measured in fresh LV tissue biopsies with pyruvate (5 mM) and parmitoyl-carnitine (2 mM) as substrates, each combined with malate (5 mM) and carnitine (5 mM). For preparation of permeabilized myocardial fibers, approximately 10-40 mg tissue was dissected from LV and transferred to 2 ml ice-cold preparation medium (20 mM imidazole, 20 mM taurine, 0.5 mM dithiothreitol, 7.1 mM $MgCl_2$, 50 mM MES, 5 mM ATP, 15 mM phosphocreatine, 2.62 mM CaK_2EGTA, 7.38 mM K_2EGTA, pH 7.0 adjusted with KOH). After brief manual separation of tissue, muscle fibers were permeabilized in preparation medium supplemented with saponin by gentle agitation for 15 min on ice. Fibers were kept on ice and washed twice for 10 min by agitation in 1.5 ml washing medium (20 mM imidazole, 20 mM taurine, 0.5 mM dithiothreitol, 1.61 mM $MgCl_2$, 100 mM MES, 3 mM KH_2PO4, 2.95 mM CaK_2EGTA, 7.05 mM K_2EGTA, pH 7.1 adjusted with KOH), and immediately used for respirometric measurements. High-resolution respirometry was performed at 37ºC with 2.5-3.5 mg tissue biopsy using OROBOROS Oxygraph-2k (OROBOROS Instruments, Innsbruck, Austria). Oxygen consumption rates were measured in 1.5 ml of MiR05 buffer (0.5 mM EGTA, 3 mM $MgCl_2.6H_2O$, 60 mM K-lactobionate, 20 mM taurine, 10 mM KH_2PO_4, 20 mM HEPES, 110 mM sucrose, 1 g/L BSA) at 37ºC. The O_2 solubility factor of the buffer is 0.920. Datlab Software (OROBOROS Instruments) was used for data acquisition and analysis. Oxygen consumption was recorded under three conditions: basal respiration rates in the presence of substrate alone before the addition of ADP was defined as state 2, maximal ADP-stimulated (4 mM) respiration rates was defined as state 3, and respiration rates in the absence of ADP phosphorylation and measured in the presence of oligomyocin (1 mM), an ATP synthase inhibitor, was termed state 4. Respiratory control ratio was calculated as the ratio of state 3 to state 4.

Oxygen consumption rates were expressed as nmol O_2/min per dry tissue weight (mg), and normalized to citrate synthase activity (Sigma Aldrich).

WGA precipitation of O-GlcNAcylated proteins

Nuclear extracts were prepared from three pooled hearts using the NE-PER Nuclear and Cytoplasmic kit (Thermo Scientific). In brief, left ventricular tissue was minced into small pieces and subjected to homogenization using a Dounce homogenizer. Cytoplasmic extracts were obtained and stored at -20°C, and supernatant containing nuclear extracts was assayed to determine protein concentration (Bio-Rad). Precipitation of O-GlcNAcylated proteins was performed with wheat germ agglutinin (WGA)-conjugated agarose beads, a lectin that binds to GlycNAcylated proteins (Vector Laboratories, Burlingame, CA, USA). The agarose WGA gel was washed twice from the stabilizing sugar with a bead binding buffer (20 mM Tris pH 7.4, 1 mM $CaCl_2$, 1 mM $MgCl_2$). Nuclear proteins (500 µg) were diluted 4X in bead binding buffer containing 1 µM PUGNAc. Of the diluted sample, 950 µl was added to 25 µl of washed agarose WGA solution, followed by overnight incubation at 4°C on a rocking platform. *N*-acetylglucosamine (GlcNAc; 20 mM), a competitor, was added where indicated and served as a control (Sigma). The precipitated agarose WGA-protein complexes were obtained by centrifugation at 10,000 *g* for 1 min, and then washed three times with bead binding buffer. The complexes were then resuspended in protein sample buffer and boiled for 5 min. Samples were loaded on a 8% SDS polyacrylamide gel for electrophoresis, and Western blot was performed to identify eluted proteins using the following antibodies: GATA-4 (sc-25310), Nkx-2.5 (sc-14033) (Santa Cruz Biotechnology, Inc.); MEF2C (#5030) (Cell Signaling).

Neonatal rat ventricular myocyte isolation and culture

Neonatal rat ventricular myocytes (NRVMs) were isolated from 1-3 day old Sprague-Dawley pups as previously described (Lu et al. 2010). Cells were cultured at 37°C in the presence of 5% CO_2 in Dulbecco's Modified Eagle Medium (DMEM) containing 5% fetal calf serum (FCS) and penicillin-streptomycin (100 IU/ml and 100 µg/ml, respectively) (Invitrogen, Breda, The Netherlands). Where indicated, cells were treated for 24 hours with the following in serum-free DMEM: 50 µM phenylephrine (PE), an α1-adrenergic receptor agonist which induces cellular hypertrophy (Lu et al. 2010); 100 µM 6-diazo-5-oxonorleucine (DON) (Sigma), an inhibitor of glutamine:fructose-6-phosphate amidotransferase (GFAT), the first and rate-limiting enzyme in the hexosamine biosynthesis pathway (HBP) (Facundo et al. 2012); 100 µM PUGNAc (Sigma).

Adenoviral infection

Adenoviral constructs and recombinant adenovirus containing murine LXRα (Ad-LXRα) and LXRα-specific siRNA (si-LXRα) were produced as described previously (Lu et al. 2010). Adenoviral sequences used for cloning are listed in Supplemental Table 7. A GFP-expressing virus, GL2 (Ad-cont), was used as a control (Lu et al. 2010). Cells were infected overnight at MOI of 50, unless otherwise indicated. After overnight incubation, cells were washed three times with PBS and culturing was continued in serum-free DMEM medium. Following 24 hours incubation in serum-free DMEM medium, the indicated treatments were administered over a 24 hour period.

***In vitro* glucose uptake assay**

Glucose uptake in NRVMs was assayed using Glucose Uptake Colorimetric Assay Kit (Abcam). Briefly, 40×10^3 NRVMs were seeded per well in a 96-well plate, and cells were infected and treated as described above. Prior to start of assay, cells were washed three times with PBS and incubated in 100 µl Krebs-Ringer-Phosphate-Hepes (KRPH) solution for 40 min. Insulin was administered as a positive control, and was added 20 min into incubation time for an additional 20 min. Thereafter, 10 µl of 10 mM 2-deoxyglucose (2-DG) was added to each well. To assess specificity, 20-fold excess glucose (20 mM final concentration) was added to a control well (Suppl. Fig. 9A). After 20 min incubation, cells were washed three times with PBS and lysed with extraction buffer. Sample preparation was performed in accordance with manufacturer's instructions, and absorbance was measured at 412 nm using a spectrophotometer (Benchmark Plus Microplate Reader, Bio-Rad). Fold changes, as compared to control cells, were calculated and are shown.

Immunofluorescence staining

Isolated cardiomyocytes were cultured in 12-well plates on 18 mm coverslips coated with laminin. Cells were fixed in 4% paraformaldehyde for 10 min, then permeabilized with ice-cold 0.3% Triton X100 for 5 min. Blocking was performed in 3% bovine serum albumin (BSA) 0.1% PBS/Tween solution containing 2% goat serum for 1 hour, followed by incubation for 1 hour with a monoclonal anti-human LXRα antibody (2ZPPZ0412H, R&D Systems, Perseus Proteomics). After washing, cells were further incubated with goat anti-mouse IgG-FITC secondary antibody (sc-2010, Santa Cruz, Heidelberg, Germany) and fluorescent phalloidin-rhodamine (Invitrogen, Breda, The Netherlands) for detection of F-actin. Coverslips were mounted using Vectashield mounting medium with 4',6-diamidino-2-phenylindole (DAPI) (Vector Laboratories, Burlingame, CA, USA), and imaged with a confocal microscope (Leica Microsystems, Wetzlar, Germany). Cell size was determined as previously described (Cannon et al. 2015).

Protein synthesis assay

NRVMs were transfected with the following adenoviruses, Ad-LXR, si-LXRα, or Ad-cont, for 24 hours, then cultured in serum-free DMEM medium for 24 hours. [^{3}H]Leucine was administered to cells for a further 24 hours, and determination of protein incorporation is as previously described (Lu et al. 2010).

Statistical analysis

All data are presented as means ± standard error of the mean (SEM). Student's paired 2-tailed t-test was used for two group comparisons. One-way ANOVA was performed to analyze differences for multiple-group comparisons, followed by Bonferroni post hoc analysis to assess statistical significance. Kruskall-Wallis test followed by Mann-Whitney *U* test was used to analyze cell experiments (n=4-5). All results were tested at the $P<0.05$ level of significance. Statistical analyses were performed using IBM SPSS Statistics 22 software (Chicago, IL, USA).

Supplemental References:

Alberti S, Schuster G, Parini P, Feltkamp D, Diczfalusy U, Rudling M, Angelin B, Bjorkhem I, Pettersson S, Gustafsson JA (2001) Hepatic cholesterol metabolism and resistance to dietary cholesterol in LXRbeta-deficient mice. J Clin Invest 107: 565-573.

BLIGH EG, DYER WJ (1959) A rapid method of total lipid extraction and purification. Can J Biochem Physiol 37: 911-917.

Böttcher CJF, Van gent CM, Pries C (1961) A rapid and sensitive sub-micro phosphorus determination. Analytica Chimica Acta 24: 203-204.

Cannon MV, Yu H, Candido WM, Dokter MM, Lindstedt EL, Sillje HH, van Gilst WH, de Boer RA (2015) The liver X receptor agonist AZ876 protects against pathological cardiac hypertrophy and fibrosis without lipogenic side effects. Eur J Heart Fail .

Facundo HT, Brainard RE, Watson LJ, Ngoh GA, Hamid T, Prabhu SD, Jones SP (2012) O-GlcNAc signaling is essential for NFAT-mediated transcriptional reprogramming during cardiomyocyte hypertrophy. Am J Physiol Heart Circ Physiol 302: H2122-30.

Lu B, Mahmud H, Maass AH, Yu B, van Gilst WH, de Boer RA, Sillje HH (2010) The Plk1 inhibitor BI 2536 temporarily arrests primary cardiac fibroblasts in mitosis and generates aneuploidy in vitro. PLoS One 5: e12963.

Rockman HA, Ross RS, Harris AN, Knowlton KU, Steinhelper ME, Field LJ, Ross J,Jr, Chien KR (1991) Segregation of atrial-specific and inducible expression of an atrial natriuretic factor transgene in an in vivo murine model of cardiac hypertrophy. Proc Natl Acad Sci U S A 88: 8277-8281.

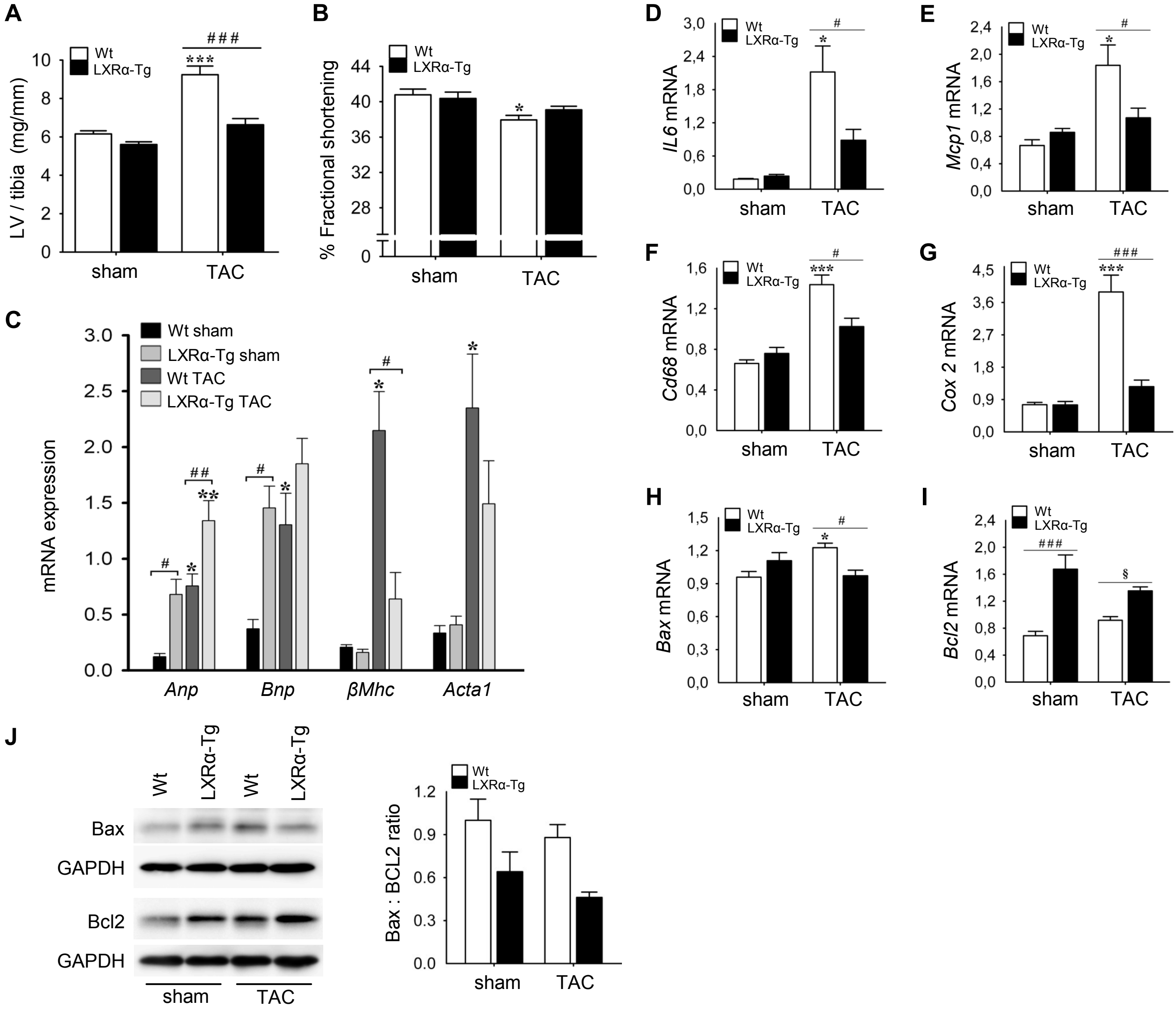

Supplemental Figure 1

Response of cardiac LXRα overexpression in the early phase of pressure overload-induced cardiac hypertrophy.

LXRα-Tg and Wt mice were subjected to 1 week of transverse aortic constriction (TAC).

(**A**) Left ventricular weight to tibia length ratios (LV/tibia), n=8/group, except n=7 Wt sham. ***P=0.00001 versus Wt sham, $^{\#\#\#}$P<0.00001.

(**B**) Functional determination of percent fractional shortening with echocardiography, n=8/group, except n=7 Wt sham. *P=0.01 versus Wt sham.

(**C-I**) Assessment of molecular determinants of (**C**) hypertrophy, (**D-G**) inflammation, and (**H-I**) apoptosis in the LV; RT-PCR analysis of mRNA levels were normalized to *36b4*; n=8/group, except n=7 Wt sham.

(**C**) *Anp*: *P=0.01 versus Wt sham, $^{\#}$P=0.04, **P=0.007 versus LXRα-Tg sham, $^{\#\#}$P=0.02; *Bnp*: *P=0.03 versus Wt sham, $^{\#}$P=0.01; *βMhc*: *P=0.00001 versus Wt sham, $^{\#}$P=0.0002; *Acta1*: *P=0.001 versus Wt sham. (**D-I**) *IL6*: *P=0.0001 versus Wt sham, $^{\#}$P=0.01; *Mcp1*: *P=0.001 versus Wt sham, $^{\#}$P=0.03; *Cd68*: ***P<0.00001 versus Wt sham, $^{\#}$P=0.002; *Cox2*: ***P<0.00001 versus Wt sham, $^{\#\#\#}$P<0.00001; *Bax*: *P=0.02 versus Wt sham, $^{\#}$P=0.02; *Bcl2*: $^{\#\#\#}$P=0.00004, §P=0.09.

(**J**) Western blot assessment of apoptosis markers after 1 week pressure overload, Bax and Bcl2, quantified as ratio; protein normalized to GAPDH, n=6/group.

Data information: data are means ± SEM; one-way ANOVA with Bonferroni's multiple comparison test was used to compare groups.

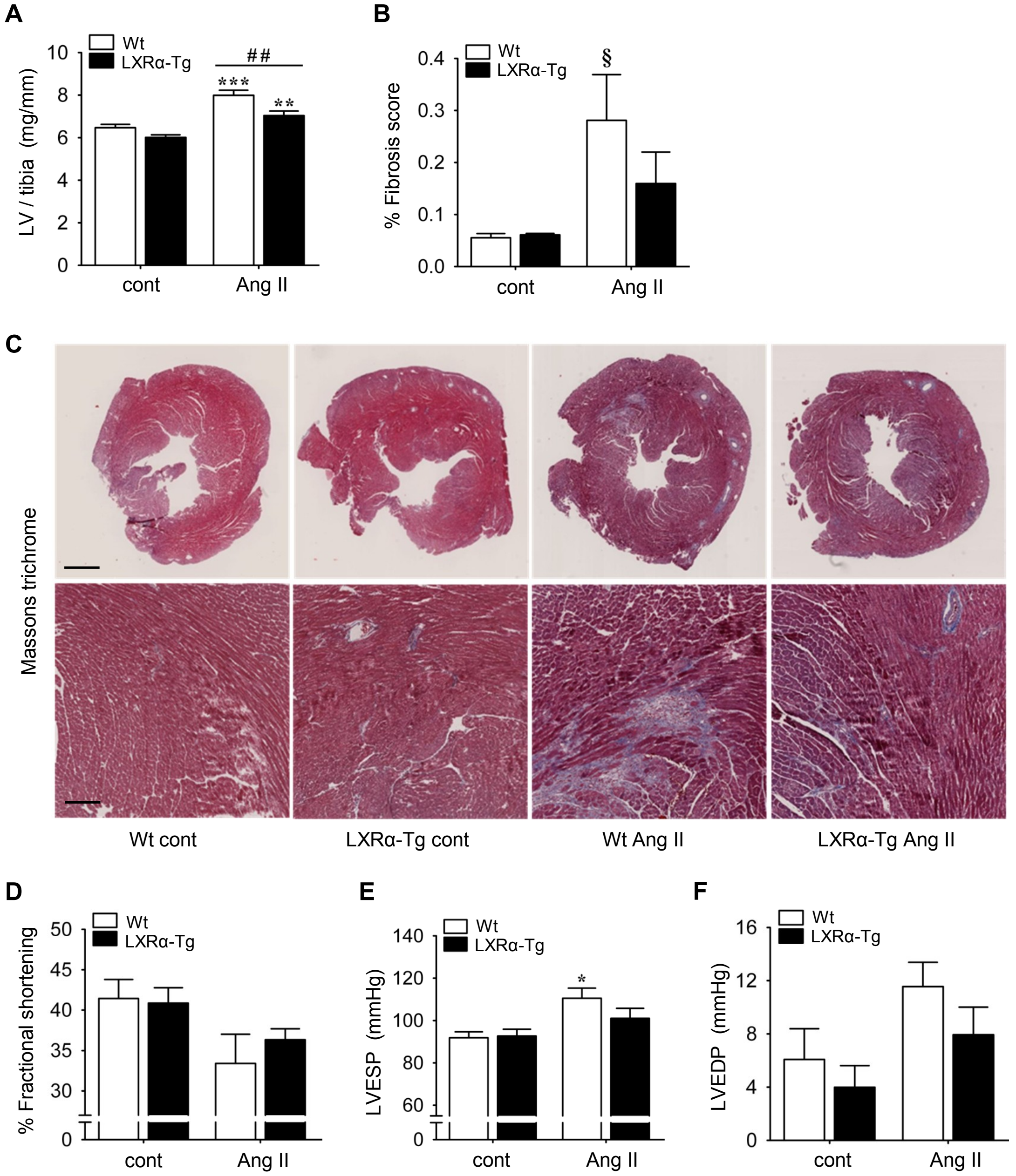

Supplemental Figure 2

Cardiac LXRα protects against angiotensin (Ang) II stimulation.

Cardiac morphometry and *in vivo* functional analysis following 10 days of Ang II (1 mg/kg/day) infusion. (**A**) Left ventricular weight to tibia length ratios (LV/tibia) of Wt and LXRα-Tg mice receiving saline (cont) or Ang II infusion via osmotic minipumps; n=6-8/cont group, n=12-14/ Ang II group. **P=0.01, ***P=0.0005 versus respective control, $^{\#\#}$P=0.007.

(**B-C**) Representative LV histological sections stained with Massons trichrome (bars = 1 mm, 100 μm) for quantification of percent fibrosis of whole heart; n=7 Wt cont, n=6 LXRα-Tg cont, n=8/Ang II group. §P=0.07 versus Wt cont.

(**D**) Echocardiographical assessment of percent fractional shortening; n=6 Wt cont, n=5 LXRα-Tg cont, n=4/Ang II group.

(**E-F**) Hemodynamic monitoring was performed *in situ* to record: (**E**) LV end-systolic pressure (LVESP), and (**F**) LV end-diastolic pressure (LVEDP); n=5-9/group. *P=0.03 versus Wt cont.

Data information: data are means ± SEM; one-way ANOVA with Bonferroni's multiple comparison test was used to compare groups.

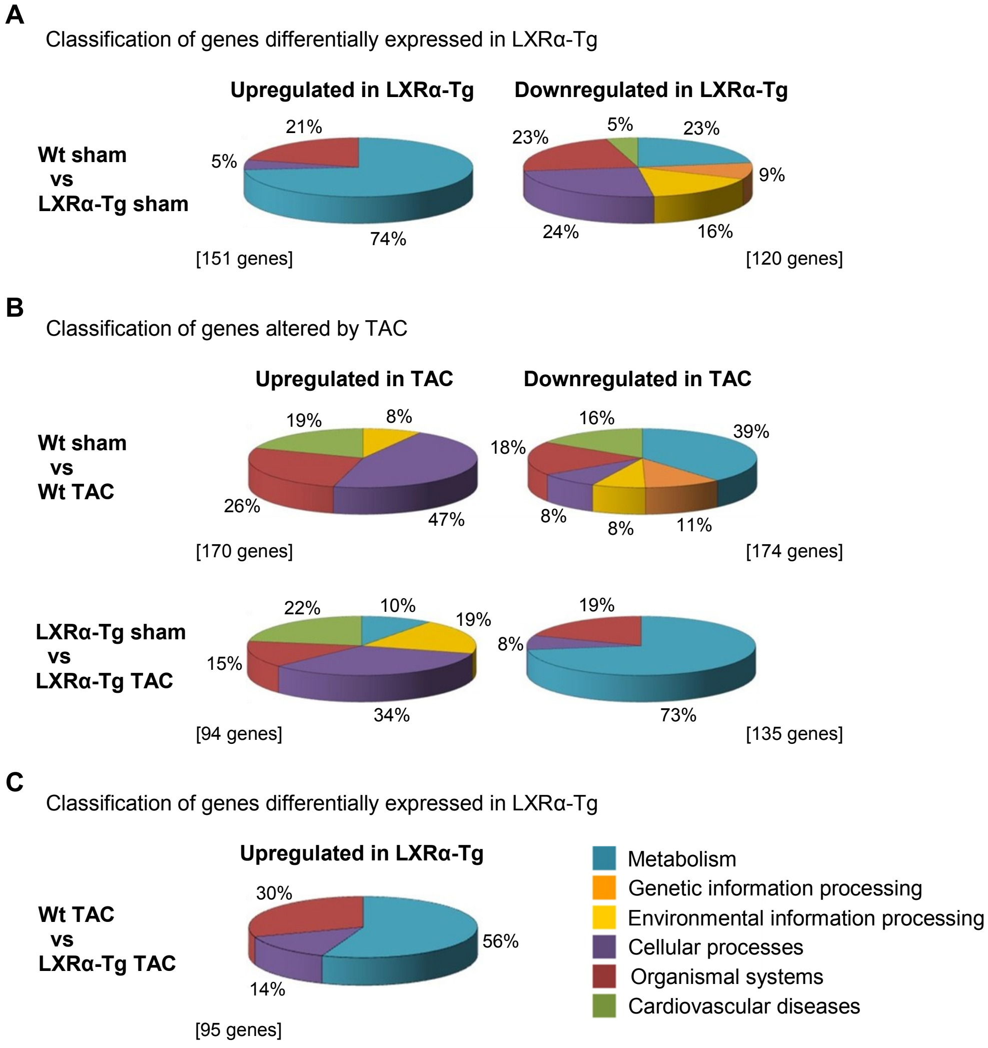

Supplemental Figure 3

Microarray analysis of left ventricular transcripts shows alterations in metabolic pathways upon myocardial LXRα overexpression.

(**A-C**) Differentially expressed genes (1.2-fold, P<0.05) were classified as either up- or downregulated (n=3/group). Sets of genes were further clustered into functional biological pathways (KEGG PATHWAY analysis, FDR adjustment, P<0.0001), and major categories are shown. Gene numbers denoted are number of functionally annotated genes identified for highly enriched pathways per up or down classification, and their relative distribution within major categories are displayed as segmental percentages.

(**A**) Differentially expressed genes in sham-operated LXRα-Tg versus Wt mice.

(**B**) Genotype-specific assessment of TAC-induced alterations.

(**C**) Comparative analysis of upregulated genes between Wt and LXRα-Tg mice subjected to TAC.

No relevant categories were significantly enriched for downregulated genes.

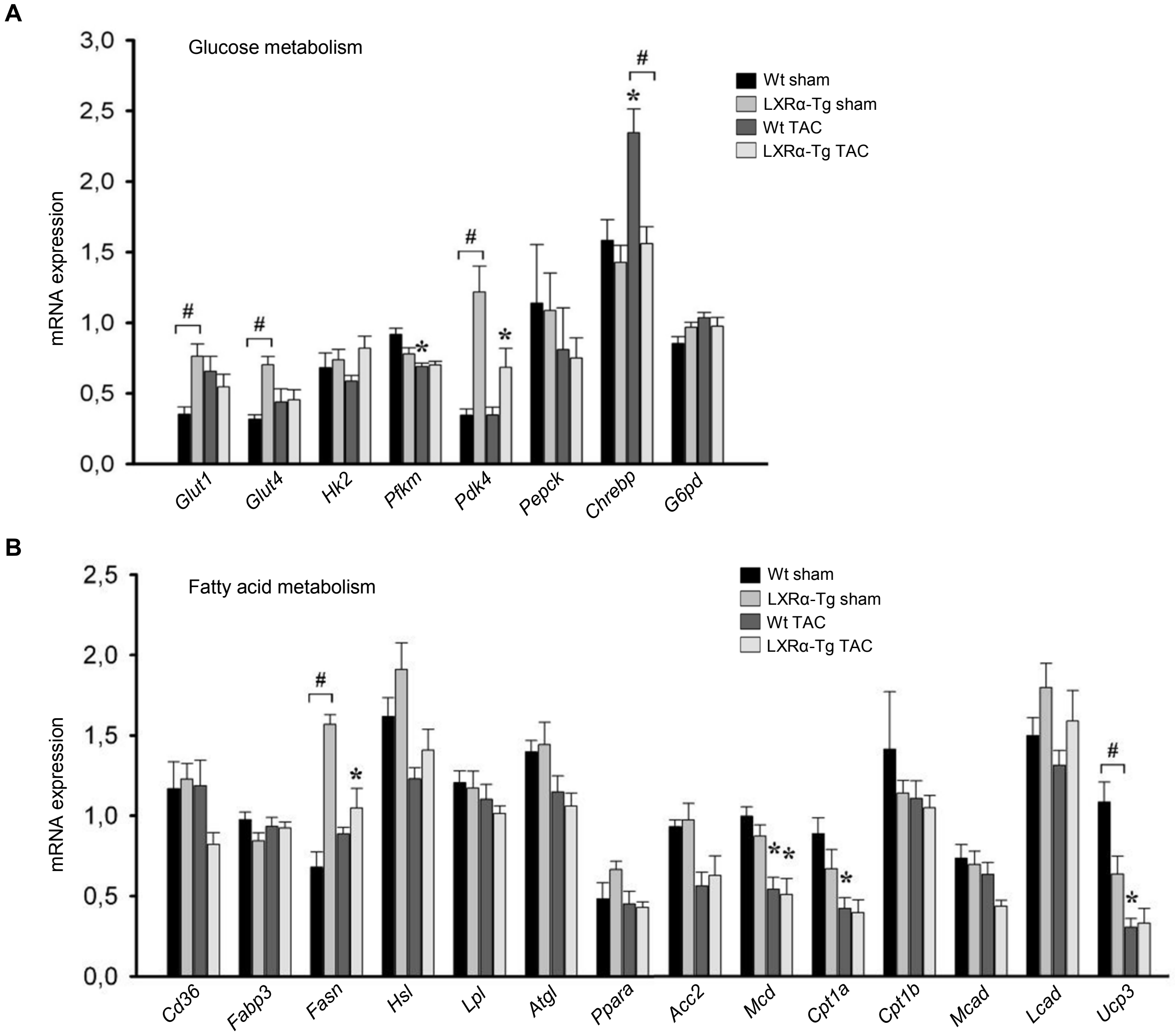

Supplemental Figure 4

Metabolic gene expression in the heart following 5 weeks of chronic pressure overload.

(**A**) Key genes involved in glucose, and (**B**) fatty acid metabolism were determined to assess the impact of cardiac-specific LXRα overexpression at baseline and following hypertrophic perturbation. Quantitative PCR analysis of left ventricular mRNA levels normalized to invariant transcript, *36b4*; n=6-8/group.

(**A**) *Glut1*: $^{\#}$P=0.009; *Glut4*: $^{\#}$P=0.001; *Pfkm*: *P=0.001 versus Wt sham; *Pdk4*: *P=0.03 versus LXRα-Tg sham, $^{\#}$P=0.0001; *Chrebp*: *P=0.004 versus Wt sham, $^{\#}$P=0.003.

(**B**) *Fasn*: *P=0.001 versus LXRα-Tg sham, $^{\#}$P<0.00001; *Mcd*: *P=0.002 versus Wt sham, *P=0.01 versus LXRα-Tg sham; *Cpt1a*: *P=0.02 versus Wt sham; *Ucp3*: *P=0.00008 versus Wt sham, $^{\#}$P=0.02.

Data information: data are means ± SEM; one-way ANOVA with Bonferroni's multiple comparison test was used to compare groups.

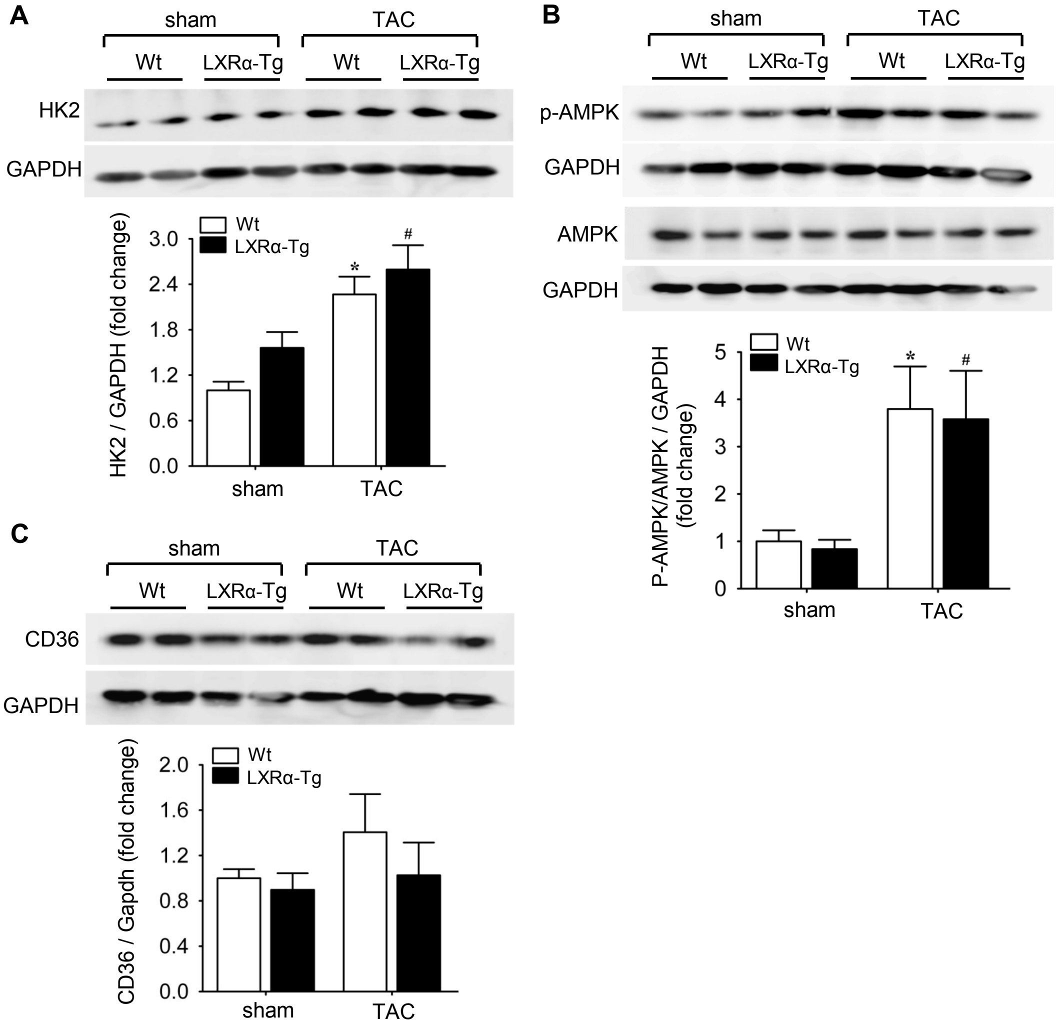

Supplemental Figure 5

Assessment of key metabolic proteins in substrate utilization following 5 weeks of TAC.

Protein was measured in the left ventricle from Western blot and expressed as fold change for the following: (**A**) hexokinase 2 (HK2), (**B**) phosphorylated AMPK to total AMPK, and (**C**) CD36. GAPDH is shown as control and for normalization in quantification; n=6-8/group. HK2: *P=0.004 versus Wt sham, #P=0.04 versus LXRα-Tg sham; P-AMPK/AMPK: *P=0.049 versus Wt sham, #P=0.034 versus LXRα-Tg sham.

Data information: data are means ± SEM; one-way ANOVA with Bonferroni's multiple comparison test was used to compare groups.

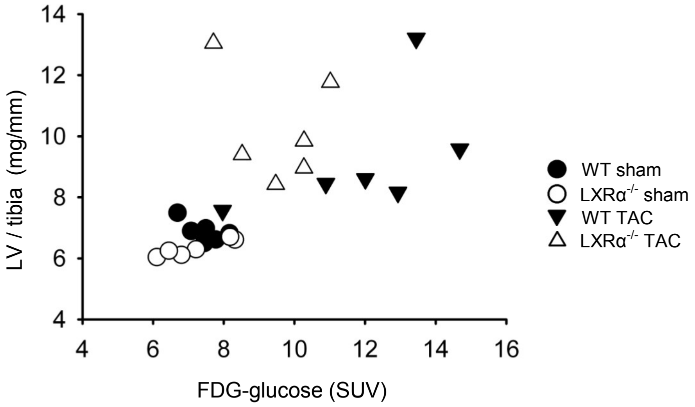

Supplemental Figure 6

LXRα-deficiency impairs augmentations in glucose uptake

Assessment of myocardial ^{18}F-FDG-glucose uptake levels (SUV, standard uptake value) with respect to developed left ventricular hypertrophy (LV/tibia ratio) in LXRα$^{-/-}$ mice and WT cohorts following 5 weeks of pressure overload. Data information: n=6 per group.

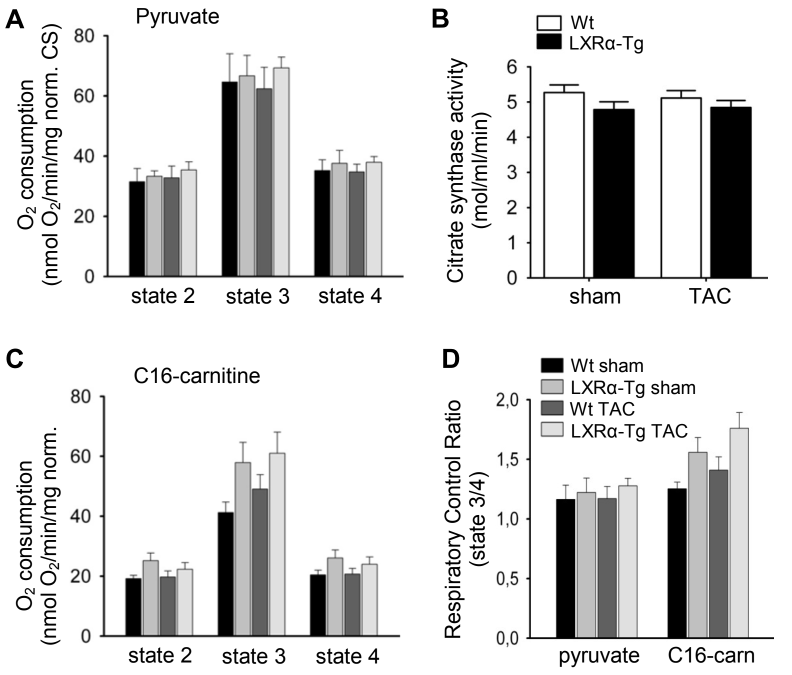

Supplemental Figure 7

Effect of cardiac LXRα overexpression on mitochondrial oxidative capacity.

(**A, C-D**) Mitochondrial oxygen consumption rates were measured in permeabilized LV muscle fibers from sham- and TAC-operated mice in the presence of (**A**) pyruvate, and (**C**) parmitoyl (C16)-carnitine (FA substrate).

Data were recorded under basal conditions (state 2) in the presence of substrate alone, maximal ADP-stimulated respiration (state 3), and respiration rates with the addition of oligomyocin to inhibit ADP phosphorylation by ATP synthase (state 4); CS, citrate synthase; n=6 per sham group, n=8 Wt TAC, n=7 LXRα-Tg TAC. No significant differences are reported.

(**D**) Respiratory control ratios (RCR) were determined from the ratio of state 3 to 4, indicative of mitochondrial function; n=6 per sham group, n=8 Wt TAC, n=7 LXRα-Tg TAC. No significant differences are reported.

(**B**) Individual oxygen consumption rates were normalized to citrate synthase activity, and group means are displayed; n=6 per sham group, n=8 Wt TAC, n=7 LXRα-Tg TAC. No significant differences are reported.

Data information: data are means ± SEM; one-way ANOVA with Bonferroni's multiple comparison test was used to compare groups.

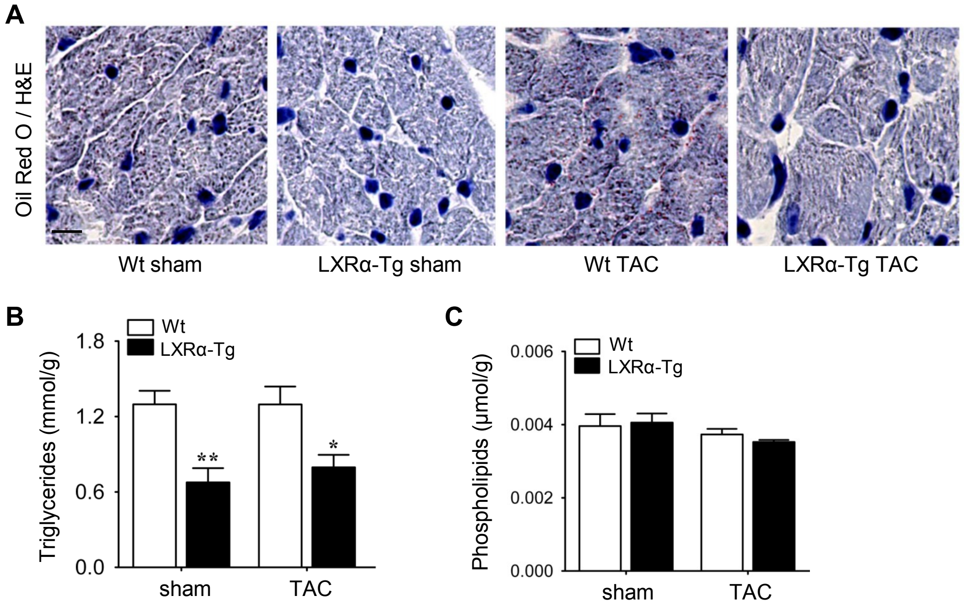

Supplemental Figure 8

Altered lipid homeostasis in murine hearts overexpressing LXRα.

(**A**) Representative LV histological sections of neutral lipid droplets stained with Oil Red O and hematoxylin and eosin (H&E); bar = 10 µm.

Myocardial (**B**) triglyceride, and (**C**) phospholipid content; n=5/group. **P=0.009 versus Wt sham,*P=0.045 versus Wt TAC.

Data information: data are means ± SEM; one-way ANOVA with Bonferroni's multiple comparison test was used to compare groups.

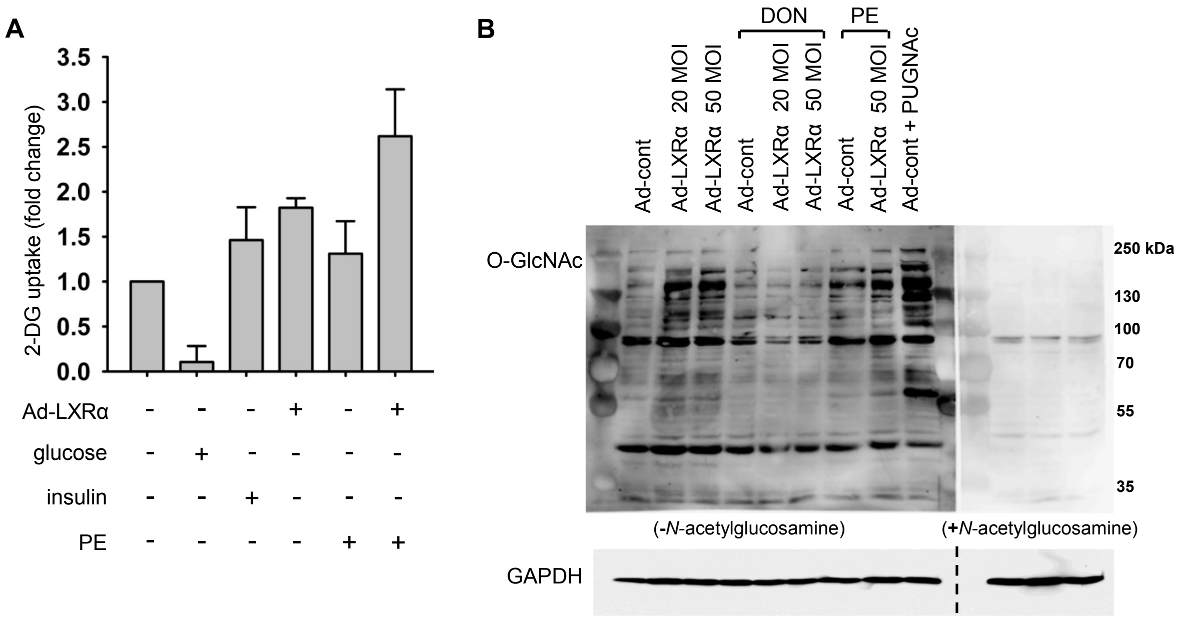

Supplemental Figure 9

Modulation of glucose uptake and O-GlcNAc signaling in cardiomyocytes overexpressing LXRα.

Neonatal rat ventricular myocytes (NRVMs) transfected with Ad-LXRα or GL2 control (Ad-cont) under basal conditions and in response to phenylephrine (PE) treatment for 24 hours.

(**A**) Assessment of 2-deoxyglucose (2-DG) uptake in which excess glucose and insulin served as experimental controls; n=2-4/ group.

(**B**) Additional independent Western blot to Figure 6D indicating Ad-LXRα- and PE-induced increases in global protein O-GlcNAcylation, which was abrogated following inhibition of HBP with DON. PUGNAc was administered to verify increased O-GlcNAc levels, and GAPDH served as loading control.

Dashed line indicates where original membrane was cut prior to separate incubation with *N*-acetylglucosamine, which was used to confirm antibody specificity.

Supplemental Table 1. Baseline Characteristics in Young and Old LXRα-Tg and Wild-type Mice

	**3 months**		**12 months**	
	**Wt**	**LXRα-Tg**	**Wt**	**LXRα-Tg**
Organ weight				
Body weight, g	27.2 ± 0.6	26.2 ± 0.7	40.2 ± 2.0	41.2 ± 2.2
LV weight/tibia, mg/mm	6.3 ± 0.2	5.7 ± 0.1*	8.4 ± 0.4	7.1 ± 0.3*
Kidney weight/tibia, mg/mm	20.7 ± 0.4	20.3 ± 0.2	30.1 ± 0.6	31.1 ± 1.1
Liver weight/tibia, mg/mm	71.5 ± 3.1	71.9 ± 1.7	105.4 ± 5.7	113.7 ± 13.7
Echocardiography				
Interventricular septum, mm				
Diastole	0.70 ± 0.01	0.68 ± 0.01	0.71 ± 0.02	0.75 ± 0.02
Systole	1.60 ± 0.06	1.42 ± 0.04	1.56 ± 0.08	1.47 ± 0.06
LV posterior wall, mm				
Diastole	0.72 ± 0.01	0.70 ± 0.02	0.72 ± 0.02	0.72 ± 0.03
Systole	1.42 ± 0.04	1.43 ± 0.05	1.44 ± 0.05	1.49 ± 0.08
LV internal diameter, mm				
Diastole	3.77 ± 0.03	3.72 ± 0.04	3.91 ± 0.08	3.89 ± 0.11
Systole	2.20 ± 0.03	2.14 ± 0.02	2.22 ± 0.10	2.33 ± 0.06
Fractional shortening, %	41.7 ± 0.7	42.5 ± 0.7	43.4 ± 1.9	39.9 ± 1.2
Hemodynamics				
Heart rate, bpm	454 ± 23	479 ± 15	475 ± 15	501 ± 15
Mean arterial pressure, mmHg	66.2 ± 2.2	66.0 ± 4.2	78.3 ± 2.3	76.9 ± 5.3
LV end-systolic pressure, mmHg	93.8 ± 4.3	87.8 ± 3.4	106.0 ± 2.3	92.4 ± 4.2†
LV end-diastolic pressure, mmHg	5.9 ± 1.7	5.0 ± 1.1	13.5 ± 3.0	11.3 ± 3.3
dP/dt_{max}, mmHg	7999 ± 556	8596 ± 526	7217 ± 414	8154 ± 658
dP/dt_{min}, mmHg	-7847 ± 349	-7601 ± 582	-6111 ± 326	-6951 ± 699
Blood chemistry				
Glucose, mmol/L ‡	12.3 ± 1.1	12.0 ± 0.8		
Triglycerides, mmol/L	0.7 ± 0.1	0.8 ± 0.1	1.7 ± 0.2	1.9 ± 0.3
Cholesterol, mmol/L	2.0 ± 0.1	2.1 ± 0.1	2.3 ± 0.2	2.1 ± 0.1
Non-esterified fatty acids, mmol/L	0.6 ± 0.1	0.6 ± 0.2	0.2 ± 0.03	0.3 ± 0.1

Data are expressed as means ± SEM. * P<0.05, † P<0.001, age-matched Wt versus LXRα-Tg mice. ‡ Not measured in 12-month mice. Wt, 3 months (n=7-15); LXRα-Tg, 3 months (n=6-15); Wt, 12 months (n=8); LXRα-Tg, 12 months (n=7).

Supplemental Table 2. Functional and Biometrical Parameters of LXRα-Tg and Wild-type Mice at Five Weeks Following Transverse Aortic Constriction

	Sham		TAC	
	**Wt**	**LXRα-Tg**	**Wt**	**LXRα-Tg**
Cardiac function	(n=19)	(n=22)	(n=24)	(n=26)
Heart rate, bpm	411 ± 13	428 ± 17	443 ± 10	439 ± 11
Interventricular septum, mm				
Diastole	0.72 ± 0.02	0.71 ± 0.01	1.04 ± 0.02†	0.95 ± 0.02†§
Systole	1.37 ± 0.04	1.27 ± 0.04	1.48 ± 0.03	1.43 ± 0.04*
LV posterior wall, mm				
Diastole	0.75 ± 0.02	0.71 ± 0.01	1.01 ± 0.02†	0.93 ± 0.02†‡
Systole	1.44 ± 0.03	1.41 ± 0.03	1.43 ± 0.05	1.49 ± 0.03
LV internal diameter, mm				
Diastole	4.00 ± 0.06	3.76 ± 0.05	4.00 ± 0.08	3.80 ± 0.07
Systole	2.30 ± 0.09	2.23 ± 0.05	2.72 ± 0.09†	2.50 ± 0.06*
LV mass/BW, mg/g	3.7 ± 0.1	3.2 ± 0.1	6.1 ± 0.2†	4.8 ± 0.2†¶
Fractional shortening, %	40.7 ± 0.9	39.7 ± 0.8	29.8 ± 1.1†	34.1 ± 0.4†§
Ejection fraction, %	77.6 ± 1.0	76.6 ± 0.9	63.4 ± 1.6†	69.5 ± 1.3†§
E/A ratio	1.62 ± 0.06	1.61 ± 0.10	1.60 ± 0.06	1.62 ± 0.08
dP/dt_{max}, mmHg	7761 ± 569	7586 ± 350	7777 ± 327	7514 ± 278
dP/dt_{min}, mmHg	-6889 ± 421	-6823 ± 421	-7732 ± 540	-6564 ± 337
Post mortem	(n=21)	(n=24)	(n=24)	(n=26)
Body weight, g	28.7 ± 0.7	28.6 ± 0.6	28.1 ± 0.3	28.4 ± 0.4
Heart weight/BW, mg/g	5.3 ± 0.1	4.8 ± 0.1§	7.1 ± 0.2†	5.9 ± 0.1†¶
Kidney weight/BW, mg/g [#]	16.7 ± 0.6	16.3 ± 0.4	16.5 ± 0.5	16.4 ± 0.7
Liver weight/BW, mg/g [#]	52.8 ± 2.8	53.8 ± 1.5	56.4 ± 2.0	55.1 ± 1.5

Data are expressed as means ± SEM. * P<0.05, † P<0.001, TAC versus corresponding sham group; ‡ P<0.05, § P<0.01, ¶ P<0.001, Wt vs LXRα-Tg mice. [#] n=7-8/group.

Supplemental Table 3. KEGG PATHWAY Analysis of Differentially Expressed Genes in LXRα-Tg Murine Hearts

Wt sham versus LXRα-Tg sham

Up/Down	Category	Subcategory	P-value	Genes
Up in LXRα-Tg	Metabolism	Drug metabolism – cytochrome P450	$9.6X10^{-9}$	Cyp2c70 Cyp2c37 Cyp2d9 Cyp2c50 Cyp2d10 Cyp2a5 Ugt2b36 Cyp2e1 Ugt1a10 Adh1 Gsta3 Fmo5 Gstt1 Gstm6 Gstz1 Gstp1
Up in LXRα-Tg	Cellular processes	PPAR signaling	$6.5X10^{-7}$	Nr1h3 Slc27a2 Cyp4a12a Apoc3 Apoa1 Apoa2 Cyp8b1 Acaa1b Acox2 Scd1 Hmgcs2 Cyp27a1 Scp2 Acsl5
Up in LXRα-Tg	Metabolism	Metabolic pathways	$1.1X10^{-6}$	Cyp2c70 Cps1 Hpd Cyp2c37 Cyp4a12a Pon1 Cyp2c50 Cyp2a5 Haao Arg1 Ugt2b36 Cyp2e1 Mat1a Uox Cyp8b1 Fbp1 Acaa1b Uroc1 Ass1 Cdo1 Acox2 Ugt1a10 Aldob Gcnt1 Adh1 1190003J15Rik Gck P4ha1 Abat St3gal5 Hmgcs2 Cyp27a1 Sephs2 Pgam1 Scp2 Gamt Coasy Khk Hsd3b7 Gstz1 Ak2 Nme5 Tbxas1 Hsd11b1 Dcxr Acsl5 Gba Pcyt2 Pip5k1b Fdps Gne Hdc Scly Grhpr Aprt Arsb Nt5c3 Inpp5e Gnpda1
Down in LXRα-Tg	Cellular processes	PPAR signaling	$2.6X10^{-5}$	Scd4 Acsl1 Cpt1b Acadm Aqp7 Slc27a1 Cpt2 Pparg Acsl3
Down in LXRα-Tg	Metabolism	Fatty acid metabolism	$3.4X10^{-5}$	Acsl1 Cpt1b Acadm Cpt2 Acaa2 Dci Acsl3

Wt sham versus Wt TAC

Up/Down	Category	Subcategory	P-value	Genes
Up in TAC	Cellular processes	Focal adhesion	$9.5X10^{-8}$	Comp Col1a1 Col6a2 Itga11 Bcl2 Itga5 Col4a4 2900073G15Rik Col6a1 Actn1 Col4a2 Igf1 Pdgfrb Col4a1 Flna Capn2 Actn4 Akt3 Zyx Ilk Itgb1
Up in TAC	Environmental information processes	ECM-receptor interaction	$2.0X10^{-6}$	Comp Col1a1 Cd44 Col6a2 Itga11 Itga5 Col4a4 Col6a1 Col4a2 Col4a1 Sdc3 Itgb1
Up in TAC	Cardiovascular diseases	Dilated cardiomyopathy	$3.4X10^{-5}$	Myh7 Tgfb3 Itga11 Itga5 Igf1 Adcy4 Lmna Tpm4 Itgb1 Des Mybpc3
Up in TAC	Cardiovascular diseases	Hypertrophic cardiomyopathy	$7.4X10^{-5}$	Myh7 Tgfb3 Itga11 Itga5 Igf1 Lmna Tpm4 Itgb1 Des Mybpc3
Up in TAC	Cellular processes	Regulation of actin cytoskeleton	$3.4X10^{-4}$	Itga11 Itga5 Arhgef1 2900073G15Rik Actn1 F2r Myh10 Pdgfrb Rras Arpc4 Pip4k2a Csk Actn4 Arpc5 6720456B07Rik Itgb1
Down in TAC	Cellular processes	PPAR signaling	$1.6X10^{-5}$	Angptl4 Slc27a1 Acsl6 Acsl1 Acadm Cpt2 Aqp7 Rxrg Dbi Pparg Scp2
Down in TAC	Metabolism	Citrate cycle (TCA cycle)	$1.5X10^{-4}$	Suclg2 Sdhc Idh1 Cs Idh3a Pdha1
Down in TAC	Metabolism	Fatty acid metabolism	$3.6X10^{-4}$	Acsl6 Acaa2 Acsl1 Dci Acadm Acadvl Cpt2

LXRα-Tg sham versus LXRα-Tg TAC

Up/Down	Category	Subcategory	P-value	Genes
Up in TAC	Cellular processes	Focal adhesion	$8.5X10^{-6}$	Myl7 Col1a1 Col4a1 Col4a2 Ccnd2 Col5a1 Itga1 2900073G15Rik Capn2 Vav3 Flna Ccnd3 Actb
Up in TAC	Environmental information processes	ECM-receptor interaction	$2.3X10^{-4}$	Col1a1 Cd44 Col4a1 Col4a2 Col5a1 Itga1 Hspg2
Up in TAC	Cardiovascular	Hypertrophic cardiomyopathy	$2.3X10^{-4}$	Cacnb3 Itga1 Des Cacna2d1 Lmna Actb Ttn

	diseases			
Up in TAC	Cardiovascular diseases	Dilated cardiomyopathy	$4.2X10^{-4}$	Cacnb3 Itga1 Des Cacna2d1 Lmna Actb Ttn
Up in TAC	Cellular processes	Regulation of actin cytoskeleton	$1.8X10^{-3}$	Myl7 Itga1 Myh9 2900073G15Rik Arpc5l Vav3 Arpc3 Arhgef6 Msn Actb
Down in TAC	Metabolism	Drug metabolism – cytochrome P450	$2.0X10^{-11}$	Cyp2c50 Cyp2c37 Cyp2a5 Cyp2c70 Cyp2d9 Ugt2b36 Cyp2e1 Cyp2d10 Adh1 Ugt1a10 Gsta3 Mgst1 Gstt1 Fmo5 Gstz1 Gstt2
Down in TAC	Cellular processes	PPAR signaling	$1.8X10^{-9}$	Slc27a2 Acaa1b Apoc3 Cyp4a12a Apoa2 Apoa1 Hmgcs2 Cyp8b1 Acox2 Scd1 Cyp27a1 Rxrg Cpt2 Acsl5
Down in TAC	Metabolism	Fatty acid metabolism	$8.0X10^{-6}$	Acaa1b Cyp4a12a Adh1 Acat2 Acaa2 Acadvl Cpt2 Acsl5

Wt TAC versus LXRα-Tg TAC

**Up/Down**	**Category**	**Subcategory**	**P-value**	**Genes**
Up in LXRα-Tg	Metabolism	Metabolic pathways	$7.1X10^{-4}$	Asns St3gal5 Dbh Suclg2 Amy1 Abat Pla2g5 P4ha1 Gcnt1 Cyp27a1 Gck Ppt2 Gpaa1 Pip5k1b Rrm1 Nme4 Atp6v0a1 Chka Pla2g12a Fdps Nt5m Aprt Cox15 Lias Agps Pip5k1a LOC100040592 Pcyt2 Pigx Mtap Thtpa Grhpr
Up in LXRα-Tg	Metabolism	Glutathione metabolism	$1.1X10^{-3}$	Gstm1 Gstm6 Rrm1 Gstm4 Gstm2

Supplemental Table 4. Biometrical, Echocardiographic, and Hemodynamic Parameters of LXRα$^{-/-}$ and Wild-type Mice at Five Weeks Post Transverse Aortic Constriction

	**Sham**		**TAC**	
	**WT**	**LXRα$^{-/-}$**	**WT**	**LXRα$^{-/-}$**
Post mortem	(n=10)	(n=8)	(n=12)	(n=10)
Body weight, g	27.9 ± 0.6	25.8 ± 0.5‡	27.3 ± 0.4	27.7 ± 0.4
LV weight, mg	111.3 ± 2.4	100.9 ± 1.3	143.6 ± 8.6$^{\#}$	153.0 ± 8.5†
Liver weight, mg	1426 ± 43	1329 ± 54	1451 ± 34	1469 ± 56
Echocardiography	(n=10)	(n=7)	(n=12)	(n=10)
Interventricular septum, mm				
Diastole	0.73 ± 0.01	0.71 ± 0.02	0.99 ± 0.02†	1.05 ± 0.03†
Systole	1.49 ± 0.07	1.44 ± 0.08	1.48 ± 0.05†	1.34 ± 0.09†
LV posterior wall, mm				
Diastole	0.73 ± 0.01	0.72 ± 0.02	1.02 ± 0.03	1.04 ± 0.04
Systole	1.34 ± 0.06	1.59 ± 0.11	1.36 ± 0.06	1.21 ± 0.05$^{\#}$
LV internal diameter, mm				
Diastole	3.85 ± 0.08	3.75 ± 0.08	3.86 ± 0.13	3.84 ± 0.10
Systole	2.24 ± 0.06	2.26 ± 0.08	2.90 ± 0.15$^{\#}$	3.08 ± 0.10†
Fractional shortening, %	41.9 ± 0.6	39.8 ± 1.2	25.3 ± 1.8	19.5 ± 1.9
E/A ratio	1.7 ± 0.1	1.6 ± 0.1	1.7 ± 0.1†	1.7 ± 0.1†
Hemodynamics	(n=10)	(n=7)	(n=10)	(n=9)
Heart rate, bpm	482 ± 17	477 ± 25	465 ± 22	472 ± 14
Mean arterial pressure, mmHg	72.7 ± 2.3	73.1 ± 2.8	86.6 ± 3.0$^{\#}$	93.1 ± 1.7†
LV end-systolic pressure, mmHg	95.4 ± 2.5	98.1 ± 3.6	131.3 ± 3.2†	141.5 ± 5.1†
LV end-diastolic pressure, mmHg	10.0 ± 0.9	9.2 ± 2.1	18.8 ± 1.4$^{\#}$	21.6 ± 1.9†
dP/dt_{max}, mmHg	6995 ± 327	7757 ± 612	6279 ± 266	6751 ± 323
dP/dt_{min}, mmHg	-6815 ± 232	-7496 ± 608	-6089 ± 248	-6447 ± 415
Tau, τ, msec	7.2 ± 0.2	6.8 ± 0.5	8.9 ± 0.4*	9.8 ± 0.6†

Data are expressed as means ± SEM. * P<0.05, $^{\#}$ P<0.05, † P<0.001, TAC versus corresponding sham group; ‡ P<0.05, WT vs LXRα$^{-/-}$ mice.

Supplemental Table 5. Mouse Gene Primers for Real-Time PCR

Gene	Forward primer (5'-3')	Reverse primer (5'-3')
Lxra	ATGCGGCGGAAATGCCAGGA	TACACTGTTGCTGGGCAGCC
Lxrb	CCGAAGATGCTGGGCCATGA	CATGCCAGCCTCCTTGCACT
Pgc1a	AACGCGGACAGAATTGAGAG	TTCGTTCGACCTGCGTAAAG
Srebp1c	ATCGGCGCGGAAGCTGTCGGGGTAGCGTC	ACTGTCTTGGTTGTTGATGAGCTGGAGCAT
Scd1	GCGCATCTCTATGGATATCG	TACTCACTGGCAGAGTAGTC
Abca1	GGACATGCACAAGGTCCTGA	CAGAAAATCCTGGAGCTTCAAA
Abcg1	CCTTCCTCAGCATCATGCG	CCGATCCCAATGTGCGA
Myh6	GTTAACCAGAGTTTGAGTGACA	CCTTCTCTGACTTTCGGAGGTACT
Myh7	ATGTGCCGGACCTTGGAAG	CCTCGGGTTAGCTGAGAGATCA
Nppa	ATGGGCTCCTTCTCCATCAC	TCTACCGGCATCTTCTCCTC
Nppb	AAGTCCTAGCCAGTCTCCAGA	GAGCTGTCTCTGGGCCATTTC
Acta1	TGCCATGTATGTGGCTATCCA	TCCCCAGAATCCAACACGAT
Rcan1	GCTTGACTGAGAGAGCGAGTC	CCACACAAGCAATCAGGGAGC
Col1a1	ACAGCGTAGCCTACATGG	AAGTTCCGGTGTGACTCG
Ctgf	GCATCTCCACCCGAGTTAC	ACTGGTGCAGCCAGAAAG
Cd36	CTGTGTTTGGAGGCATTC	AGCAGTGGTTCCTTCTTC
Fabp3	GAGGCAAACTCATCCATGTG	ACGCCTCCTTCTCATAAGTC
Fasn	AGCTACCGGCAAAGATGAC	CCCGATCTTCCAGGCTCTTC
Hsl	ACACGTCACCCATAGTCAAG	ACATGACCGAGTCATCTAGC
Lpl	GGGCTCTGCCTGAGTTGTAG	CCATCCTCAGTCCCAGAAAA
Atgl	GGCCAAGAGGAAATTGGG	AAGACAGTGGCACAGAGG
Ppara	TATTCGGCTGAAGCTGGT	CTGGCATTTGTTCCGGTTCT
Acc2	ATCTGAAGCGGGACTCTG	AGCTGAGCCACCTGTATC
Mcd	GCTCTGACTGGTGACATTTC	CTGTTCCTCTCCTCGGTTTC
Cpt1a	CCTGCATTCCTTCCCATTTG	AGTCATGGAAGCCTCATACG
Cpt1b	CCCATGTGCTCCTACCAG	CACGTGCCTGCTCTCTGA
Mcad	CCGTTCCCTCTATCAAAAG	ACACCCATACGCCAACTCTT
Lcad	ATGTGGGAGTACCCGATTG	TCCAGGATGTAGGCAGATG
Ucp3	TGGTGACCTACGACATCATC	CCAAAGGCAGAGACAAAGTG
Glut1	GGTGTGCAGCAGCCTGTGTA	GACGAACAGCGACACCACAGT
Glut4	GACAGCCAGCCTACGCACCA	TGTCCGTCGTCCAGCCTCGTTCTAC
Hk2	GGACGGGACACTGTACAAG	GCCACAGCAGTGATGAGAG
Pfkm	CTGGTCTTTCAGCCAGTAAC	GAGGTGTCCAGATCAATCTC
Pdk4	GCATTTCTACTCGGATGCTCATG	CCAATGTGGCTTGGGTTTCC
Pepck	TTTGTAGGAGCTGCCATGAG	TGCCGAAGTTGTAGCCAAAG
Chrebp	AGCTGCGGGATGAAATAGAG	ATCTGGTCAAAGCGCTGATG
G6pd	GCCTGCCATGTTCTTTAACC	CCAGGATGAGGCGTTCATAC
IL6	TCCCAACAGACCTGTCTATAC	CAGAATTGCCATTGCACAACTC

Mcp1	CAATGAGTAGGCTGGAGAG	CTGGACCCATTCCTTCTTG
Cd68	CTCTCATCATTGGCCTGGTC	GGGCTGGTAGGTTGATTGTC
Cox2	GCAAAGGCCTCCATTGAC	TCTGCAGCCATTTCCTTC
Bcl2	TGGGATGCCTTTGTGGAACT	CTGAGCAGCGTCTTCAGAGACA
Bax	CATCATGGGCTGGACACTGG	GTCCCGAAGTAGGAGAGGAG
36b4	AAGCGCGTCCTGGCATTGTC	GCAGCCGCAAATGCAGATGG

Supplemental Table 6. Rat Gene Primers for Real-Time PCR

Gene	Forward primer (5'-3')	Reverse primer (5'-3')
Glut1	GCTTTGG CAGGCGGAAC T	AGCACGGCAGACACAAAGG
Glut4	CCGTGGCCTCCTATGAGATACT	AGGCACCCCGAAGATGAGT
Nppa	ATGGGCTCCTTCTCCATCAC	TCTACCGGCATCTTCTCCTC
Nppb	ACAATCCACGATGCAGAAGCT	GGGCCTTGGTCCTTTGAGA
Acta1	GGGCAGGTCATCACCATC	TACAGGTCCTTCCTGATGTC
Rcan1	CCGACTCATTGGGATCATCTT	TGTCTCAGCCAGATGCAGTTAAT
36b4	AAGCGCGTCCTGGCATTGTC	GCAGCCGCAAATGCAGATGG

Supplemental Table 7. Primers used for Cloning

Gene	5'-3'
LXRα forward	GTT GGATCC ACC ATGTCCTTGTGGCTGGAGG
LXRα reverse	GAA CTCGAG TCA TTCGTGGACATCCCAGATC
siLXRα forward	GATCCCGGAGTGTCGCCTTCGCAAATTCAAGAGATTTGCGAAGGCGACACTCCTTTTTGGAAA
siLXRα reverse	AGCTTTTCCAAAAAGGAGTGTCGCCTTCGCAAATCTCTTGAATTTGCGAAGGCGACACTCCGG
