# Supplementary figures and images for: Cardiac LXRα protects against pathological cardiac hypertrophy and dysfunction by enhancing glucose uptake and utilization
Source: EMBO Mol Med. 2015 Jul 14;7(9):1229–43. doi: 10.15252/emmm.201404669 (PMC4568954; doi:10.15252/emmm.201404669)

**Full unedited blots for Suppl Fig 1, panel J**

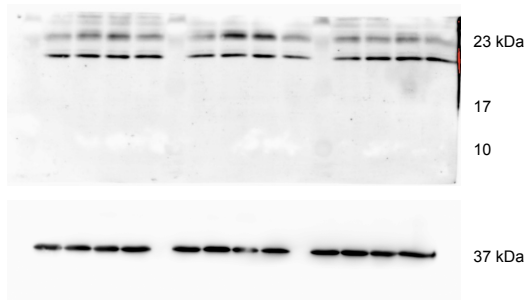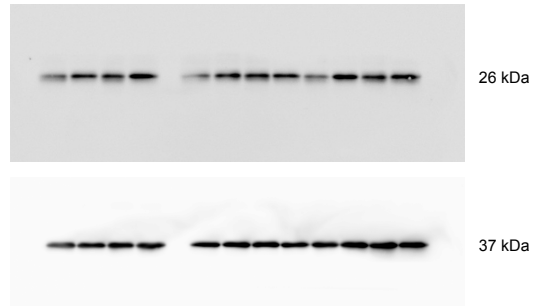

Supplement: Supplementary file 2 [file emmm0007-1229-sd2.pdf]

## Full unedited blots for Suppl Fig 9, panel B

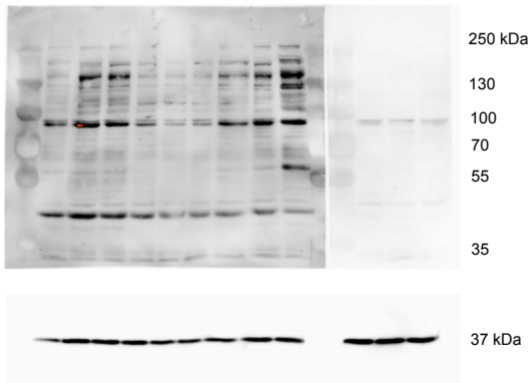

Supplement: Supplementary file 4 [file emmm0007-1229-sd4.pdf]

Cannon et al. Source Data

Full unedited blot for Figure 1, panel B

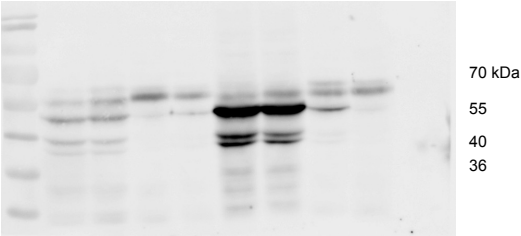

Supplement: Supplementary file 6 [file emmm0007-1229-sd6.pdf]

Full unedited blots for Figure 4, panel E

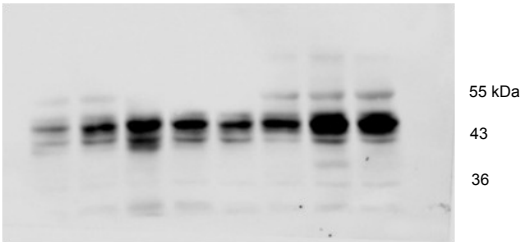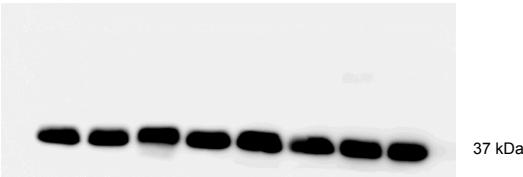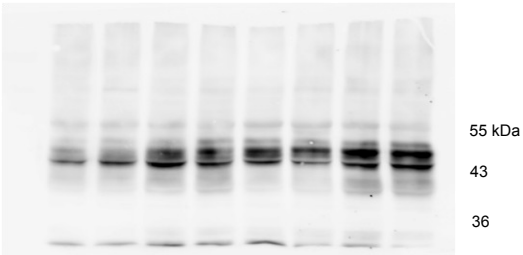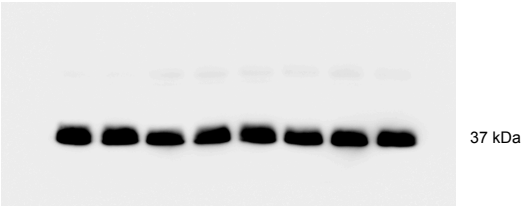

Supplement: Supplementary file 7 [file emmm0007-1229-sd7.pdf]

Full unedited blots for Figure 5, panel H

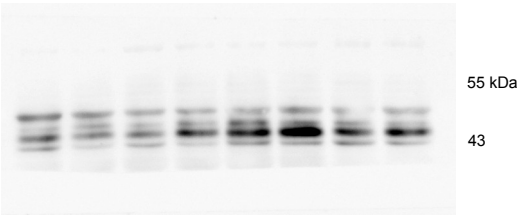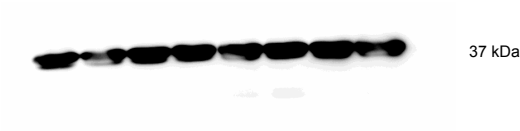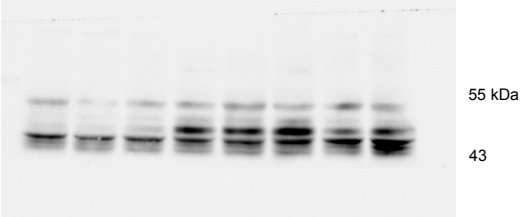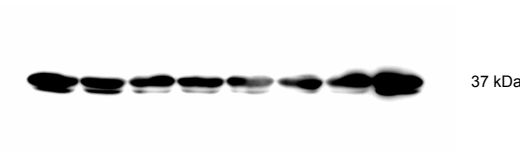

Supplement: Supplementary file 8 [file emmm0007-1229-sd8.pdf]

Full unedited blots for Figure 6, panel D

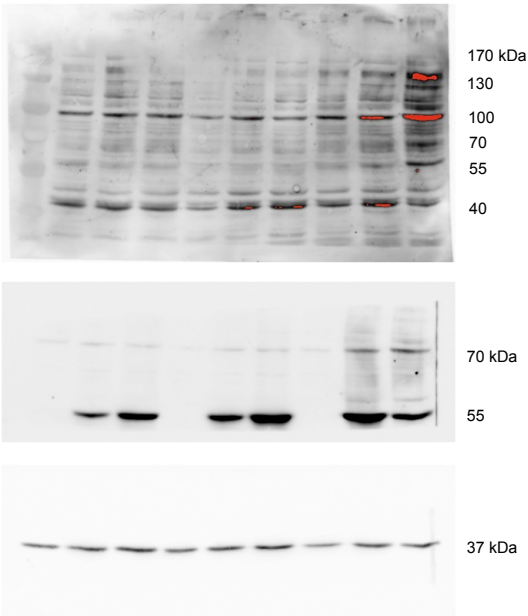

Supplement: Supplementary file 9 [file emmm0007-1229-sd9.pdf]

Full unedited blots for Figure 7, panel B

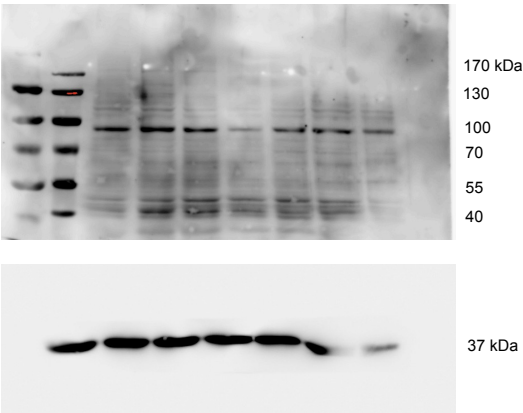

Supplement: Supplementary file 10 [file emmm0007-1229-sd10.pdf]
