# Supplementary material for: Cardiac LXRα protects against pathological cardiac hypertrophy and dysfunction by enhancing glucose uptake and utilization
Source: EMBO Mol Med. 2015 Jul 14;7(9):1229–43. doi: 10.15252/emmm.201404669 (PMC4568954; doi:10.15252/emmm.201404669)
Supplement: Supplementary file 3 [file emmm0007-1229-sd3.pdf]

Cannon et al. Source Data_Supplementary Material

Full unedited blots for Suppl Fig 5, panel A

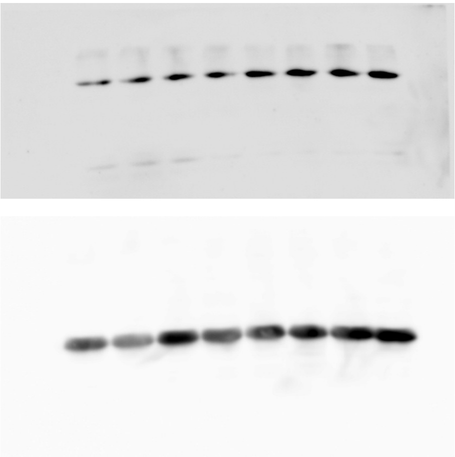

130 kDa
100
37 kDa

Full unedited blots for Suppl Fig 5, panel B

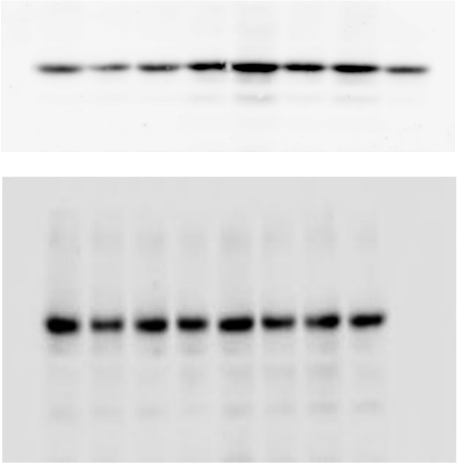

60 kDa
60 kDa

Full unedited blots for Suppl Fig 5, panel C

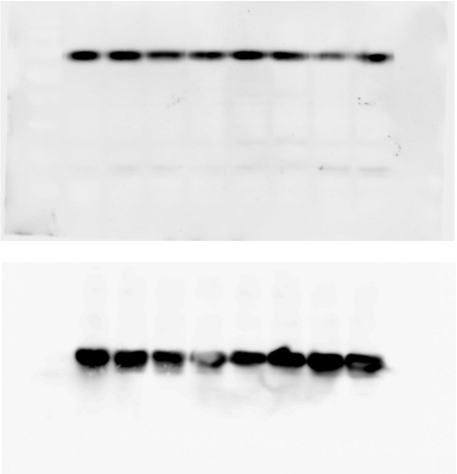

85 kDa
37 kDa
