# Supplementary material for: Cardiac LXRα protects against pathological cardiac hypertrophy and dysfunction by enhancing glucose uptake and utilization
Source: EMBO Mol Med. 2015 Jul 14;7(9):1229–43. doi: 10.15252/emmm.201404669 (PMC4568954; doi:10.15252/emmm.201404669)
Supplement: Supplementary file 5 [file emmm0007-1229-sd5.pdf]

Cardiac LXR protects against pathological cardiac hypertrophy and dysfunction by enhancing glucose uptake and utilization

Megan V. Cannon, Herman H.W. Silljé, Jürgen W.A. Sijbesma, Inge Vreeswijk-Baudoin, Jolita Ciapaite, Bart van der Sluis, Jan van Deursen, Gustavo J.J. Silva, Leon J. de Windt, Jan-Åke Gustafsson, Pim van der Harst, Wiek H. van Gilst, Rudolf A. de Boer

Corresponding author: Rudolf de Boer, University Medical Center Groningen

Review timeline:

Submission date:	19 September 2014
Editorial Decision:	22 October 2014
Revision received:	16 April 2015
Editorial Decision:	08 May 2015
Revision received:	04 June 2015
Accepted:	12 June 2015

Transaction Report:

(Note: With the exception of the correction of typographical or spelling errors that could be a source of ambiguity, letters and reports are not edited. The original formatting of letters and referee reports may not be reflected in this compilation.)

Editor: Roberto Buccione

1st Editorial Decision 22 October 2014

Thank you for the submission of your manuscript to EMBO Molecular Medicine. We have now heard back from two Reviewers whom we asked to evaluate your manuscript.

We are sorry that it has taken longer than usual to get back to you on your manuscript. In this case we experienced unusual difficulties in securing three appropriate reviewers and then obtaining their evaluations in a timely manner and furthermore, one Reviewer unexpectedly withdrew (#2).

As you will see, the two Reviewers find merits in your manuscript but raise significant issues. Based on our discussion here, we feel that while there is consensus that your work is novel and potentially interesting, the main concerns are the insufficient depth of mechanistic insight and that the metabolic implications appear to be unclear/unproven. I will not dwell into much detail, as the Reviewers' comments are clear. I would like, however, to highlight a few main points.

Reviewer 1 would like more insight into how LXRalpha impacts hypertrophy and heart function and is also puzzled by the apparently contradiction between the finding that LXRalpha expression exacerbates glucose-glucose oxidation uncoupling and the suggestion of a beneficial metabolic effect in the heart. S/he also lists other issues that require your action.

Reviewer 3 challenges your decision to focus on a specific time point after TAC surgery and suggests that earlier phases should be also analysed to clarify the role of LXRalpha. S/he also finds that the loss-of-function experiments do not evidence an especially remarkable phenotype, as compared to LXRalpha overexpression. Reviewer 3 also lists other items for your consideration.

In conclusion, while publication of the paper cannot be considered at this stage, given the potential interest of your findings and the fact that the Reviewers, although critical, were globally positive, we have decided to give you the opportunity to address the above concerns.

We are thus prepared to consider a substantially revised submission, with the understanding that the Reviewers' concerns must be addressed with additional experimental data where appropriate and that acceptance of the manuscript will entail a second round of review. This also includes significantly upgrading the level of mechanistic insight and clarifying the metabolic implications.

Please note that it is EMBO Molecular Medicine policy to allow a single round of revision only and that, therefore, acceptance or rejection of the manuscript will depend on the completeness of your responses included in the next, final version of the manuscript.

As you know, EMBO Molecular Medicine has a "scooping protection" policy, whereby similar findings that are published by others during review or revision are not a criterion for rejection. However, I do ask you to get in touch with us after three months if you have not completed your revision, to update us on the status. Please also contact us as soon as possible if similar work is published elsewhere.

I look forward to seeing a revised form of your manuscript in due time.

***** Reviewer's comments *****

Referee #1 (Comments on Novelty/Model System):

This is an interesting paper that provides intriguing data to support the concept that LXRa overexpression in the heart can decrease the severity of cardiac hypertrophy and improve cardiac function in mice subjected to TAC or Ang II infusion. It is proposed that this occurs via increasing myocardial glucose uptake and increasing protein O-GlycNAcylation. It is suggested that modulating LXRa may provide a unique opportunity for intervening in myocyte metabolism.

The data showing a beneficial effects of over-expressing LXRa in the heart on decreasing hypertrophy and increasing cardiac function is convincing, and the experiments are competently performed. Similarly, the effect of LXRa deletion on exacerbating hypertrophy is also convincing. However, the mechanistic insights into how LXRa overexpression and deletion are having these effects is less clear. Data is provided to show that LXRa overexpression increases glucose uptake and protein O-GlycNAcylation. However, how this may alter cardiac hypertrophy and cardiac function is not clear.

Many studies have shown that cardiac hypertrophy is associated with an increase in glucose uptake by the heart, a phenomena consistent with the switch of the heart to a more "fetal" phenotype. This is confirmed in the present study. What is confusing is that cardiac LXRa overexpression actually further increases glucose uptake and GLUT1 and GLUT4 expression. This occurs without any increase in pyruvate oxidation. As a result, the uncoupling of glycolysis from glucose oxidation is

exacerbated by LXRa overexpression. It is not clear how this would have a beneficial metabolic effect in the hearts. The authors suggest that this additional glucose for the heart is imporetant. However, what is really required is an increase in mitochondrial oxidative metabolism, particularly glucose oxidation. Unfortunately, LXRa-overexpression did not really alter these pathways, and based on the PDK4 expression may have further decreased glucose oxidation. The authors do not adequately address this paradox.

An increase in ANP and BNP is a hallmark of cardiac hypertrophy, and the levels of these naturetic peptides has been used as a marker of hypertrophy severity. Curiously, LXRa overexpression markedly increased both ANP and BNP expression. The authors suggest that this is cardioprotective, but provide no evidence to support this.

Cardiac LXRa overexpression was shown to be associated with an increase in protein O-GlycNAcylation. This is convincing data. What is not clear, however, is how these changes impact on either cardiac hypertrophy or cardiac function. Data linking altered protein O-GlycNAcylation to hypertrophy of function is missing. In addition, the authors do not adequately speculate as to why alterations in protein O-GlycNAcylation would alter hypertrophy or function.

Specific Comments:

1) page 6, line 2. It is stated that LXRa overexpression increased PGC-1a expression. This should increase mitochondrial biogenesis and oxidative metabolism. However, this did not appear to occur in this study. The authors should address why this dissociation occurred.

2) page 13, line 13, The authors quote the study of Kolwicz and Tian (2011) to support the concept that increasing the reliance of the heart on glucose is an adaptive response to cardiac hypertrophy. However, this study actually showed an increase in glucose oxidation in the heart, while glycolysis actually decreased. This is opposite of what was seen in the authors study. This needs to be clarified in the paper.

Referee #1 (Remarks):

This paper provides interesting data to link LXRa to cardioprotection in the setting of cardiac hypertrophy. The beneficial effect of cardiospecific overexpression of LXR1 is convincing. However, what is less convincing is how LXRa overexpression is having these effects. The metabolic effects of LXRa are opposite to what would be expected. In addition, convincing evidence linking alterations in protein O-GlycNAcylation to decreased cardiac hypertrophy are not convincing. These and other concerns are discussed in detail in the "Comments to Authors".

Referee #3 (Comments on Novelty/Model System):

gain and loss of function studies were appropriately performed in mice

Referee #3 (Remarks):

Cannon et.al investigated the role of cardiac LXR for pathological cardiac hypertrophy and dysfunction. So far, very little has been known about the role of LXR in the heart, although its possible effect on blood pressure control has been recently reported. Using a transgenic approach as well as a LXR -deficient mouse model, the paper indicates that LXR acts as a transcriptional regulator that helps in the adaptive metabolic response to chronic cardiac stress by interacting with cardiac metabolism.

However, there are several points that need to be more precisely addressed:

Major comments:

1) The authors show some robust evidence that overexpression of LXR attenuates pathological hypertrophy in a TAC and in an angiotensin-dependent model. Despite the development of a robust phenotype that is protected against pathological remodeling in case of pressure overload, it remains unclear why the authors have decided to study animals only at one time point- namely 5 weeks upon TAC surgery. In a pressure overload model, different phases of cardiac remodeling occur, where different processes take place- early phase (up to 1 week after surgery) where inflammatory processes, apoptosis and necrosis take place, intermediate phase (up to 4 weeks after surgery with development of pathologic hypertrophy), as well as a late phase with cardiac decompensation and development of congestive heart disease. For deciphering of possible complementary mechanisms of LXR action, it would be interesting to know whether LXR has direct influence on other earlier processes in cardiac remodeling such as inflammation or cardiac apoptosis

2) Whereas the TG mouse model shows a robust cardioprotective response and shows that overexpression of LXR is sufficient to induce cardioprotection, the loss-of function model does only show a slightly more severe phenotype than wildtype animals in some but not all aspects (Figure 5A-G). How would the authors comment on that?

3) Figure 6D: The authors argue that the cardioprotective effect of LXR is to be contributed to the stimulation of HBP. Indeed the Western Blot they show shows an increased glycosylation with increased MOIs of Ad- LXR ; however the DON administration does not seem to lead to a significant reduction of the O-Glycosylation pattern of the transfected cells. Furthermore, instead of PE another positive control (such as treatment with Glucosamine oder Thiamet G) is more proper in this setting. Please provide the proper controls.

4) Indeed, in case the cardioprotective effect of LXR is HBP-dependent, one would expect a deterioration of the cardioprotective function after DON treatment in vivo. Is there any in vivo data to confirm this?

Minor comments:

1) It would be helpful to provide the n = number of animals per group directly in the figure itself and not only in the figure legend

2) The authors argue that O-Glycosylation and the shift towards glucose reliance is believed to be an adaptive response that confers cardioprotection, however , it is also known that increased O-glycosylation of certain proteins in the heart (Eriksson et al, 2013) also may have an unfavorable effect on heart function, arrhythmias and overall animal survival. Hence, it would be informative to have the Kaplan-Meyer curves of overall survival of the TG vs WT mice

3) Fig. 6E-F: is the labeling correct below the last column. Should this not read DON+PE?

1st Revision - authors' response 16 April 2015

RESPONSES TO REVIEWERS

Comments from Reviewer 1:

Referee #1 (Comments on Novelty/Model System):

This is an interesting paper that provides intriguing data to support the concept that LXRa overexpression in the heart can decrease the severity of cardiac hypertrophy and improve cardiac function in mice subjected to TAC or Ang II infusion. It is proposed that this occurs via increasing myocardial glucose uptake and increasing protein O-GlycNAcylation. It is suggested that modulating LXRa may provide a unique opportunity for intervening in myocyte metabolism.

The data showing a beneficial effects of over-expressing LXRa in the heart on decreasing hypertrophy and increasing cardiac function is convincing, and the experiments are competently performed. Similarly, the effect of LXRa deletion on exacerbating hypertrophy is also convincing. However, the mechanistic insights into how LXRa overexpression and deletion are having these effects is less clear. Data is provided to show that LXRa overexpression increases glucose uptake and protein O-GlycNAcylation. However, how this may alter cardiac hypertrophy and cardiac function is not clear.

We are pleased to read that the article is of interest to the reviewer, and would like to thank the reviewer for his/her careful consideration and critique of our manuscript. On the basis of the remarks provided by the reviewer, we have conducted additional experiments and analyses, which further strengthen the conclusions regarding modulation of cardiac hypertrophy by LXRα overexpression. We have revised the manuscript accordingly, as described below.

1) Many studies have shown that cardiac hypertrophy is associated with an increase in glucose uptake by the heart, a phenomena consistent with the switch of the heart to a more "fetal" phenotype. This is confirmed in the present study. What is confusing is that cardiac LXRa overexpression actually further increases glucose uptake and GLUT1 and GLUT4 expression. This occurs without any increase in pyruvate oxidation. As a result, the uncoupling of glycolysis from glucose oxidation is exacerbated by LXRa overexpression. It is not clear how this would have a beneficial metabolic effect in the hearts. The authors suggest that this additional glucose for the heart is imporetant. However, what is really required is an increase in mitochondrial oxidative metabolism, particularly glucose oxidation. Unfortunately, LXRa-overexpression did not really alter these pathways, and based on the PDK4 expression may have further decreased glucose oxidation. The authors do not adequately address this paradox.

We agree that some aspects of the LXRα transgenic mouse appear paradoxical. Our LXRα-Tg mice show enhanced glucose uptake in the heart, both under basal and stressed conditions, but we cannot agree with the statement that "the uncoupling of glycolysis from glucose oxidation is exacerbated by LXRα overexpression." In these hearts, glucose oxidation is not reduced or impaired, but remains

intact. Our data indicate that mitochondrial oxidation in the presence of the glucose substrate, pyruvate, was normal, not compromised, when compared to wild-type mice (Suppl. Figure 7A, included below). Furthermore, contractile function was not impaired in LXRα-Tg mice, neither at baseline (Suppl. Table I), or following chronic pressure overload (data added to Suppl. Table II). This suggests that, in LXRα-Tg hearts, mitochondrial ATP is sufficient to support myocardial contraction, and that ATP supply matches energy demand.

Supplemental Figure 7A:

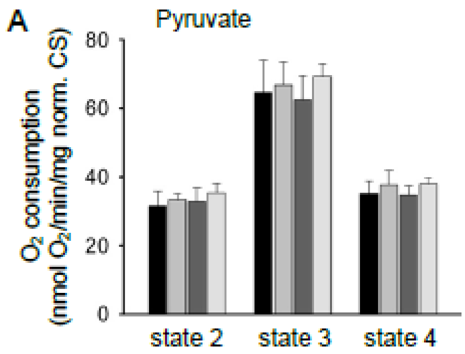

In overt heart failure, mitochondrial function is impaired and both glucose and fatty acid oxidation rates decline. Under these conditions, increasing mitochondrial metabolism would obviously be beneficial for the heart. However, during the development of hypertrophy and early heart failure, the uncoupling of glycolysis from glucose oxidation is not yet present. A study by Allard et al. showed that glucose oxidation rates are normal in hypertrophic hearts, whereas glycolytic rates were substantially higher compared to control hearts *(Heart Circ Physiol* 1994;36:H742-50). Also, a recent paper by Lai et al. showed that expression of mitochondrial OXPHOS components were not altered in early heart failure (*Circ Heart Fail* 2014;7:1022-31). We have not performed studies inducing failure in LXRα-Tg hearts, where these phenomena are expected to occur, as the objective of our current study was to investigate the effect of LXRα overexpression on hypertrophic remodeling. This would certainly be the next step in our investigations. Nevertheless, it is interesting that we observed a trend for increased fatty acid oxidation capacity in LXRα-Tg hearts (Suppl. Fig. 7C). Since our studies were performed in the setting of hypertrophic development and our mice did not show signs and symptoms of overt heart failure, the observed increases in glucose uptake and relative to glucose oxidation is therefore in agreement with previous reports (Allard et al., *Heart Circ Physiol* 1994;36:H742-50; Wambolt et al., *J Mol Cell Cardiol* 1999;31:493-502).

The uncoupling between glucose uptake rates and glucose oxidation is intriguing, and this apparent paradox has led to an interest in exploring other novel uses for derivatives of glycolysis (reviewed by Kolwicz and Tian, *Cardiovasc Res* 2011;90:194-201; Doenst et al., *Circ Res* 2013;113:709-724). In our mouse model, we observed an increase in glucose uptake that was diverted toward the hexosamine biosynthesis pathway, resulting in increased levels of O-GlcNAcylated proteins. Amongst these O-GlcNAcylated proteins, transcription factors are present that control the expression of natriuretic peptides (new data, see below). In LXRα-Tg mice, the identification of these O-GlcNAc targets

establish the link between glycolysis and the capacity to induce transcription of potent anti-hypertrophic factors, natriuretic peptides, via glucose-O-GlcNAc-dependent signaling. We believe that this is in large part the mechanism for protection against cardiac hypertrophy in these mice.

2) An increase in ANP and BNP is a hallmark of cardiac hypertrophy, and the levels of these naturetic peptides has been used as a marker of hypertrophy severity. Curiously, LXRa overexpression markedly increased both ANP and BNP expression. The authors suggest that this is cardioprotective, but provide no evidence to support this.

Although natriuretic peptides are commonly used as biomarkers of heart failure severity, and thus elevated levels are usually linked to severe heart failure, however, in and of themselves, natriuretic peptides are *protective* molecules functioning naturally to prevent heart failure development.

The effects of natriuretic peptides on cardiac hypertrophy have been well established. Deletion of ANP in mice causes hypertension and cardiac hypertrophy at baseline, whereas pressure and volume overload cause an exacerbated hypertrophic response in mutant mice more than wild-type (Mori et al., *Cardiovasc Res* 2003;61(4):771-9; Wang et al., *Hypertension* 2003;42:88-95). When these mice were fed a low-salt diet, differences in blood pressure (BP) disappeared, but mice deficient for ANP still had extensive hypertrophy, demonstrating that the anti-hypertrophic effects of ANP are BP-independent (Feng et al., *Clin Exp Pharmacol Physiol* 2003;30:343-9). In studies using NPR-A-knockout mice (NPR-A is the receptor for ANP and BNP), these mice suffer from ventricular hypertrophy, interstitial fibrosis, and cardiac dysfunction, also demonstrating that abolished natriuretic peptide signaling has detrimental effects on the heart independent of elevated BP (Kishimoto et al., *Proc Natl Acad Sci USA* 2001;98(5):2703-6; Knowles et al., *J Clin Invest* 2001;107(8):975-84). Furthermore, specific deletion of NPR-A in the heart using an α-MHC-Cre system confirmed the direct hypertrophic effect on the myocardium (Holtwick et al., *J Clin Invest* 2003;111:1399–1407). More recently, a novel drug class of angiotensin receptor neprilysin inhibitors (ARNi) have been developed to simultaneously block RAAS activation and augment natriuretic peptides through neprilysin inhibition, an enzyme that degrades active natriuretic peptides. LCZ696, an ARNi, was shown to attenuate cardiac hypertrophy and dysfunction post myocardial infarction in rats (Krum et al., *Circ Heart Fail* 2015;8:71-8). In humans, the PARADIGM study has been published recently (McMurray et al., NEJM 2014), showing that LCZ696 reduces heart failure-related morbidity and mortality that was associated with a striking *increase* in circulating BNP. Taken together, these studies strongly support the notion that natriuretic peptides are cardioprotective, and this is widely accepted in literature. Thus, the induction of ANP and BNP in LXRα-Tg hearts may very well explain the overall protection against damaging perturbations.

We have added the following text with reference in the Results section:

"The anti-hypertrophic properties of ANP and BNP are well established (Nishikimi et al. 2006)."

3) Cardiac LXRa overexpression was shown to be associated with an increase in protein O-GlycNAcylation. This is convincing data. What is not clear, however, is how these changes impact on either cardiac hypertrophy or cardiac function. Data linking altered protein O-GlycNAcylation to hypertrophy of function is missing. In addition, the authors do not adequately speculate as to why alterations in protein O-GlycNAcylation would alter hypertrophy or function.

We agree that the link between global O-GlcNAcylation and hypertrophy was not clear. We have therefore performed additional experiments to clarify this effect. We first conducted experiments *in vitro* and show that LXRα overexpression in cardiomyocytes reduced cell size following hypertrophic stimulation with PE, but in the presence of DON, an inhibitor of the hexosamine biosynthetic pathway (HBP) and O-GlcNAc formation, the effect on reduced cell size was abolished (Figure 6G and 6H).

We have added the following figures and text to the revised manuscript:

Figure 6G and 6H:

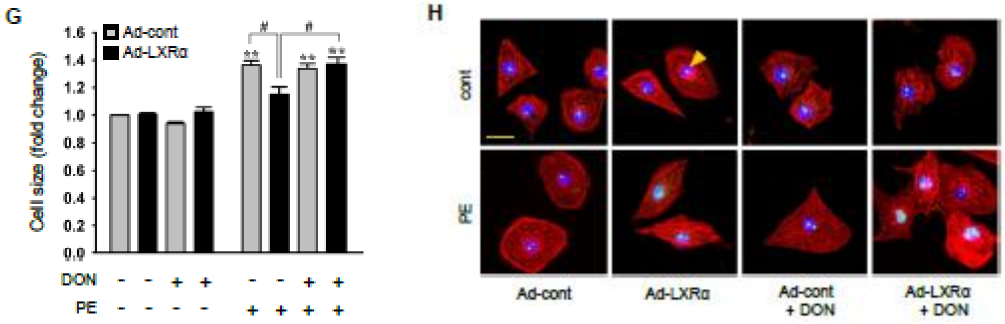

"Further assessment of cellular hypertrophy indicated that DON inhibition of HBP flux also abolished the Ad-LXRα-mediated reductions in cell size that was increased upon PE stimulation (Figure 6G and 6H)."

Secondly, we acknowledge that global O-GlcNAcylation may be merely informative in that it represents an abundance of unspecified O-GlcNAcylated proteins present in the myocardium. Since we show that LXRα overexpression increases ANP and BNP both *in vivo* and *in vitro*, and inhibiting O-GlcNAc formation with DON reduced the upregulated ANP and BNP (Figure 6E and 6F), suggesting that their transcription is dependent on this pathway, we therefore looked to identify specific O-GlcNAcylated targets. We performed wheat germ agglutinin (WGA) precipitation to isolate nuclear O-GlcNAcylated proteins, and with Western blot we show that known transcriptional activators of both ANP and BNP, GATA4 and Mef2c, but not Nkx-2.5 (Figure 8C and 8D), were glycosylated. This suggests that O-GlcNAc modification enhances their transcriptional activity toward natriuretic peptides, ANP and BNP, linking glucose metabolism with induction of these natriuretic peptides to confer anti-hypertrophic effects in the heart. We also believe that these findings advance the current understanding of these pathways that are not typical of the energy-dependent functions of glucose, yet beneficial in the functioning and survival of the myocyte in pathological hypertrophy.

We have added the following figures and text to the Results section:

<u>Figure 8C and 8D</u>:

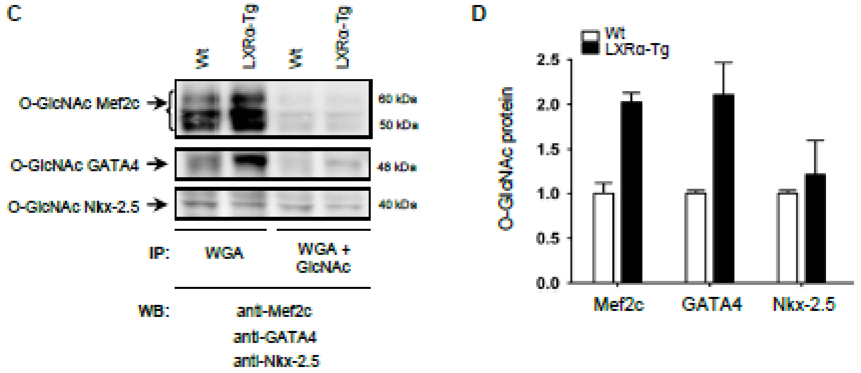

"To further identify specific O-GlcNAc targets, agarose wheat germ agglutinin (WGA) precipitation was performed to isolate nuclear GlcNAcylated proteins. Using antibodies specific for known transcription factors activating ANP and BNP (Hayek and Nemer. 2011,Morin et al. 2000), Western blot analysis revealed that GATA4 and Mef2c precipitated with WGA in LXRα-Tg hearts, but not with Nkx-2.5, suggesting that O-GlcNAc modification of GATA4 and Mef2c potentiate their activities (Figure 8C and 8D). N-acetylglucosamine (GlcNAc), a competitor, served as control. In summary, these data indicate that cardiac LXRα integrates glucose metabolism and downstream O-GlcNAcylation with induction of cytoprotective natriuretic peptides to orchestrate an anti-hypertrophic response."

Specific Comments:

1) page 6, line 2. It is stated that LXRa overexpression increased PGC-1a expression. This should increase mitochondrial biogenesis and oxidative metabolism. However, this did not appear to occur in this study. The authors should address why this dissociation occurred.

We were indeed expecting to see these changes, particularly for mitochondrial citrate synthase activity and oxidative metabolism (Suppl. Fig. 7) as a result of increased PGC1a expression. On the other hand, we believe that PGC1a may perform other functions in the context of LXR biology. The transcriptional activities of LXRs are highly regulated by bound co-factors that keep them either in the active or inactive state. PGC1a is an established co-activator of LXRs (Oberkofler et al., *Biochem J* 2003;371:89-96), and when bound to the LXR/RXR heterodimer complex, PGC1a enables LXR to activate target gene expression. We therefore speculate that the increased PGC1a may be a direct effect of, or an intrinsic response to, the increased cardiac LXRα expression induced in transgenic mice, assisting the LXRα transgene in functionally maintaining a state of constitutive activation, as opposed to playing a role in mitochondrial pathways.

We have added the following text to the Results section:

"To verify whether overexpression indeed induced functionally active LXRα, we determined mRNA levels of well described LXRα target genes (Tontonoz and Mangelsdorf. 2003) including Srebp1c, Scd1,

and Abca1. These were significantly increased in LXRα-Tg mice (Figure 1H). Furthermore, Pgc1a, a co-activator of LXR (Oberkofler et al. 2003), was also upregulated, plausibly to maintain LXRα in a constitutively active state."

2) page 13, line 13, The authors quote the study of Kolwicz and Tian (2011) to support the concept that increasing the reliance of the heart on glucose is an adaptive response to cardiac hypertrophy. However, this study actually showed an increase in glucose oxidation in the heart, while glycolysis actually decreased. This is opposite of what was seen in the authors study. This needs to be clarified in the paper.

We did not quote a research paper, but a review article by S. Kolwicz and R. Tian (2011) that provides support for enhanced glucose reliance (energy-dependent or energy-independent) as an adaptive response in cardiac hypertrophy.

We do not argue against an increase in glucose oxidation being beneficial. We do, however, want to emphasize that there are several studies showing that an increase in glucose uptake does not necessarily result in an increase in glucose oxidation in hypertrophic hearts (mentioned in the Discussion section, page 15, line 18): *"Moreover, the fate of glucose is of interest given that increased glucose uptake and glycolysis in cardiac hypertrophy does not always result in concomitant increases in glucose oxidation (Allard et al, 1994; Wambolt et al, 1999; Doenst et al, 2013)."* Our study further addresses this phenomenon, where the increase in glucose uptake relative to glucose oxidation is particularly evident with cardiac LXRα overexpression.

The studies of Liao et al. and Luptak et al. mentioned on page 14, line 20, below this quote also point to the same beneficial effects of glucose uptake in adaptation to cardiac hypertrophy. Liao et al. generated a transgenic mouse with constitutive GLUT1 overexpression in the heart which increased both glucose uptake and glycolysis (glucose oxidation was not measured, only phosphocreatine to ATP ratio), resulting in improved contractile function and survival following pressure overload. (*Circulation* 2002;106:2125-2131). Luptak et al. demonstrated that PPARα deficiency in mice reduced the capacity for energy production and caused cardiac dysfunction in response to stress, whereas overexpressing GLUT1 corrected insufficient glucose utilization and oxidation to protect against high workloads (*Circulation* 2005;112:2339-2346). So although increased glucose oxidation was shown to be protective in this study, this finding is in contrast to our study as glucose oxidation was not impaired in normal or hypertrophic LXRα-Tg hearts, whereas this required rescuing in PPARα-deficient hearts. To address these contradictory findings in reference to the abovementioned studies, we have added the following text to the Discussion section:

"In essence, excess glucose uptake and glycolysis appears to be partially uncoupled from mitochondrial oxidation and ATP synthesis in LXRα-Tg hearts, possibly via a regulatory effect of LXRα on Pdk4, which negatively regulates pyruvate dehydrogenase complex (PDC) activity. This is in contrast to previous reports showing that, in the protection against cardiac stress, GLUT1 overexpression corrected insufficient glucose utilization and oxidation caused by PPARα deficiency in

mice (Luptak et al. 2005), and preserved mitochondrial energetic status (Liao et al. 2002). That glucose oxidative capacity is not increased in LXRα-Tg hearts may be due to the fact that mitochondrial oxidation rates are indeed normal and not compromised, and since myocardial contractility is unimpaired is evidence that ATP supply is sufficient to fuel contraction."

Referee #1 (Remarks):

This paper provides interesting data to link LXRa to cardioprotection in the setting of cardiac hypertrophy. The beneficial effect of cardiospecific overexpression of LXR1 is convincing. However, what is less convincing is how LXRa overexpression is having these effects. The metabolic effects of LXRa are opposite to what would be expected. In addition, convincing evidence linking alterations in protein O-GlycNAcylation to decreased cardiac hypertrophy are not convincing. These and other concerns are discussed in detail in the "Comments to Authors".

We are pleased to read that the reviewer finds the data implicating beneficial effects of cardiac-specific overexpression of LXRα convincing. We have attempted to revise the discussion in a manner that better acknowledges and explains the distinctive metabolic effects of LXRα. Increases in glucose uptake in response to hypertrophic perturbation is an established phenomenon, a known adaptation that is protective, and LXRα overexpression is further increasing this capacity. What is paradoxical, albeit interesting, is that we do not observe upregulation of mitochondrial "machinery" that would lead to subsequent increased glucose oxidation rates. On the other hand, one may surmise that increased ATP production is not necessary if myocardial contractile function is adequate, which we demonstrate in LXRα-Tg hearts with invasive hemodynamic monitoring (Suppl. Tables I and II). In overt heart failure, for example, both glucose and fatty acid oxidation are severely diminished. Whether LXRα rescues impaired mitochondrial glucose/fatty acid oxidation in this setting would be the next step in our investigations. However, in the current study we focused on an intermediary or compensated phase of cardiac hypertrophy, and our data indicate that LXRα mediates an adaptive response via glucose-O-GlcNAc-dependent signaling in the protection against pathological hypertrophy.

The mismatch between increased glucose uptake rates relative to glucose oxidation is intriguing, yet has also been previously observed. This apparent paradox has led to an interest in exploring other novel uses for metabolites of glycolysis (reviewed by Kolwicz and Tian, *Cardiovasc Res* 2011;90:194-201; Doenst et al., *Circ Res* 2013;113:709-724). In addition to glycolytic ATP, there are other accessory pathways of glycolysis that affect cell function and survival, such as targeted protein O-GlcNAcylation, which we identified in LXRα-Tg hearts. In the revised manuscript we have further addressed the link between protein O-GlcNAcylation and hypertrophy. As described above, additional *in vitro* experiments show that LXRα overexpression in cardiomyocytes leads to reduced cellular hypertrophy following PE stimulation, but when O-GlcNAc formation is blocked with the inhibitor of the HBP pathway, DON, this effect of LXRα was abolished (Figure 6G and 6H). In addition, more precise experiments were conducted to elucidate downstream effects of O-GlcNAc, and the ANP and BNP

regulatory transcription factors, GATA4 and Mef2c, were identified as O-GlcNAc targets in LXRα-Tg hearts (Figure 8C). This further establishes the link between increased glucose flux and induction of natriuretic peptides with anti-hypertrophic properties. There may in fact be several other protein or enzymatic targets subject to O-GlcNAc modification that contribute to the protective effects of cardiac LXRα. Overall, we believe that these additional data strengthen the link between O-GlcNAcylation and the attenuated cardiac hypertrophy by LXRα overexpression.

Comments from Reviewer 3:

Referee #3 (Comments on Novelty/Model System):

gain and loss of function studies were appropriately performed in mice

Referee #3 (Remarks):

Cannon et.al investigated the role of cardiac LXRα; for pathological cardiac hypertrophy and dysfunction. So far, very little has been known about the role of LXRα in the heart, although its possible effect on blood pressure control has been recently reported. Using a transgenic approach as well as a LXRα-deficient mouse model, the paper indicates that LXRα; acts as a transcriptional regulator that helps in the adaptive metabolic response to chronic cardiac stress by interacting with cardiac metabolism. However, there are several points that need to be more precisely addressed:

We would like to thank the reviewer for his/her careful consideration and evaluation of our manuscript. The remarks and suggestions of the reviewer have certainly helped improve this study, and we believe that the additional work we have performed has strengthened the manuscript considerably.

Major comments:

1) The authors show some robust evidence that overexpression of LXRα; attenuates pathological hypertrophy in a TAC and in an angiotensin-dependent model. Despite the development of a robust phenotype that is protected against pathological remodeling in case of pressure overload, it remains unclear why the authors have decided to study animals only at one time point- namely 5 weeks upon TAC surgery. In a pressure overload model, different phases of cardiac remodeling occur, where different processes take place- early phase (up to 1 week after surgery) where inflammatory processes, apoptosis and necrosis take place, intermediate phase (up to 4 weeks after surgery with development of pathologic hypertrophy), as well as a late phase with cardiac decompensation and development of congestive heart disease. For deciphering of possible complementary mechanisms of LXRα; action, it would be interesting to know whether LXRα; has direct influence on other earlier processes in cardiac remodeling such as inflammation or cardiac apoptosis.

Although we established that increases in glucose-O-GlcNAc-dependent signaling may mediate, in large part, the protective effects of cardiac LXRα, we also agree with the reviewer that other mechanisms are likely to be involved in myocardial protection. We therefore performed an additional TAC experiment of 1 week duration to evaluate a potential role for LXRα in the early phase of hypertrophic remodeling, as suggested by the reviewer. We found that, already at 1 week post TAC, hypertrophy was present and cardiac LXRα overexpression attenuated this development (Suppl. Fig. 1A) and induced changes in the fetal gene program (Suppl. Fig. 1C) that are comparative to the effects observed after 5 weeks TAC.

Supplemental Figure 1A and 1C:

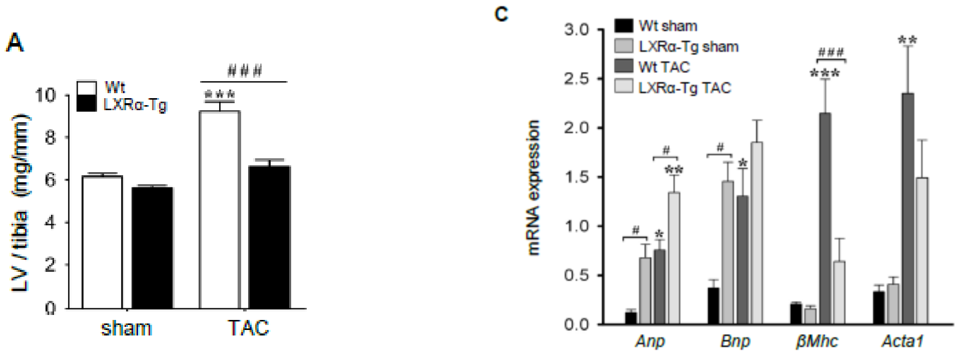

We also observed several inflammatory genes to be strongly upregulated in wild-type mice, but levels of expression were significantly attenuated with cardiac LXRα overexpression. An anti-inflammatory role for LXRα in hypertrophic cardiomyocytes has been previously reported by Wu et al. (*Cardiovasc Res* 2009;84:119-126).

Supplemental Figure 1D-G:

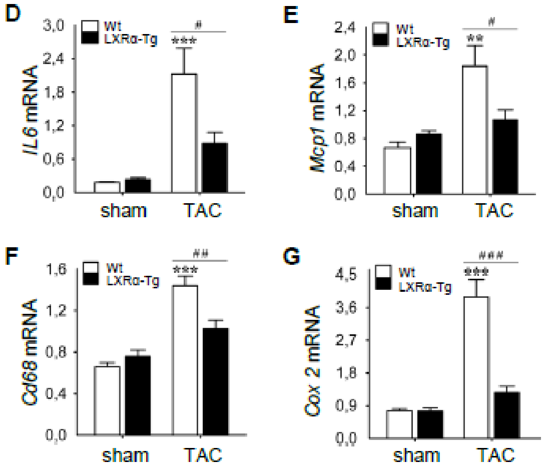

Interestingly, we observed the anti-apoptotic factor, Bcl2, to be significantly increased in LXRα-Tg hearts (Suppl. Fig. 1I and 1J). However, we could not detect downstream cleaved caspase 3 either on Western blot or with immunohistochemistry. In addition, the Bax:Bcl2 ratio was not increased with TAC (Suppl. Fig. 1J), suggesting that apoptosis is not a strong entity in this 1-week TAC study. Nevertheless, cardiac LXRα overexpression may protect against apoptotic triggers.

In summary, it appears that LXRα does influence early inflammatory processes, particularly at the transcriptional level. Since we observed attenuated development of fibrosis in our 5 week TAC study, it is possible that anti-inflammatory effects of LXRα may contribute to this remodeling process.

__Supplemental Figure 1J__:

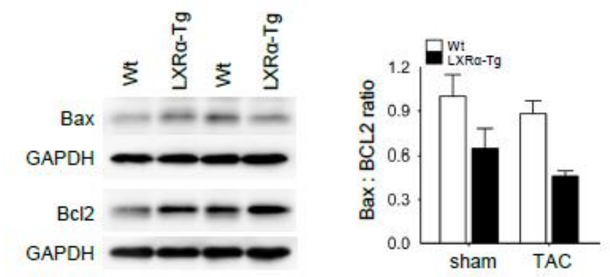

We have added the following text to the Results and Discussion sections, respectively:

"To determine whether cardiac LXRα overexpression affects early hypertrophic remodeling processes, mice were subjected to 1 week of TAC-induced pressure overload. Cardiac hypertrophy was present in Wt mice after 1 week of TAC, however, this was significantly attenuated in LXRα-Tg mice (Suppl. Fig. 1A). Assessment of cardiac function with echocardiography indicated that function remained relatively compensated in TAC-operated mice (Suppl. Fig. 1B). The effect of cardiac LXRα on hypertrophy, including expression of fetal genes (Suppl. Fig. 1C), are comparable to what we observed after 5 weeks TAC. Molecular determinants of inflammation were more strongly upregulated in Wt hearts compared to LXRα-Tg (Suppl. Fig. 1D-G), whereas the anti-apoptotic factor, Bcl2, was significantly induced with LXRα overexpression (Suppl. Fig. 1I and 1J). These data implicate an anti-inflammatory role for LXRα in the initial phase of hypertrophic pathogenesis, and possible protection against anti-apoptotic triggers."

"Cardiac LXRα also appears to influence early remodeling processes since less inflammation in association with decreased hypertrophy occurred at an earlier time point of 1 week post TAC. LXRα-Tg mice may also be less susceptible to apoptosis, which is underscored by upregulation of Bcl2. Taken together, counteraction of inflammatory signaling and myocyte death may explain the attenuated development of fibrosis remodeling we observed after 5 weeks TAC."

2) Whereas the TG mouse model shows a robust cardioprotective response and shows that overexpression of LXRα; is sufficient to induce cardioprotection, the loss-of function model does only show a slightly more severe phenotype than wildtype animals in some but not all aspects (Figure 5A-G). How would the authors comment on that?

We cannot fully explain this apparent discrepancy between LXRα overexpression and deficiency in murine hearts. However, our *in vitro* data indicate that when LXRα is knocked down in isolated cardiomyocytes, there is a sharp increase in cellular growth (Figure 7A) and greater induction of hypertrophic gene expression (Figure 7E and 7F), suggesting that, at the cellular level, there is a clear effect on hypertrophy for cells deficient of LXRα. We therefore speculate that, in the LXRα$^{-/-}$ knockout mouse strain used in our studies, there may be compensatory mechanisms that are operative to

accommodate for the loss of LXRα in the intact heart, for example, a minimal degree of compensation by the LXRβ isoform.

We have addressed this discrepancy in the Discussion section, and added the following text:

"In loss-of-function studies, LXRα$^{-/-}$ mice did not develop significantly greater severity of hypertrophy with respect to WT, although function was worsened in LXRα-deficient hearts. This is in contrast to a previous report showing exacerbated hypertrophic response in LXRα$^{-/-}$ mice (Wu et al. 2009). The discrepancy between LXRα overexpression and deficiency cannot be fully explained herein, however, our in vitro data indicate that, at the cellular level, there is a clear effect on hypertrophic growth in cardiomyocytes lacking LXRα, and thus compensatory mechanisms may be operative in the intact heart."

3) Figure 6D: The authors argue that the cardioprotective effect of LXRα; is to be contributed to the stimulation of HBP. Indeed the Western Blot they show shows an increased glycosylation with increased MOIs of Ad- LXRα; however the DON administration does not seem to lead to a significant reduction of the O-Glycosylation pattern of the transfected cells. Furthermore, instead of PE another positive control (such as treatment with Glucosamine oder Thiamet G) is more proper in this setting. Please provide the proper controls.

Firstly, we agree that the increased global O-GlcNAcylation in cells overexpressing LXRα is only partially attenuated by DON. Although a higher dose of DON may have yielded a greater reduction in O-GlcNAc signaling, DON is relatively toxic when administered at these higher dose ranges. Despite only partially attenuated O-GlcNAc protein expression, we nevertheless found 100 µM DON treatment sufficient to exert other effects, namely on modulation of gene expression (Figure 6E and 6F) and cell size (Figure 6G and 6H, new data).

The following assessment of cell size has been added to the revised manuscript:

Figure 6G and 6H:

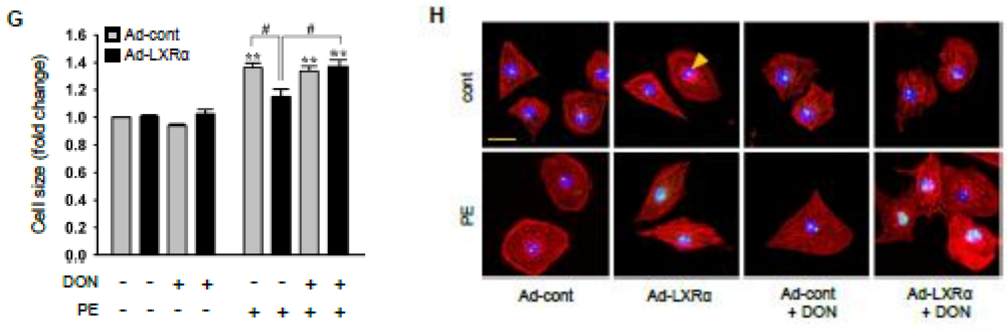

"Further assessment of cellular hypertrophy indicated that DON inhibition of HBP flux also abolished the Ad-LXRα-mediated reductions in cell size that was increased upon PE stimulation (Figure 6G and 6H)."

Secondly, we had included in the supplementary figures section of the original data supplement an additional Western blot detecting O-GlcNAc protein levels in the presence of PUGNAc. PUGNAc has a mode of action similar to Thiamet G in that it inhibits O-GlcNAcase, the enzyme responsible for O-GlcNAc removal from proteins (Suppl. Figure 9B). We show that O-GlcNAc expression in isolated cardiomyocytes is indeed increased in the presence of PUGNAc. Furthermore, part of this Western blot was incubated in *N*-acetylglucosamine to demonstrate O-GlcNAc antibody specificity (Suppl. Figure 9B). Here, we show that the O-GlcNAc signal was strongly attenuated:

<u>Supplemental Figure 9B</u>:

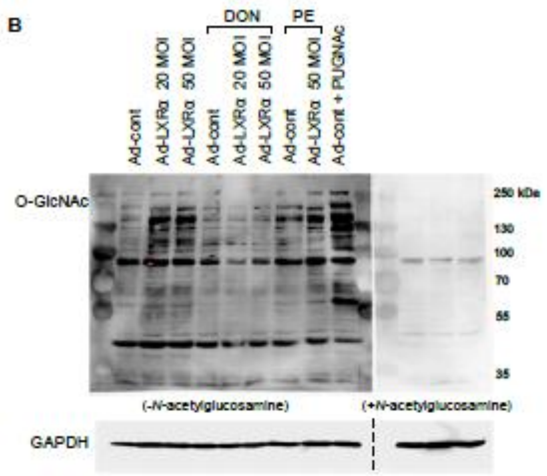

4) Indeed, in case the cardioprotective effect of LXRα; is HBP-dependent, one would expect a deterioration of the cardioprotective function after DON treatment in vivo. Is there any in vivo data to confirm this?

We have searched the literature for studies regarding *in vivo* intervention in the HBP pathway, such as with 6-Diazo-5-oxo-L-norleucine (DON) treatment, but have found no such studies. One reason may be that DON inhibition may be relatively toxic at physiological doses. On the other hand, a seminal study conducted by Watson et al. investigated the effect of O-GlcNAc transferase (OGT) deletion in rats (*Proc Natl Acad Sci USA* 2010;107:17797-17802). OGT is the enzyme that links O-GlcNAc onto protein residues. Rats with cardiomyocyte-specific deletion of OGT subjected to myocardial infarction exhibited worsened cardiac function and adverse structural remodeling, indicating that this pathway is indeed crucial for protection against heart failure development.

Minor comments:

1) It would be helpful to provide the n = number of animals per group directly in the figure itself and not only in the figure legend

We agree that disclosing the number of animals per group directly in the figure would be more helpful, however, we are of the opinion that because we have created multi-panel figures, the additional inclusions would cause overcrowding and be distracting to the reader. Should the editorial office request otherwise, we shall certainly comply. We have instead specified the exact number of animals per group in the legend, as opposed to a range.

2) The authors argue that O-Glycosylation and the shift towards glucose reliance is believed to be an adaptive response that confers cardioprotection, however , it is also known that increased O-glycosylation of certain proteins in the heart (Eriksson et al, 2013) also may have an unfavorable effect on heart function, arrhythmias and overall animal survival. Hence, it would be informative to have the Kaplan-Meyer curves of overall survival of the TG vs WT mice

We agree that global O-GlcNAcylation is indicative of numerous proteins being O-GlcNAcylated, and that it can be assumed that there are indeed unfavorable glycosylation effects. Regulation of the balance between favorable versus unfavorable effects may be key in the overall impact of O-GlcNAc signaling in the diseased myocardium. Also, identification of specific proteins that are O-GlcNAcylated should provide further insight into the link between glucose signaling and the diseased heart. We acknowledged this limitation of the original manuscript and have since then performed additional experiments to identify O-GlcNAcylated targets (please refer to major comment #3 of our responses to reviewer #1 for these results).

Regarding survival, in our pressure overload model induced via transverse aortic constriction (TAC), we experienced low mortality rates that were connected to mainly technical issues, i.e.: intubation, surgery, failure to recover from anesthesia. Mice died either during surgery or shortly thereafter, within 24 hours. Mice that survived surgery were able to withstand chronic pressure overload over the 5 week period, irrespective of genotype. We speculate that prolongation of TAC past 8 weeks may incur a more severe heart failure phenotype, and therefore later time points would yield more information regarding survival in these mice. In the relatively short-term study of angiotensin II (Ang II) infusion, a total of 6 mice died or reached humane end point (loss of > 15% body weight, hind limb paralysis).

We have instead provided a Table indicating mortality with respect to genotype:

Mortality from TAC and angiotensin II intervention

Intervention	Wt	LXRα-Tg
TAC (5 weeks)	***n=59***	***n=61***
**Dead during surgery**	**6**	**2**
**Dead 1 hr post surgery**	**1**	
**Dead 1 day post surgery**	**2**	
Angiotensin II infusion (10 days)	***n=20***	***n=26***
**Dead 48 hrs post pump insertion**		**2**
**Dead 3-6 days post pump insertion**	**2**	**2**

3) Fig. 6E-F: is the labeling correct below the last column. Should this not read DON+PE?

Thank you for bringing this to our attention. We have corrected the legend: DON+PE.

2nd Editorial Decision 08 May 2015

Thank you for the submission of your revised manuscript to EMBO Molecular Medicine. We have now received the enclosed reports from the referees that were asked to re-assess it. As you will see the reviewers are now globally supportive and I am pleased to inform you that we will be able to accept your manuscript pending the following final amendments:

1) As per our Author Guidelines, the description of all reported data that includes statistical testing must state the name of the statistical test used to generate error bars and P values, the number (n) of independent experiments underlying each data point (not replicate measures of one sample), and the actual P value for each test (not merely 'significant' or 'P < 0.05'). I note that you have provided the exact P value in some but not all cases.

2) Every published paper now includes a 'Synopsis' to further enhance discoverability. Synopses are displayed on the journal webpage and are freely accessible to all readers. They include a short standfirst as well as 2-5 one sentence bullet points that summarise the paper. Please provide the synopsis including the short list of bullet points that summarise the key NEW findings. The bullet points should be designed to be complementary to the abstract - i.e. not repeat the same text. We encourage inclusion of key acronyms and quantitative information. Please use the passive voice. Please attach this information in a separate file or send them by email, we will incorporate it accordingly. You are also welcome to suggest a striking image or visual abstract to illustrate your article. If you do please provide a jpeg file 550 px-wide x 400-px high.

3) We are now encouraging the publication of source data, particularly for electrophoretic gels and blots, with the aim of making primary data more accessible and transparent to the reader. Would you be willing to provide a PDF file per figure that contains the original, uncropped and unprocessed scans of all or at least the key gels used in the manuscript? The PDF files should be labeled with the appropriate figure/panel number, and should have molecular weight markers; further annotation may be useful but is not essential. The PDF files will be published online with the article as supplementary "Source Data" files. If you have any questions regarding this just contact me.

4) Please improve the quality of Fig.6, panel D, I see some digital disturbance in some bands (reddish coloring).

I look forward to reading a new revised version of your manuscript as soon as possible.

***** Reviewer's comments *****

Referee #1 (Remarks):

none

Referee #3 (Remarks):

well done

2nd Revision - authors' response 04 June 2015

We are very pleased to hear that our manuscript entitled "Cardiac LXRα protects against pathological cardiac hypertrophy and dysfunction by enhancing glucose uptake and utilization," (EMM-2014-04669-V2) has been accepted for publication in EMBO Molecular Medicine pending final amendments.

As requested, we now provide exact P values in the figure legends. We did, however, have a few P values that were low (indicating a very high level of significance): for all values that were P < 1 X 10-7, we chose to express them as P < 0.00001. Please inform us should this manner not be acceptable for presentation.

In addition, we have included a synopsis with the revised manuscript, as well as a Source Data file containing all original blots presented in the manuscript.

Lastly, the quality of Fig. 6, panel D, has been improved by converting the image from "color" to "grayscale" to remove the reddish coloring.

© EMBO
