# Supplementary material for: Cardiac LXRα protects against pathological cardiac hypertrophy and dysfunction by enhancing glucose uptake and utilization
Source: EMBO Mol Med. 2015 Jul 14;7(9):1229–43. doi: 10.15252/emmm.201404669 (PMC4568954; doi:10.15252/emmm.201404669)

Full unedited blots for Figure 8, panel A

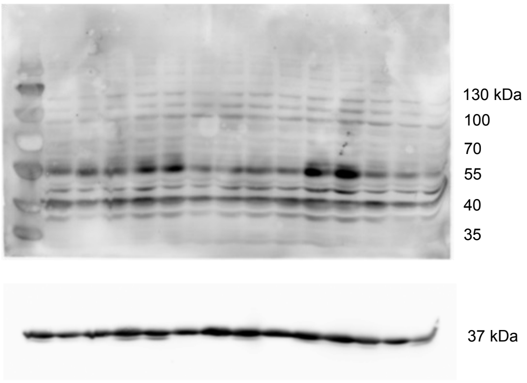

Full unedited blots for Figure 8, panel B

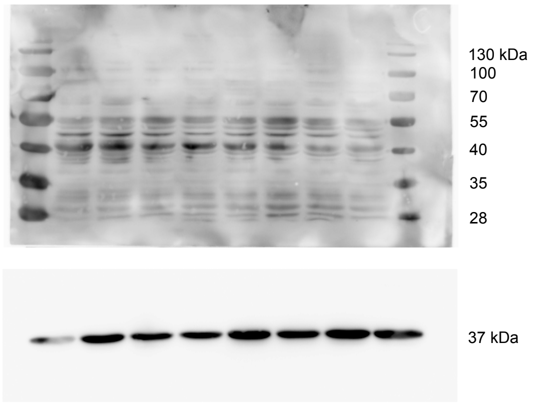

Full unedited blots for Figure 8, panel C

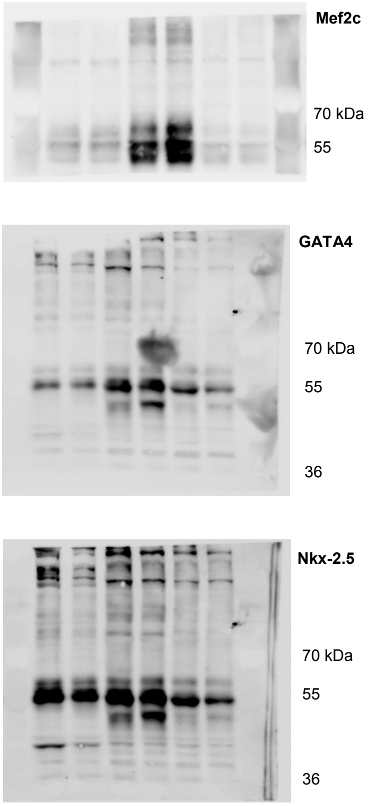

Supplement: Supplementary file 11 [file emmm0007-1229-sd11.pdf]
